# Comparison of *Francisella tularensis *genomes reveals evolutionary events associated with the emergence of human pathogenic strains

**DOI:** 10.1186/gb-2007-8-6-r102

**Published:** 2007-06-05

**Authors:** Laurence Rohmer, Christine Fong, Simone Abmayr, Michael Wasnick, Theodore J Larson Freeman, Matthew Radey, Tina Guina, Kerstin Svensson, Hillary S Hayden, Michael Jacobs, Larry A Gallagher, Colin Manoil, Robert K Ernst, Becky Drees, Danielle Buckley, Eric Haugen, Donald Bovee, Yang Zhou, Jean Chang, Ruth Levy, Regina Lim, Will Gillett, Don Guenthener, Allison Kang, Scott A Shaffer, Greg Taylor, Jinzhi Chen, Byron Gallis, David A D'Argenio, Mats Forsman, Maynard V Olson, David R Goodlett, Rajinder Kaul, Samuel I Miller, Mitchell J Brittnacher

**Affiliations:** 1 Department of Genome Sciences, University of Washington, Campus Box 357710, 1705 NE Pacific street Seattle, Washington 98195, USA; 2 Department of Pediatrics, Division of Infectious Diseases, University of Washington, Campus Box 357710, 1720 NE Pacific street, Seattle, Washington 98195, USA; 3 NBC Analysis, Division of NBC Defence, Swedish Defence Research Agency, SE-901 82 Umeå, Sweden; 4 Department of Clinical Microbiology, Infectious Diseases, Umeå University, SE-901 85 Umeå, Sweden; 5 University of Washington Genome Center, University of Washington, Campus Box 352145, Mason Road, Seattle, Washington 98195, USA; 6 Department Medicine, University of Washington, Seattle, Washington 98195, USA; 7 Department of Microbiology, University of Washington, Box 357242, 1720 NE Pacific street, Seattle, Washington 98195, USA; 8 Department of Medicinal Chemistry, Box 357610, University of Washington, Seattle, Washington 98195, USA

## Abstract

**Background:**

*Francisella tularensis *subspecies *tularensis *and *holarctica *are pathogenic to humans, whereas the two other subspecies, *novicida *and *mediasiatica*, rarely cause disease. To uncover the factors that allow subspecies *tularensis *and *holarctica *to be pathogenic to humans, we compared their genome sequences with the genome sequence of *Francisella tularensis *subspecies *novicida *U112, which is nonpathogenic to humans.

**Results:**

Comparison of the genomes of human pathogenic *Francisella *strains with the genome of U112 identifies genes specific to the human pathogenic strains and reveals pseudogenes that previously were unidentified. In addition, this analysis provides a coarse chronology of the evolutionary events that took place during the emergence of the human pathogenic strains. Genomic rearrangements at the level of insertion sequences (IS elements), point mutations, and small indels took place in the human pathogenic strains during and after differentiation from the nonpathogenic strain, resulting in gene inactivation.

**Conclusion:**

The chronology of events suggests a substantial role for genetic drift in the formation of pseudogenes in *Francisella *genomes. Mutations that occurred early in the evolution, however, might have been fixed in the population either because of evolutionary bottlenecks or because they were pathoadaptive (beneficial in the context of infection). Because the structure of *Francisella *genomes is similar to that of the genomes of other emerging or highly pathogenic bacteria, this evolutionary scenario may be shared by pathogens from other species.

## Abstract 2

.Sequencing of the non-pathogenic *Francisella tularensis *sub-species novicida U112, and comparison with two pathogenic sub-species, provides insights into the evolution of pathogenicity in these species.

## Background

The genomes of bacterial pathogens are constantly evolving through various processes. The acquisition of genes that promote virulence by lateral transfer is a common property of pathogens [1,2]. The acquisition of additional virulence factors or pathogenicity islands can alter a pathogen's virulence or host range, or both. For example, the diseases caused by pathogenic *Escherichia coli *strains can take very diverse forms, depending on the virulence factors encoded in the locus of enterocyte effacement present in their genomes [3]. In addition to gain of function by gene acquisition, loss of function has also been postulated to play a role in evolution toward greater pathogenicity and host adaptation. Indeed, highly pathogenic strains tend to harbor numerous pseudogenes, whereas related strains that are mildly pathogenic do not. Comparison of *Burkholderia *and *Bordetella *genomes suggests that loss of function contributes to host adaptation [4,5]. In practice, few occurrences of fixed loss of function have been demonstrated to be beneficial for virulence [6,7]. It is therefore probable that many of the pseudogenes are merely the result of lack of selection for functions that are not needed in the host environment or of evolutionary bottlenecks [8-11].

One mechanism that promotes accelerated gene loss in pathogens may be the insertion of insertion sequences (IS elements). Analyses of genomes of some virulent strains have revealed numerous IS elements and rearrangements. In many genome comparisons with free-living or less virulent strains, a correlation between IS elements, pseudogenes, and genomic rearrangements has been observed. In *Shigella flexneri *for instance, IS elements have disrupted one-third of all genes annotated as pseudogenes [12]. Based on this observation and other comparisons [4,12-16], it has been proposed that the proliferation of IS elements is the cause of a large number of pseudogenes and genomic rearrangements in emerging or highly virulent pathogens. Given the fact that many highly virulent and emerging pathogens share these genomic features [4,12-16], it is important to understand and establish the relationship (if any) between gene acquisition, IS elements, pseudogenes, and genomic rearrangements.

In order to examine in detail the genetic determinants and the evolutionary processes involved in the emergence of *Francisella *human pathogenic strains, we compared the genomes for human pathogenic strains with the genome of a strain that is not pathogenic to humans, namely *Francisella tularensis *subspecies *novicida *U112. The facultative intracellular pathogen *Francisella tularensis *causes the zoonotic disease tularemia in a wide range of animals. Four subspecies of this Gram-negative organism are recognized: *holarctica*, *tularensis*, *novicida*, and *mediasiatica*. Subspecies *tularensis *is extremely infectious in humans; as few as ten colony-forming units can cause a successful infection that can be lethal if it is not treated. Subspecies *holarctica *causes a milder disease, which is also known as tularemia [17]. The subspecies *novicida *diverged from an ancestor common to the subspecies *tularensis *and *holarctica *[18]. Subspecies *novicida *is not infectious in humans but it causes a disease in mice that is very similar to tularemia, and it can replicate within human macrophages *in vitro *[19]. A few cases of human infection with subspecies *novicida *have also been reported in immunodeficient patients [20,21]. Similar virulence strategies are used by the various subspecies [22,23], although subspecies-specific factors must determine differences in host range and infectivity.

The genomes of *holarctica *and *tularensis *strains both exhibit properties similar to those of other highly virulent pathogens [16,24,25]: high IS element content, numerous genomic rearrangements, and a high number of pseudogenes. A two-way comparison between a *holarctica *and a *tularensis *strain revealed a strikingly different genome organization between them, mediated by ISFtu1 and ISFtu2 [16]. Since both strains are pathogenic to humans, this comparison could not be used to investigate the factors that enable these strains to infect humans. Such an investigation became possible with the genome sequence and annotation of *F t novicida *U112. In contrast to the *F tularensis *strains already sequenced, *F t novicida *U112 belongs to a subspecies that diverged from a common ancestor before the divergence of the two human pathogenic subspecies. Using the sequence of the genome of U112, we looked in particular for acquired sequences and genomic rearrangements that would have occurred before divergence of the subspecies *tularensis *and *holarctica*. The comparison of the genome of U112 with the genomes of *F t tularensis *Schu S4 and *F t holarctica *LVS (live vaccine strain) allowed us to determine the evolutionary processes that potentially contributed to the ability of *tularensis *and *holarctica *strains to infect humans. In addition, it shed some light on the relationships between pseudogenes, IS elements, and genomic rearrangements. The annotation of the strain U112 genome also provides a foundation for systematic genome-scale studies of *Francisella *virulence and related processes using a wild-type organism that does not require high-level laboratory containment. Major attributes of *F tularensis *virulence have already been uncovered using the strain U112 [26-30], in advance of confirmation using human virulent bacteria.

## Results and discussion

### Genomic rearrangements at the level of IS elements repeatedly took place in the human pathogenic strains but seldom in *F t novicida *U112

#### The genomic nucleotide sequence is highly conserved between the three strains but different mutation rates are apparent

We compared the newly sequenced genome of *F t *subspecies *novicida *strain U112 with the published sequence of the genomes of *F t *subspecies *tularensis *strain Schu S4 [25] and that of *F t *subspecies *holarctica *strain LVS (Chain and coworkers, unpublished data). Some general properties and features of the three genomes are summarized in Table 1, in which the extent of the similarity between the three subspecies is apparent. The genome of U112 is 17 kilobases (kb) larger than the Schu S4 genome and 14 kb larger than the genome of LVS. Few strain-specific regions were detected in this three-way comparison: the genome of U112 carries about 240 kb of sequences not found in the two other strains; the genome of Schu S4 carries 17.3 kb of strain-specific regions; and the genome of LVS does not contain any specific regions. The origin of replication of the U112 chromosome (around position 1) was predicted according to one of the switching points of the GC skew and by searching for DnaA-binding sequences. It is consistent with the predicted origin of replication of the chromosomes of Schu S4 and LVS, suggesting a common genome backbone for the three subspecies. The estimated nucleotide sequence identity is 97.8% between the sequences common to the U112 and the LVS genomes, 98.1% between the sequences common to U112 and Schu S4, and 99.2% between the sequence common to Schu S4 and LVS. The proposition based on physiologic experiments and DNA-DNA re-association [20] that *novicida *may be classified as a subspecies of *tularensis *is supported by the nucleotide identity between genomes.

**Table 1:** The general properties of the genomes are compared

Property	Strain (subspecies)		
	
	U112 (*novicida*)	Schu S4 (*tularensis*)	LVS (*holarctica*)
Size (base pairs)	1,910,031	1,892,819	1,895,998
GC content (%)	32.47	32.26	32.15
Protein coding genes	1731	1445	1380
Pseudogenes	14	254	303
ISFtu1 or remnant	1	53	59
ISFtu2 or remnant	18	18	43
ISFtu3 or remnant	4	3	3
ISFtu4 or remnant	1	1	1
ISFtu5 or remnant	0	1	1
ISFtu6 or remnant	2	3	2
Source (year, place)	Water (1950, Utah)	Human (1941, Ohio)	Live vaccine strain (ca. 1930, Russia)

LVS, live vaccine strain.

Although no official genomic criteria exists to classify strains into species, Konstantinidis and coworkers [31] found that almost all 70 strains in their study set that reside in the same species exhibited greater than 94% average nucleotide identity (ANI). They also showed that the classification based on ANI correlates with classifications performed with 16S RNA sequences, DNA-DNA re-association, and mutation rate. In comparison, the few sequences of the other *Francisella *species available in Genbank, namely *Francisella philomiragia*, exhibit an ANI of 91.66% with the genome of U112. The ANI corroborates the proposition that *novicida *arose by diverging from an ancestor common to the subspecies *tularensis *and *holarctica*, and that the subspecies *tularensis *and *holarctica *subsequently diverged from a common ancestor [31,32]. Based on the average level of nucleotide identity between the three genomes, it is possible to estimate the rate of substitution in the genomes of *holarctica *and *tularensis *after their divergence. The genomes of *holarctica *strains are estimated to have evolved at an average rate of 0.55 base pairs (bp)/100 bp from the common ancestor, whereas the genome of Schu S4 diverged at the lower rate of 0.25 bp/100 bp.

#### Genome reorganization occurred in the human pathogenic *F tularensis *ancestral strain during or after differentiation from the nonpathogenic strain

A recent study using paired-end sequencing [24] indicated that the organization of the genomes of *holarctica *strains and *tularensis *strains is not conserved. However, the organization was highly similar for the genomes of the 67 *holarctica *strains analyzed. Similarly, the genome of *holarctica *strain OSU18 is collinear with the genome of the *holarctica *strain LVS, but it is organized differently than the genome of Schu S4 [16]. These findings extend the phylogenetic and molecular evidence that the strains are mostly clonal in the subspecies *holarctica *and that their genome is relatively stable [18,32-34]. The subspecies *tularensis *can be divided into two distinct groups (type AI and AII) [18,35]. According to amplified fragment length polymorphism and restriction fragment length polymorphism analyses, genomes in the subspecies *tularensis *are organized differently but are similar within groups [33,34]. Hence, the genome of LVS is representative of all genomes in the subspecies *holarctica*, whereas the genome of Schu S4 represents genomes in the type AI group.

**Figure 1:**
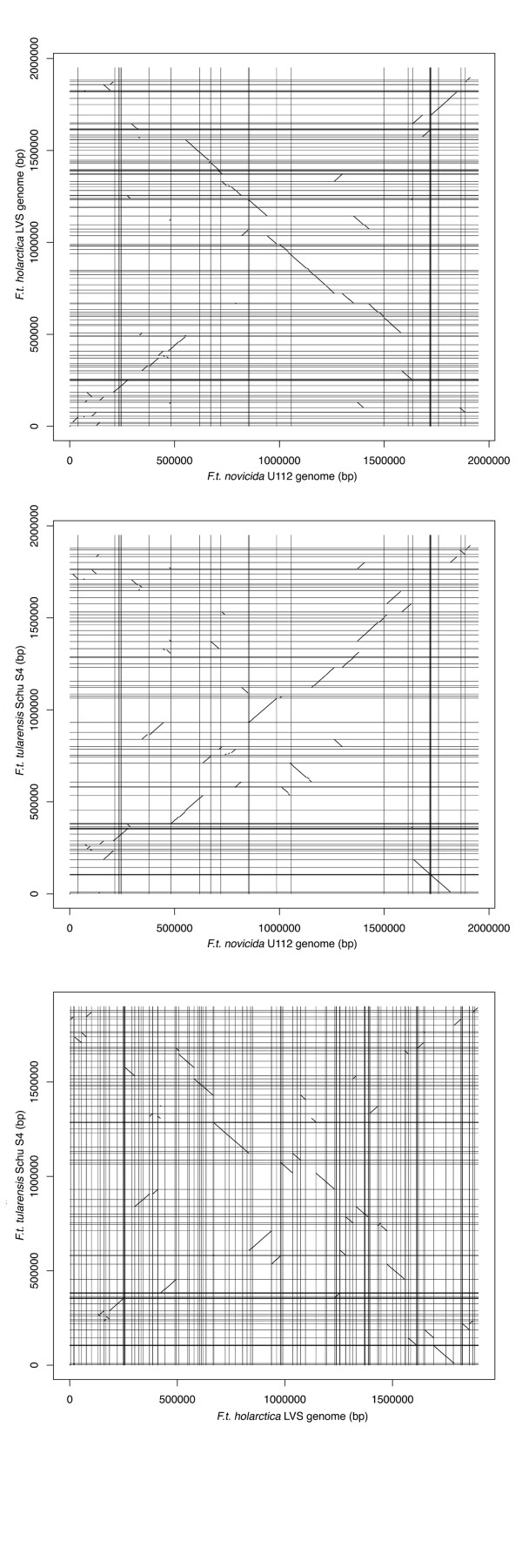
The alignment of the genomes reveals multiple genomic rearrangements probably mediated by IS elements. Each genome was aligned against each of the others using Nucmer (see Materials and methods). Horizontal and vertical lines represent the location of the IS elements in the compared genomes. The breakpoints of the syntenic blocks in the subspecies *holarctica *and *tularensis *are often associated with IS elements, whereas IS elements do not border most syntenic blocks in the genome of *novicida*. bp, base pairs; *F.t*., *Francisella tularensis*; IS, insertion sequences; LVS, live vaccine strain.

Sequence alignment of the U112 and Schu S4 genomes reveals 59 chromosomal segments with the same gene content and gene order in both organisms, but arranged differently throughout both genomes (Figure 1). Chromosomal segments with the same gene content and gene order in two bacterial genomes are hereafter termed 'syntenic regions'. The discrepancy in the order of the chromosomal segments between the two genomes suggests that regions have been moved, in one genome or the other. Hence, there are a total of 118 genomic breakpoints when comparing the two genomes. Similarly, 59 syntenic regions are arranged differently when comparing the genomes of U112 and LVS, and 51 are arranged differently between the genomes of Schu S4 and LVS (Figure 1), which is the same amount as found when comparing Schu S4 and OSU18 genomes [16]. Twenty-eight out of the 59 syntenic blocks (47%) are nearly identical in the genomes of Schu S4 and LVS relative to the genome of U112. However, the order in which the blocks are arranged differs greatly. This suggests that these syntenic blocks formed before differentiation between both human pathogenic subspecies, but moved independently later in one or both genomes. The rest of the syntenic blocks in LVS and Schu S4, in comparison with U112, differ both in content and order (Figure 1), which suggests that they formed after differentiation of the two subspecies.

#### Localization of IS elements at genomic breakpoints suggests that IS elements are involved in most genomic rearrangements in the human pathogenic strains

Six types of IS elements were identified in the three genomes. Five of them are present in the three genomes at least in a remnant form, whereas one, ISFtu5, is only present in the subspecies *holarctica *and *tularensis*. As shown in Table 1, the number of each IS element varies greatly in the three strains. The difference in numbers of ISFtu1 and ISFtu2 elements is particularly large. It suggests that ISFtu1 has transposed and proliferated in the genomes of the subspecies *tularensis *and *holarctica*, or in the genome of their common ancestor. ISFtu2 exhibits more proliferation in the *holarctica *genome. ISFtu1 appears to have been replicated essentially in the ancestor of *holarctica *and *tularensis *strains becuase 46 out of 53 elements are bordered by the same sequences in both genomes. Nine ISFtu1 elements exhibit the same bordering regions on both sides in the two subspecies genomes. However, 37 other ISFtu1 elements share only one side with an element in the other genome, indicating rearrangements specific to each subspecies. About 13 ISFtu2 elements may have transposed in the ancestral genome of *tularensis *and *holarctica*, as indicated by common bordering sequences, but have undergone subsequent rearrangements because ten ISFtu2 elements have only one common side.

These findings strongly support the proposition that genomic rearrangements occurred in the genomes of the *tularensis *and *holarctica *strains by homologous recombination at ISFtu1 and ISFtu2 elements [16]. This proposition is also supported by the fact that 82% of breakpoints of LVS-Schu S4 syntenic blocks are bordered by an IS element within 100 bp (Figure 1). Similarly, 60% of the breakpoints in LVS-U112 and Schu S4-U112 syntenic blocks are bordered by IS elements in the genome of the human pathogenic subspecies (Figure 1). This lower incidence may be due to transposition of IS elements subsequent to the initial rearrangement. IS elements appear to play a prominent role in rearrangement events, further corroborating that these events took place in the ancestor of *holarctica *and *tularensis*. Indeed, 88% of the Schu S4-U112 syntenic blocks are bordered by an IS element at one extremity or both in the genome of Schu S4. On the other hand, the location of IS elements in the genome of U112 exhibits association with breakpoints for merely four ISFtu2 elements. This suggests that the IS elements did not play a prominent role in the evolution of the strains that are not pathogenic to humans.

In summary, comparative analysis using the genome of U112 revealed that the complex evolutionary scenario of the three *F tularensis *subspecies involves the transposition of ISFtu1 (*tularensis *and *holarctica*) and ISFtu2 (*novicida*, *tularensis*, and *holarctica*), accompanied by replication of these elements and genomic rearrangements at the location of these elements at distinct steps in genome evolution.

### Comparison with the *novicida *genome identifies genes specific to the human pathogenic strains and reveals pseudogenes not previously uncovered in their respective genomes

#### The gene content of *F t novicida *U112 reveals a species genome backbone

In the genome of U112, 1,731 protein-coding genes, 14 pseudogenes, and seven disrupted genes encoding an IS element transposase were identified. The coding regions (1,751,817 bp) represent 91.72% of the entire genome. Thirty-eight tRNA genes were identified, representing 30 anticodons encoding the 20 amino acids as well as three operons encoding the 5S, 16S, and 23S ribosomal RNAs and tRNAs for alanine and isoleucine. The same RNA genes and operons are found in the genomes of *tularensis *and *holarctica*. Overall, 1,813 distinct genes (excluding IS element genes and 33 hypothetical genes that we believe are noncoding) were found in at least one of the three genomes. Out of these 1,813 genes, a total of 1,572 gene sequences (functional or disrupted) are common to the three genomes. Hence, the core gene set may represent about 86.4% of all distinct genes identified in the three genomes (Additional data file 1).

#### Human pathogenic strains contain genes that are absent from the nonpathogenic strain U112

In addition to this core gene set, the genomes of LVS and Schu S4 contain 41 genes whose sequences are absent from the genome of U112, and thus may play an important role in the virulence of *holarctica *and *tularensis *for humans. Thirteen are single genes found within sequences common to the three subspecies, and the remaining 28 are distributed in specific regions containing two to six genes (Table 2). Even a small number of acquired genes can cause specific differences in pathogenicity [36]. It is interesting that U112 is not virulent for humans but is nonetheless able to colonize human macrophages *in vitro*. This indicates that the strain encodes virulence factors that are important for the infection of human macrophages but that it lacks specific factors that make human infection possible for the *holarctica *and *tularensis *strains. Hence, it is possible that some of the 41 genes that are specific to human pathogenic strains but are lacking in U112 could confer the ability to infect humans. The genome of Schu S4 contains nine additional protein encoding genes and two pseudogenes (Table 3) that are absent from the other genomes, which reduces the list of known *tularensis *specific genes [37,38]. An 11.1 kb region (FTT1066-FTT1073) has been shown to be present in all the strains of the subspecies *tularensis *and was named RD8 [37]. It is possible that some of these specific genes contribute to the greater virulence of the *tularensis *strains compared with the *holarctica *strains. In addition to specific genes, the genome of Schu S4 contains 20 duplicated genes and the genome of LVS has 34 duplicated genes, found as single copies in the genome of U112. Because they are identical copies, the duplicated genes could be responsible for a novel gene expression pattern and could therefore represent a gain of function for the human pathogenic strains.

**Table 2:** Functions specific to human-pathogenic strains (*holarctica *and *tularensis*)

	Locus tag in the genome of Schu S4^2^	Locus tag in the genome of LVS^a^	Size of the predicted protein (amino acids)	G+C content (%)	Gene name^a^	Gene product description^a^	Functional category^b^
Sequences specific to human pathogenic strains	FTT0016	FTL_1849	192	30.0	-	Hypothetical protein FTT0016	Hypothetical
	
	FTT0300	FTL_0211	284	27.4	-	Hypothetical protein FTT0300	Hypothetical
	FTT0301	FTL_0212	289	29.5	-	Hypothetical protein FTT0301	Hypothetical
	
	FTT0376c	FTL_1314	352	28.1	-	Hypothetical membrane protein	Hypothetical
	
	FTT0395	FTL_0415	237	29.3	-	Hypothetical protein FTT0395	Hypothetical
	
	FTT0430	FTL_0461	144	34.6	*speH*	S-adenosylmethionine decarboxylase	Other metabolism
	FTT0431	FTL_0499	289	33.1	*speE*	Spermidine synthase	Other metabolism
	FTT0434	FTL_0500	328	33.7	-	Hypothetical protein FTT0434	Other metabolism
	
	FTT0524	FTL_0977	128	28.4	-	Hypothetical protein FTT0524	Hypothetical
	
	FTT0572	FTL_1339	484	31.5	-	Proton-dependent oligopeptide transport (POT) family protein	Transport
	
	FTT0601	FTL_0780	39	31.6	-	Hypothetical protein FTT0601	Hypothetical
	FTT0602c	FTL_0867	492	31.1	-	Hypothetical protein FTT0602c	Hypothetical
	FTT0603	FTL_0870	59	30.3	-	Hypothetical protein FTT0603	Hypothetical
	FTT0604	FTL_0872	144	31.2	-	Hypothetical protein FTT0604	Hypothetical
	
	FTT0727	FTL_1512	226	29.4	-	Hypothetical protein FTT0727	Hypothetical
	FTT0728	FTL_1513	310	33.2	*ybhF*	ABC transporter, ATP-binding protein	Transport
	FTT0729	FTL_1515	372	30.4	*ybhR*	ABC transporter, membrane protein	Transport
	
	FTT0794	FTL_1427	428	30.3	-	Hypothetical protein FTT0794	Hypothetical
	FTT0795	FTL_1426	227	25.5	-	Hypothetical protein FTT0795	Hypothetical
	FTT0796	FTL_1425	253	23.2	-	Hypothetical protein FTT0796	Hypothetical
	
	FTT0958c	FTL_1245	235	33.2	-	Short chain dehydrogenase	Cell wall/LPS/capsule
	
	FTT1079c	FTL_1123	86	37.3	-	Hypothetical protein FTT1079c	Hypothetical
	
	FTT1172c	FTL_0777	143	29.4	*csp*	Cold shock protein (DNA binding)	Signal transduction and regulation
	FTT1174c	FTL_0776	69	24.5	-	Hypothetical protein FTT1174c	Hypothetical
	FTT1175c	FTL_0759	212	25.5	-	Hypothetical membrane protein	Hypothetical
	FTT1188	FTL_0668	211	28.8	-	Hypothetical membrane protein	Hypothetical
	
	FTT1307c	FTL_0211	178	34.5	-	Hypothetical protein FTT1307c	Hypothetical
	
	FTT1395c	FTL_0605	476	30.6	-	ATP-dependent DNA helicase	Signal transduction and regulation
	
	FTT1451c	FTL_0604	294	38.4	*wbtL*	Glucose-1-phosphate thymidylyltransferase	Cell wall/LPS/capsule
	FTT1452c	FTL_0603	286	29.4	*wbtK*	Glycosyltransferase	Cell wall/LPS/capsule
	FTT1453c	FTL_0602	495	30.1	*wzx*	O-antigen flippase	Cell wall/LPS/capsule
	FTT1454c	FTL_0598	241	28.9	*wbtJ*	Hypothetical protein FTT1454c	Cell wall/LPS/capsule
	FTT1458c	FTL_0594	409	22.2	*wzy*	Membrane protein/O-antigen protein	Cell wall/LPS/capsule
	FTT1462c	FTL_0527	263	29.7	*wbtC*	UDP-glucose 4-epimerase	Cell wall/LPS/capsule
	
	FTT1581c	FTL_0511	94	28.5	-	Endonuclease	Mobile and extrachromosomal element functions
	
	FTT1594	FTL_1634	330	30.8	-	Transcriptional regulator, LysR family	Signal transduction and regulation
	FTT1595	FTL_1633	51	26.9	-	Hypothetical protein FTT1595	Hypothetical
	FTT1596	FTL_1632	132	32.1	-	Hypothetical protein FTT1596	Hypothetical
	FTT1597	FTL_1631	485	30.3	-	Hypothetical protein FTT1597	Hypothetical
	
	FTT1614c	FTL_0502	227	31.6	-	Hypothetical protein FTT1614c	Hypothetical
	FTT1659	FTL_0034	341	26.0	-	Hypothetical protein FTT1659	Hypothetical

Genes inactivated in *novicida *but functional in human pathogenic strains	FTT0707	FTL_1529	264	26.9	-	Nicotinamide mononucleotide transport (NMT) family protein	Transport
	FTT1090	FTL_1113	225	27.6	-	Hypothetical protein	Hypothetical
	FTT1076	FTL_1125	424	31.1	*hipA*	Transcription regulator	Signal transduction and regulation
	FTT0666c	FTL_0940	193	29.5	-	Methylpurine-DNA glycosylase family protein	DNA metabolism
	FTT1450c	FTL_0606	348	33.6	*wbtM*	dTDP-D-glucose 4,6-dehydratase	Cell wall/LPS/capsule

The genes are grouped in the table by genomic regions. ^a^As published in the annotation. ^b^The functional categories were assigned manually for this study. LPS, lipopolysaccharide.

**Table 3:** The genome of *Fracisella tularensis *supspecies *tularensis *Schu S4 encodes specific functions

	Gene accession number	Size of the predicted protein	G+C content (%)	Gene name^a^	Gene product description^a^	Functional category^b^
Genes inactivated or deleted in *novicida *and *holarctica *subspecies	FTT0097	181	31.1	-	Hypothetical protein FTT0097	Hypothetical
	FTT0432	469	30.3	speA	Putative arginine decarboxylase	Other metabolism
	FTT0435	286	34.9	-	Carbon-nitrogen hydrolase family protein	Other metabolism
	FTT0496	254	33.0	-	Hypothetical protein FTT0496	Hypothetical
	FTT0525	218	25.9	-	Hypothetical protein FTT0525	Hypothetical
	FTT0528	125	29.7	-	Hypothetical protein FTT0528	Hypothetical
	FTT0677c	258	27.2	-	Hypothetical protein FTT0677c	Hypothetical
	FTT0754c	111	24.0	-	Hypothetical membrane protein	Hypothetical
	FTT0939c	314	28.2	add	Adenosine deaminase	Nucleotides and nucleosides metabolism
	FTT1080c	292	24.8	-	Hypothetical membrane protein	Hypothetical
	FTT1122c	156	36.9	-	Hypothetical lipoprotein	Hypothetical
	FTT1598	944	34.3	-	Hypothetical membrane protein	Hypothetical
	FTT1666c	295	27.8	-	3-Hydroxyisobutyrate dehydrogenase	No functional role assigned
	FTT1667	78	26.5	-	Hypothetical protein FTT1667	Hypothetical
	FTT1766	218	33.5	-	O-methyltransferase	Cell wall/LPS/capsule
	FTT1781c	249	30.7	-	Hypothetical protein FTT1781c	Hypothetical
	FTT1784c	102	23.2	-	Hypothetical protein FTT1784c	Hypothetical
	FTT1787c	203	28.7	-	Transporter, LysE family	Transport
	FTT1789	264	29.1	-	Hypothetical protein FTT1789	Hypothetical

Sequences specific to the *tularensis *subspecies	FTT1066c	124	27.6	-	Hypothetical protein FTT1066c	Hypothetical
	FTT1068c	192	20.7	-	Hypothetical protein FTT1068c	Hypothetical
	FTT1069c	301	28.3	-	Hypothetical protein FTT1069c	Hypothetical
	FTT1071c	168	33.5	-	Hypothetical protein FTT1071c	Hypothetical
	FTT1072	209	31.6	-	Hypothetical protein FTT1072	Hypothetical
	FTT1073c	123	31.6	-	Hypothetical protein FTT1073c	Hypothetical
	FTT1308c	202	29.1	-	Hypothetical protein FTT1308c	Hypothetical
	FTT1580c	176	26.4	-	Hypothetical protein FTT1580c	Hypothetical
	FTT1791	120	30.1	-	Hypothetical protein FTT1791	Hypothetical

^a^As published in the annotation of the genome of Schu S4. ^b^The functional categories were assigned manually for this study. LPS, lipopolysaccharide.

#### Human pathogenic strains have undergone substantial loss of function, but not the non-pathogenic strain

Fourteen pseudogenes have been identified in U112 (Additional data file 1). In contrast, the original annotation of Schu S4 listed 201 pseudogenes [25]. Using the genome of U112 as a reference, 53 additional pseudogenes were predicted in the genome of Schu S4 (Additional data file 1) following a procedure described in Materials and methods (see below), most of which were annotated as multiple open reading frames (ORFs) in the published genome. Because the strain LVS was artificially attenuated, it is expected to contain mutations that are not found in any other *holarctica *genome. Indeed, 11 pseudogene-causing mutations were found to be specific to the LVS genome [39]. We ignored these 11 pseudogenes for the following comparative analysis, because they do not represent a loss of function in the *holarctica *subspecies as a whole.

When compared with the genome of U112, analysis of the genome of LVS revealed 303 pseudogenes in addition to those contained in IS elements (Additional data file 1). OK The number of protein encoding genes in the genome of LVS and the subspecies *holarctica *in general may therefore be about 1,400. The higher mutation rate observed in *holarctica *genomes as compared with *tularensis *could explain the greater number of pseudogenes. In addition, at least eight genes present in *novicida *and *holarctica *were lost by the strain Schu S4, and ten that were present in *novicida *and *tularensis *were lost by LVS. A set of 160 genes were inactivated in both LVS and Schu S4. Taking into account gene deletion and inactivation, U112 encodes 164 functions that are no longer active in both *holarctica *and *tularensis *strains. Similarly, 18 functions are specific to the strain Schu S4 and potentially to the subspecies *tularensis *in general (Table 3).

### Genomic comparison between human pathogenic strains and a strain nonpathogenic to humans provides a coarse chronology of the evolutionary events that took place during the emergence of the former

#### A reduced set of genes was inactivated in the genome of the strain ancestral to human pathogenic strains

**Figure 2:**
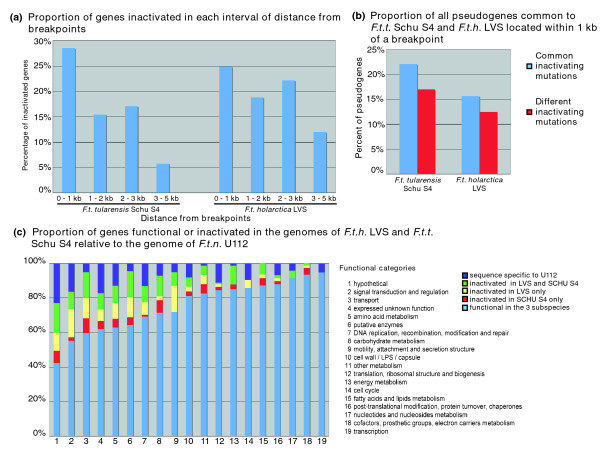
The distribution of pseudogenes is uneven in the genome and across functional categories. **(a) **Pseudogenes are more likely to be found near genomic breakpoints than in the rest of the genome. B. Genes inactivated both in Schu S4 and live vaccine strain (LVS) and sharing the same inactivating mutation are more likely to be near a genomic breakpoint than those not sharing the same inactivating mutation. **(c) **Missing and inactivated genes in the genomes of *Francisella tularensis *subspecies *tularensis *(*F.t.t*.) Schu S4 and *Francisella tularensis *subspecies *holarctica *(*F.t.h*.) LVS are not evenly distributed across functional categories. *F.t.n*., *Francisella tularensis *subspecies *novicida*; kb, kilobases; LPS, lipopolysaccharide.

A total of 160 genes are inactivated in the genomes of both subspecies *holarctica *and *tularensis*. Upon alignment of their sequences, 53% of pseudogenes common to LVS and Schu S4 exhibit at least one common mutation that may have led to their inactivation, whereas 32% of the pseudogenes common to both subspecies share no common variations. The sequence of the remaining 15% is too divergent to determine a potential common inactivating mutation (Additional data file 1). This indicates that at least 53% have arisen in the genome of the human pathogenic ancestor. These 82 pseudogenes bearing common mutations are more likely to be located directly at breakpoints than the pseudogenes not sharing any common mutation (Figure 2b). In addition, the IS insertion is the only inactivating common mutation found in 19 out the 82 pseudogenes from the ancestral strain. This suggests that IS insertions or subsequent sequence rearrangements contributed to at least 22% of the earliest gene inactivations that took place in the emerging human pathogenic strain.

#### Contribution of IS elements and other early mutations to genome reduction through initiation of genetic drift

When directly compared with the genome of U112, most pseudogenes in the genomes of Schu S4 and LVS appear to result from small indels (1 or 2 bp) or nonsense mutations. In *tularensis *and *holarctica *genomes, genes within 1 kb from a genomic breakpoint are twice as likely to be inactivated as were genes in other genomic locations (Figure 2a). The proportion of genes that are within 1 kb from a genomic breakpoint and are inactivated is 28.5% in the genome of Schu S4 (57 out of 200), whereas the global proportion of inactivated genes is 12.6%. Similarly, 24.9% of genes within 1 kb from genomic breakpoints are inactivated in the genome of LVS, whereas the global proportion of inactivated genes is 16.3%. Figure 2a shows that, to a lesser extent, the genes within 3 kb from a breakpoint are also more likely to be inactivated than are the genes in the rest of the genome. In Schu S4, 15.4% of genes between 1 and 2 kb from a breakpoint are inactivated and 17.1% are between 2 and 3 kb. Similarly in LVS 18.8% of the genes between 1 and 2 kb from a breakpoint and 22.1% between 2 and 3 kb are inactivated. It is unlikely that genomic rearrangements could directly have caused mutations as far as 3 kb from the breakpoints. It is more likely that the rearrangements disrupted the transcriptional unit to which these genes belong. If these genes are no longer transcribed, then their sequences are no longer subjected to selection and evolve by neutral genetic drift, eventually causing the disruption of the ORF through mutation.

In agreement with this conjecture, predicted operons located at breakpoints are more likely to contain more than one pseudogene, in Schu S4 by 4-fold and in LVS by 1.4-fold. An additional argument in favor of the inactivation of some genes by genetic drift is the uneven distribution of pseudogenes across functional categories (Figure 2c). Pseudogenes and absent genes of the *holarctica *and *tularensis *genomes have been assigned to functional categories based on the annotation of their functional counterpart in the genome of U112. For example, 41.2% of the genes predicted to be involved in amino acid biosynthesis in the genome of *novicida *are inactivated in the genome of one or both of the other subspecies. Similarly, 43.1% of the genes predicted to encode transporters are inactivated in the genomes of *holarctica *and *tularensis*. Remarkably, the distribution in functional categories is the same for genes inactivated in one genome and those inactivated in both. Likewise, it was previously observed in the genomes of *Salmonella typhi *and *S paratyphi *that the pseudogenes were different but appeared to belong to the same pathways and operons [11]. The over-representation of pseudogenes in certain functional categories suggests a loss of function associated with specific pathways, resulting in the decay of multiple genes in these categories [40]. Following the disruption of a biologic process by the inactivation of one gene, other genes involved in this process are no longer subjected to selective pressure.

#### Inactivation of the leucine and valine biosynthesis pathway illustrates the proposed evolutionary scenario

This example illustrates the proposed model of evolution of *Francisella *human pathogenic strains: initial inactivation of a gene in the ancestor of the subspecies *tularensis *and *holarctica *(potentially pathoadaptive) and further gene inactivation in regions no longer subjected to selective pressure before and after subspeciation.

**Figure 3:**
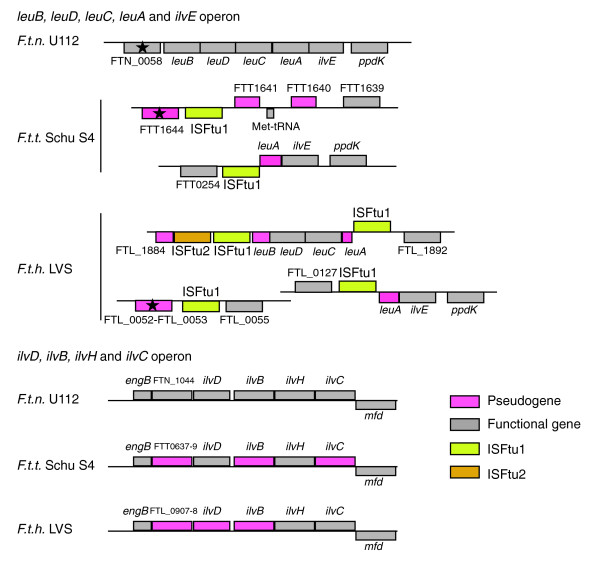
Inactivating mutations in two operons illustrate the ongoing process of gene decay. The *leu *operon and the *ilv *operon, which work in concert, accumulated inactivating mutations in the genome of *Francisella tularensis *subspecies *tularensis *(*F.t.t*.) Schu S4 and *F tularensis *subspecies *holarctica *(*F.t.h*.) live vaccine strain (LVS). The ISFtu1 element that disrupted *leuA *and the ISFtu1 integrated upstream of *leuB *share the same bordering sequences in both genomes. The inactivating mutation in *leuB *is the same in both genomes as well. Therefore, these events are believed to have taken place in the *leu *operon before divergence into two subspecies. The other mutations in the regions of the *leu *operon and the *ilv *operon are of different origins in the two genomes, indicating that these mutations took place after the subspeciation.

In the genome of U112, the genes involved in leucine and valine biosynthesis are organized in two operons: one contains *leuB*, *leuD*, *leuC*, *leuA*, and *ilvE*; and the other one contains *ilvD*, *ilvB*, *ilvH*, and *ilvC*. All genes are expressed in rich medium (Rohmer and coworkers, unpublished data). In the *tularensis *and *holarctica *strains the leucine, isoleucine, and valine biosynthesis pathway is inactivated. Based on the organization of the two regions depicted in Figure 3, we can infer events that took place in *leu *and *ilv *loci. Two ISFtu1 elements are associated with the *leu *operon in both human pathogenic strains and have the same bordering sequences: the same portions of *leuA *and the upstream sequence of *leuB*. Hence, the insertion of two ISFtu1 elements has taken place in the *leu *operon of the ancestor of the two strains and disrupted *leuA *and the upstream region of *leuB*. All sequences of the *leu *operon are still present in the genome of LVS, but they are scattered to three different locations, all associated with ISFtu1 elements. In the genome of Schu S4, *leuB*, *leuD*, and *leuC *have been deleted and one IS element sits in place of the deletion (Figure 3). It seems therefore that the two ISFtu1 elements inserted in the genome of the ancestor underwent different recombination events in each strain. The *ilv *operon contains distinct mutations in the genome of LVS and Schu S4; in LVS *ilvB *(FTL_0913-FTL_0914) and *ilvD *(FTL_0911-FTL_0912) are inactivated by a 100 bp deletion and a 350 bp deletion, respectively, whereas in Schu S4 *ilvC *(FTT0643) and *ilvB *(FTT0641) are inactivated because of a nonsense mutation and a single nucleotide deletion, respectively. The distinct origin of the inactivation of the *ilv *operon indicates that mutations took place after divergence as well.

### Predicted impact of the genetic differences on the pathogenicity of *F tularensis*

#### Potential virulence factors found in the U112 genome and common to all *F tularensis *strains

As described in the Introduction (above), virulence strategies overlap in the three subspecies. Here, we provide a list of virulence factors complementary to those previously predicted [16,25,41] using the U112 genome as a reference (Additional data file 2). A variety of protein features are potentially indicative of a role in virulence, such as the presence of a protein domain previously associated with a virulence function, the presence of a eukaryotic domain, or homology to eukaryotic proteins sufficiently high to suggest a role in the host cell [42-44]. A total of 129 proteins in U112 revealed one or more of these features. Interestingly, only 80 of them were present and functional in both of the other genomes (Additional data file 2). This suggests that many of these 129 proteins are not involved in virulence or are not essential for the virulence in humans. It is still conceivable that these proteins confer a capacity to infect hosts or to target functions that the subspecies *holarctica *and *tularensis *no longer utilize, or they may even be detrimental to the bacterium in the human host.

The ORF FTN_0921 in *novicida *U112 (FTT1043 in Schu S4) is homologous to a *Legionella *macrophage infectivity potentiator. FTN_1151 (FTT1170) contains Sel1 eukaryotic tetratrico peptide repeats and is homologous to EnhC and EnhA of *Coxiella burnetii*, which promote entry of *Coxiella *into host cells. These two proteins could contribute to entry of the bacteria into the macrophage. FNU1336 (FTT1332) may be a hemolysin. FTN_0403 (FTT0877c) is only homologous to eukaryotic proteins and, in particular, to a family of membrane-bound proteins with which it shares a pair of repeats, each spanning two transmembrane helices connected by a loop. The PQ motif found on loop 2 was shown to be critical for the localization of cystinosin to lysosomes [45]. FTN_0083 (FTT0243) may interact with the cytoskeleton of the host cell because it contains an α-tubulin suppressor or related RCC1 domain. FTN_0171 (FTT0195) has ankyrin repeats, sometimes present in bacterial virulence factors. Larsson and coworkers [25] pointed out that the genome of *Francisella tularensis *does not encode any of the secretion systems that are usually associated with pathogenicity (type III and type IV). A protein homologous to toxin secretion ABC transporters (FTN_1693) and HlyD-family secretion proteins (FTN_0029, FTN_0718, and FTN_1276) may play a role in the delivery of virulence factors. It has been shown that a secretion system similar to type II and type IV systems is responsible for the secretion of virulence factors in U112 [29]. TolC appears to play a role in virulence in U112 as well in *holarctica *strains [46]. Secretion through these systems first requires protein translocation through the bacterial inner membrane via an independent export system. A full and functional sec system was identified in the genome of U112 as well as in the genomes of Schu S4 and LVS. This suggests that some of the proteins that are exported outside the cell may contain a signal peptide, promoting their translocation across the inner membrane via the sec system. Hence, we suggest that there may be proteins that interact with host factors that are yet to be identified among the set of proteins with a predicted signal sequence.

#### Functions specific to the human pathogenic subspecies *holarctica *and *tularensis*

We consider functions to be specific to the human pathogenic subspecies if either their DNA sequence is solely found in these strains, or their counterparts in the nonpathogenic *novicida *are inactivated. We have found 41 genes whose DNA sequence is specific to *holarctica *and *tularensis *and five genes common to these subspecies that are pseudogenes in U112. In addition, there are 20 duplicated genes in Schu S4 and 34 in LVS. Included in this set is the duplicated pathogenicity island, of which there is only one copy in U112 [26]. The duplication of the *Francisella *pathogenicity island may provide a higher level of expression of the virulence genes it carries, as it is the case for the Shiga toxin genes in *Shigella dysenteriae *1 [47]. Potentially, greater expression of these pathogenicity genes could play a role in virulence in humans.

Among the 41 genes found solely in the genome of the *holarctica *and *tularensis *subspecies, 24 have no predicted function (Table 2). Some of the 41 genes could be linked to the pathogenicity of the human pathogenic strains. Six genes involved in the biosynthesis of the O-antigen of lipopolysaccharide in type A and type B strains have no counterparts in U112. The U112 subspecies carries a different set of genes for this function. This could explain the difference noted in the structure of the O-antigen of U112 as compared with those of *tularensis *strains [48]. The difference in the O-antigen part of the lipopolysaccharide structure could contribute to the difference in host range observed between the three subspecies. In addition to sequence-specific genes, five U112 pseudogenes are functional in both *holarctica *and *tularensis*. It may be that inactivation of these genes impairs the virulence of the strain U112 in humans, but the functions they encode do not suggest this possibility. Two of these genes encode nicotinamide ribonucleoside (NR) uptake permease family proteins (FTT0707 and FTT1090), but four other genes found in the U112 genome encode proteins of this family and some of their counterparts have become pseudogenes in the genome of *holarctica *and *tularensis *strains. Hence, these genes may have been inactivated because of functional redundancy. FTT0666c (homologous to some methylpurine-DNA glycosylases), inactivated in U112, may be involved in DNA repair following DNA damage induced by stress. FTT1076 (*hipA*), a protein that potentially is involved in persistence after exposure to antimicrobial products or other stressful conditions [49], is also inactivated in U112. It is therefore possible that U112 may be less resistant to human responses than the *holarctica *and *tularensis *strains. Finally, FTT1450c, *wbtM *on the O-antigen gene cluster, encodes a dTDP-D-glucose 4,6-dehydratase. Because some components of lipopolysaccharide are missing in U112, it is possible that FTT1450c in U112 has degenerated over time because of lack of selection. It would be interesting to examine the state of these five genes in the *novicida *strains isolated in humans [20,21,50].

#### Some of the functions specific to F tularensis subspecies *tularensis *Schu S4 may promote the high virulence of type A strains

Comparison between the three genomes reveals regions encoding nine proteins specific to Schu S4 and potentially to the subspecies *tularensis*. The RD8 11.1 kb specific region [37] carries six functional genes and two pseudogenes (FTT1066 to FTT1073). Three genes in this region suggest that it could be a phage remnant: a type III restriction-modification system restriction enzyme that is apparently nonfunctional (FTT1067); a DNA helicase, which is also nonfunctional (FTT1070); and a predicted antirestriction protein (FTT1071). The five other proteins have no predicted function. This region is bordered on each side by ISFtu1 elements. Because it is specific to all type A strains and exhibits properties of genomic islands (low G+C content and proteins related to mobile elements), the region may be a pathogenicity island that contributes to the virulence of *tularensis*. FTT1580c, a hypothetical protein, was detected in the region of difference RD1 [37] as specific to the subspecies *tularensis*. Two hypothetical proteins, namely FTT1308c and FTT1791, were also determined to be specific to Schu S4 in the three-way comparison. They were not detected in the regions of difference obtained by Broekhuijsen and coworkers [37] and Svensson and colleagues [38], and so it is possible that these genes are not specific to *tularensis *strains or are not present in all *tularensis *strains. Alternatively, the differences are not detectable with the techniques used by the authors.

In addition to the sequence-specific functions, some functions (encoded by 20 genes) are specific to Schu S4 because they are pseudogenes or absent in the genomes of U112 and LVS. Table 3 lists these 20 genes. A predicted O-methyltransferase (FTT1766) is only functional in Schu S4, and could influence the composition of the bacterial surface. FTT0939, an adenosine deaminase, is only functional in type A strains. This enzyme is predicted to be involved in purine salvage. This could be important to consider for vaccine design, because inactivation of the purine biosynthesis pathway of a type A strain may not result in the significant reduction of fitness that has been observedin type B [51] and *novicida *strains (data not shown).

#### Loss of function specific to *holarctica *may be responsible for the lower level of virulence of these strains when compared with *tularensis *strains

Eight additional genes involved in regulation are inactivated in the genome of *holarctica *alone (Additional data file 1). Six of these genes belong to the LysR transcriptional regulator family. The regulators of the LysR family have diverse targets, including virulence genes and genes that are involved in response to a specific environment. The genome of *holarctica *strains also exhibits a higher number of pseudogenes in the functional category 'motility, attachment, and secretion structure'. Although three genes encoding potential pilins are inactivated in both subspecies, the *holarctica *genome underwent inactivation of four additional genes encoding pilins and two predicted to encode membrane fusion proteins. Attachment and motility are key aspects of pathogenicity, and inactivation of these genes may lower the efficiency of infection of humans by *holarctica *strains. In addition, six genes that are potentially involved in DNA repair are solely inactivated in *holarctica *(including one encoding a photolyase that repairs mismatched pyrimidine dimers, and one that encodes the protein mutT, which is involved in removing an oxidatively damaged form of guanine). This could explain the higher rate of mutation in *holarctica *strains than in *tularensis *strains, and may indirectly be responsible for the inactivation of genes that are important for the pathogenicity of *holarctica *strains.

#### Loss of function common to *tularensis *and *holarctica *provide clues to possible pathoadptation and to the properties of the environmental niches they occupy during their life cycle

Our data suggest that more than half of the pseudogenes in the human pathogenic strains appeared relatively late in their evolution, after the subspeciation. If pathoadaptive mutations occurred, then it is more likely that they took place before the divergence of the pathogenic strains, rather than twice, independently in each pathogenic subspecies. The 84 pseudogenes in the two human pathogenic strains that have arisen in the genome of their common ancestor are listed in Additional data file 1. Significantly, the gene *pepO *is part of these early mutants in the human pathogenic strains. This gene is active in U112, but a strain U112 in which *pepO *(FTN_1186) is inactivated spreads more to systemic sites [29]. Similarly, the system used to secrete pepO and other proteins [29] was also altered in the ancestor of the human pathogenic strains (FTN_0306 and FTN_0389). The distribution of the early pseudogenes across functional categories is similar to the distribution of the entire set of pseudogenes (data not shown). However, although eight independent pathways of amino acid biosynthesis are inactivated in one or both human pathogenic strains (24 genes), only one biosynthesis pathway is inactivated in the ancestral strain: the biosynthesis pathway for leucine, isoleucine, and valine. This suggests that the biosynthesis of most amino acids is not required in the current niche of *tularensis *and *holarctica *subspecies, but also that only leucine/isoleucine/valine biosynthesis may have played a role in preventing virulence in the human niche. Three transcriptional regulators are inactivated in both genomes: two regulators of the LysR family, and *kdpD *and *kdpE*, which form a two-component regulator. Numerous genes encoding transporters are also inactivated. Hence, it is apparent that the *tularensis *and *holarctica *subspecies have lost their ability to adapt to or exploit some conditions, and perhaps have undergone niche restriction.

## Conclusion

The three-way genomic comparison described in this study illustrates the value of comparing closely related genomes of a nonpathogenic strain and human pathogenic strains. It allowed us to perform a detailed analysis of the events that may have led to the emergence of *Francisella *human pathogenic strains. The emergence could have been initiated by the gain or loss of function (pathoadaptivity) that took place in a few bacteria, an event that enables them to colonize an environment *de novo*, or more successfully than before. This step constitutes a first evolutionary bottleneck because only a small number of bacteria undergo the genomic change, and any mutation that was carried in this restricted set of bacteria is conserved within the pathogenic population. Consequently, IS transpositions and nucleotide substitution may have caused gene decay as the result of genetic drift and evolutionary bottlenecks (such as small inocula during an infection). The features of the *holarctica *and *tularensis *genomes are consistent with those observed in other facultative or recent obligate intracellular highly pathogenic bacteria. Consequently, our analysis could contribute to deciphering the evolutionary processes that take place in other facultative or recent obligate intracellular, highly pathogenic bacteria.

## Materials and methods

### Genome sequencing and validation

Whole genome shotgun sequencing was used to sequence the *F tularensis *subspecies *novicida *U112 [52] genome, as per the standard protocols followed in the University of Washington Genome Center [53,54]. In all, 32,180 plasmid and 1,728 fosmid paired-end sequencing reads were attempted, which provided 10.3× sequence coverage for the U112 genome (average Q20 614 bases/read, failure rate 16.3%). The genome was assembled using Phred/Phrap software tools [55,56] and viewed in CONSED [57]. The assembly contained 213 contigs, with 98 contigs being more than 2 kb in size. Genome finishing was initially attempted by carrying out experiments designed by the Autofinish tool in CONSED [58]. Manual finishing by an expert finisher followed four reiterative rounds of Autofinish. The finished *F tularensis *subspecies *novicida *U112 genome assembly contained 29,180 sequencing reads. Experimentally derived fingerprints from fosmid clones were compared with the virtual sequence-derived fragments from the finished genome using the SeqTile software developed in-house (Gillett, unpublished data). Correspondence between the experimentally and sequence derived fingerprints was observed, validating the final *F tularensis *subspecies *novicida *U112 genome assembly. The replication origin was determined using the software Oriloc [59].

### Genome-wide comparisons

The genome sequences of *F tularensis *subspecies *holarctica *strain LVS and *F tularensis *subspecies *tularensis *Schu S4 used were those of the published annotation (NC_007880 and NC_006570, respectively). Genomic sequence comparisons were performed with the program Nucmer from the package MUMmer [60] using a minimum cluster length of 650 bp. The software show-coords of the same package was then used to infer the degree of similarity and to map the genomic fragments of the query genome onto the reference genome. Additional curation of the output of show-coords was performed using custom Perl scripts. Fragments inferred to be strain specific were searched against the genomes of other strains using the algorithm megablast [61] to confirm their specificity.

### Identification of genes in *Francisella *genomes

Protein coding sequences in the genome of *F tularensis *subspecies *novicida *strain U112 were predicted using Glimmer 2.13 [62] and manually curated. The protein coding regions for *F tularensis *subspecies *holarctica *strain LVS and *F tularensis *subspecies *tularensis *Schu S4 were those of the published annotation (NC_007880 and NC_006570, respectively).

### Identification and comparison of the three *Francisella *genomes

We initially used the protein sequences to determine orthologous genes. Orthologous proteins in the three strains were first determined by reciprocal best hit (RBH) using the blastp algorithm [63,64]. When no orthologous gene was found in one genome, the blastn algorithm was used to search for a matching sequence in the genome in which it was missing, and - when present - the sequence was associated with the sequences of the orthologs in the other genomes. When the orthologous protein sequences differed in length by more than 30% (a threshold more conservative than the standard [20%] determined by Lerat and coworkers [65,66]), the gene encoding the shortest protein was designated a pseudogene, which represented about 73% of all pseudogenes in the genome of Schu S4. When the size differed by 10% to 30%, the protein alignments were examined and the status of the gene (functional or pseudogene) was assigned manually. Usually, these cases matched pseudogenes with a frameshift leading to a protein of similar size or a mutation close to the 5' extremity (such as an IS element insertion), where the ORF predictor would predict an ORF beginning at the next available start codon.

### Genome annotation

Gene descriptions and functional categories were manually determined based on homologies to domains found in the PFAM database [67], the Prosite database [68], and the cdd database [69]; homologies to proteins of the nr database and the TCDB database [70]; as well as by complementary approaches such as the Gotcha method [71] and the Pathway tools software [72]. A distinction was made between genes encoding hypothetical proteins, for which no significant homology could be detected in any database except for nr, and genes encoding proteins of unknown function, for which no significant homology could be detected in any database except for nr, but were shown to be expressed by U112 in rich medium (data not shown). Transcriptional units were predicted using the operon finding software (ofs) version 1.2 [73] and selecting all predictions with a final probability of 0.46 or greater. The size of the operons varied from two to 29 genes (encoding ribosomal proteins). tRNAs were determined with tRNAscan-SE [74]. rRNA operons were determined by searching the genome for conserved rRNA sequences using the blastn algorithm [63]. The cellular location of encoded proteins was predicted with PSORTB [75]. The presence of a potential signal peptide necessary for secretion by the sec system was predicted with signalP [76]. IS elements were identified using the megablast algorithm [61] with the sequences from the ISfinder database that were kindly provided by the database curators [77]. Proteins with domains associated with transposase activity were all examined manually. The annotation was added into Genbank (Refseq: NC_008601).

## Additional data files

The following additional data are available with the online version of this paper. Additional data file 1 lists the 1,745 genes (functional or inactivated) that were identified in *F tularensis *subspecies *novicida U112*; their orthologous counterparts in the genome of *F tularensis *subspecies *tularensis *Schu S4 and *F tularensis *subspecies *holarctica *LVS are listed when available. Additional data file 2 catalogs the 80 candidate virulence genes of *F tularensis *subspecies *novicida *U112 that are also present in *holarctica *and *tularensis *genomes. Additional data file 3 lists the duplicated genes (100% identity) in the genomes of *F tularensis *subspecies *tularensis *Schu S4 and *F tularensis *subspecies *holarctica *LVS, and their counterpart in *F tularensis *subspecies *novicida *U112.

## Supplementary Material

Additional data file 1 A total of 1,745 genes (functional or inactivated) were identified in *Francisella tularensis *subspecies *novicida *U112; its orthologous counterpart in the genome of *Francisella tularensis *subspecies *tularensis *Schu S4 and *Francisella tularensis *subspecies *holarctica *LVS is listed when available. Click here for file

Additional data file 2 Eighty candidate virulence genes of *Francisella tularensis *subspecies *novicida *U112 are also present in *holarctica *and *tularensis *genomes. Click here for file

Additional data file 3 Provided is a list of the duplicated genes (100% identity) in the genomes of *Francisella tularensis *subspecies *tularensis *Schu S4 and *Francisella tularensis *subspecies *holarctica *LVS, and their counterpart in *Francisella tularensis *subspecies *novicida *U112. Click here for file

## References

[B1] Groisman EA, Ochman H (1996). Pathogenicity islands: bacterial evolution in quantum leaps.. Cell.

[B2] Hacker J, Kaper JB (2000). Pathogenicity islands and the evolution of microbes.. Annu Rev Microbiol.

[B3] Jores J, Rumer L, Wieler LH (2004). Impact of the locus of enterocyte effacement pathogenicity island on the evolution of pathogenic *Escherichia coli*.. Int J Med Microbiol.

[B4] Parkhill J, Sebaihia M, Preston A, Murphy LD, Thomson N, Harris DE, Holden MT, Churcher CM, Bentley SD, Mungall KL (2003). Comparative analysis of the genome sequences of *Bordetella pertussis*, *Bordetella parapertussis *and *Bordetella bronchiseptica*.. Nat Genet.

[B5] Moore RA, Reckseidler-Zenteno S, Kim H, Nierman W, Yu Y, Tuanyok A, Warawa J, DeShazer D, Woods DE (2004). Contribution of gene loss to the pathogenic evolution of *Burkholderia pseudomallei *and *Burkholderia mallei*.. Infect Immun.

[B6] Maurelli AT, Fernandez RE, Bloch CA, Rode CK, Fasano A (1998). 'Black holes' and bacterial pathogenicity: a large genomic deletion that enhances the virulence of *Shigella *spp. and enteroinvasive *Escherichia coli*.. Proc Natl Acad Sci USA.

[B7] Foreman-Wykert AK, Miller JF (2003). Hypervirulence and pathogen fitness.. Trends Microbiol.

[B8] Ochman H, Davalos LM (2006). The nature and dynamics of bacterial genomes.. Science.

[B9] Moran NA, Plague GR (2004). Genomic changes following host restriction in bacteria.. Curr Opin Genet Dev.

[B10] Mira A, Pushker R, Rodriguez-Valera F (2006). The Neolithic revolution of bacterial genomes.. Trends Microbiol.

[B11] McClelland M, Sanderson KE, Clifton SW, Latreille P, Porwollik S, Sabo A, Meyer R, Bieri T, Ozersky P, McLellan M (2004). Comparison of genome degradation in *paratyphi A *and *typhi*, human-restricted serovars of *Salmonella enterica *that cause typhoid.. Nat Genet.

[B12] Wei J, Goldberg MB, Burland V, Venkatesan MM, Deng W, Fournier G, Mayhew GF, Plunkett G, Rose DJ, Darling A (2003). Complete genome sequence and comparative genomics of *Shigella flexneri *serotype 2a strain 2457T.. Infect Immun.

[B13] Chain PS, Hu P, Malfatti SA, Radnedge L, Larimer F, Vergez LM, Worsham P, Chu MC, Andersen GL (2006). Complete genome sequence of *Yersinia pestis *strains Antiqua and Nepal516: evidence of gene reduction in an emerging pathogen.. J Bacteriol.

[B14] Cole ST, Eiglmeier K, Parkhill J, James KD, Thomson NR, Wheeler PR, Honore N, Garnier T, Churcher C, Harris D (2001). Massive gene decay in the leprosy bacillus.. Nature.

[B15] Yang F, Yang J, Zhang X, Chen L, Jiang Y, Yan Y, Tang X, Wang J, Xiong Z, Dong J (2005). Genome dynamics and diversity of *Shigella *species, the etiologic agents of bacillary dysentery.. Nucleic Acids Res.

[B16] Petrosino JF, Xiang Q, Karpathy SE, Jiang H, Yerrapragada S, Liu Y, Gioia J, Hemphill L, Gonzalez A, Raghavan TM (2006). Chromosome rearrangement and diversification of *Francisella tularensis *revealed by the type B (OSU18) genome sequence.. J Bacteriol.

[B17] Forsman M, Sandström G, Jaurin B (1990). Identification of *Francisella *species and discrimination of type A and type B strains of *F. tularensis *by 16S rRNA analysis.. Appl Environ Microbiol.

[B18] Johansson A, Farlow J, Larsson P, Dukerich M, Chambers E, Byström M, Fox J, Chu M, Forsman M, Sjöstedt A (2004). Worldwide genetic relationships among Francisella tularensis isolates determined by multiple-locus variable-number tandem repeat analysis.. J Bacteriol.

[B19] Santic M, Molmeret M, Abu Kwaik Y (2005). Modulation of biogenesis of the *Francisella tularensis *subsp. *novicida*-containing phagosome in quiescent human macrophages and its maturation into a phagolysosome upon activation by IFN-gamma.. Cell Microbiol.

[B20] Hollis DG, Weaver RE, Steigerwalt AG, Wenger JD, Moss CW, Brenner DJ (1989). *Francisella philomiragia *comb. nov. (formerly *Yersinia philomiragia*) and *Francisella tularensis *biogroup *novicida *(formerly *Francisella novicida*) associated with human disease.. J Clin Microbiol.

[B21] Clarridge JE, Raich TJ, Sjosted A, Sandstrom G, Darouiche RO, Shawar RM, Georghiou PR, Osting C, Vo L (1996). Characterization of two unusual clinically significant *Francisella *strains.. J Clin Microbiol.

[B22] Santic M, Molmeret M, Klose KE, Abu Kwaik Y (2006). *Francisella tularensis *travels a novel, twisted road within macrophages.. Trends Microbiol.

[B23] Sjöstedt A (2006). Intracellular survival mechanisms of *Francisella tularensis*, a stealth pathogen.. Microbes Infect.

[B24] Dempsey MP, Nietfeldt J, Ravel J, Hinrichs S, Crawford R, Benson AK (2006). Paired-end sequence mapping detects extensive genomic rearrangement and translocation during divergence of *Francisella tularensis *subsp. *tularensis *and *Francisella tularensis *subsp. *holarctica *populations.. J Bacteriol.

[B25] Larsson P, Oyston PC, Chain P, Chu MC, Duffield M, Fuxelius HH, Garcia E, Halltorp G, Johansson D, Isherwood KE (2005). The complete genome sequence of *Francisella tularensis*, the causative agent of tularemia.. Nat Genet.

[B26] Nano FE, Zhang N, Cowley SC, Klose KE, Cheung KK, Roberts MJ, Ludu JS, Letendre GW, Meierovics AI, Stephens G (2004). A *Francisella tularensis *pathogenicity island required for intramacrophage growth.. J Bacteriol.

[B27] Lai XH, Golovliov I, Sjöstedt A (2004). Expression of IglC is necessary for intracellular growth and induction of apoptosis in murine macrophages by *Francisella tularensis*.. Microb Pathog.

[B28] Lindgren H, Golovliov I, Baranov V, Ernst RK, Telepnev M, Sjöstedt A (2004). Factors affecting the escape of *Francisella tularensis *from the phagolysosome.. J Med Microbiol.

[B29] Hager AJ, Bolton DL, Pelletier MR, Brittnacher MJ, Gallagher LA, Kaul R, Skerrett SJ, Miller SI, Guina T (2006). Type IV pili-mediated secretion modulates *Francisella *virulence.. Mol Microbiol.

[B30] Lauriano CM, Barker JR, Yoon SS, Nano FE, Arulanandam BP, Hassett DJ, Klose KE (2004). MglA regulates transcription of virulence factors necessary for *Francisella tularensis *intraamoebae and intramacrophage survival.. Proc Natl Acad Sci USA.

[B31] Konstantinidis KT, Tiedje JM (2005). Genomic insights that advance the species definition for prokaryotes.. Proc Natl Acad Sci USA.

[B32] Nubel U, Reissbrodt R, Weller A, Grunow R, Porsch-Ozcurumez M, Tomaso H, Hofer E, Splettstoesser W, Finke EJ, Tschape H (2006). Population structure of *Francisella tularensis*.. J Bacteriol.

[B33] Garcia Del Blanco N, Dobson ME, Vela AI, De La Puente VA, Gutierrez CB, Hadfield TL, Kuhnert P, Frey J, Dominguez L, Rodriguez Ferri EF (2002). Genotyping of *Francisella tularensis *strains by pulsed-field gel electrophoresis, amplified fragment length polymorphism fingerprinting, and 16S rRNA gene sequencing.. J Clin Microbiol.

[B34] Thomas R, Johansson A, Neeson B, Isherwood K, Sjöstedt A, Ellis J, Titball RW (2003). Discrimination of human pathogenic subspecies of *Francisella tularensis *by using restriction fragment length polymorphism.. J Clin Microbiol.

[B35] Farlow J, Smith KL, Wong J, Abrams M, Lytle M, Keim P (2001). *Francisella tularensis *strain typing using multiple-locus, variable-number tandem repeat analysis.. J Clin Microbiol.

[B36] Gill SR, Fouts DE, Archer GL, Mongodin EF, Deboy RT, Ravel J, Paulsen IT, Kolonay JF, Brinkac L, Beanan M (2005). Insights on evolution of virulence and resistance from the complete genome analysis of an early methicillin-resistant *Staphylococcus aureus *strain and a biofilm-producing methicillin-resistant *Staphylococcus epidermidis *strain.. J Bacteriol.

[B37] Broekhuijsen M, Larsson P, Johansson A, Byström M, Eriksson U, Larsson E, Prior RG, Sjöstedt A, Titball RW, Forsman M (2003). Genome-wide DNA microarray analysis of *Francisella tularensis *strains demonstrates extensive genetic conservation within the species but identifies regions that are unique to the highly virulent *F. tularensis *subsp. *tularensis*.. J Clin Microbiol.

[B38] Svensson K, Larsson P, Johansson D, Bystrom M, Forsman M, Johansson A (2005). Evolution of subspecies of *Francisella tularensis*.. J Bacteriol.

[B39] Rohmer L, Brittnacher M, Svensson K, Buckley D, Haugen E, Zhou Y, Chang J, Levy R, Hayden H, Forsman M (2006). Potential source of *Francisella tularensis *live vaccine strain attenuation determined by genome comparison.. Infect Immun.

[B40] Dagan T, Blekhman R, Graur D (2006). The 'domino theory' of gene death: gradual and mass gene extinction events in three lineages of obligate symbiotic bacterial pathogens.. Mol Biol Evol.

[B41] Brotcke A, Weiss DS, Kim CC, Chain P, Malfatti S, Garcia E, Monack DM (2006). Identification of MglA-regulated genes reveals novel virulence factors in *Francisella tularensis*.. Infect Immun.

[B42] Szurek B, Marois E, Bonas U, Van den Ackerveken G (2001). Eukaryotic features of the *Xanthomonas *type III effector AvrBs3: protein domains involved in transcriptional activation and the interaction with nuclear import receptors from pepper.. Plant J.

[B43] Hornef MW, Wick MJ, Rhen M, Normark S (2002). Bacterial strategies for overcoming host innate and adaptive immune responses.. Nat Immunol.

[B44] Knodler LA, Celli J, Finlay BB (2001). Pathogenic trickery: deception of host cell processes.. Nat Rev Mol Cell Biol.

[B45] Cherqui S, Kalatzis V, Trugnan G, Antignac C (2001). The targeting of cystinosin to the lysosomal membrane requires a tyrosine-based signal and a novel sorting motif.. J Biol Chem.

[B46] Gil H, Platz GJ, Forestal CA, Monfett M, Bakshi CS, Sellati TJ, Furie MB, Benach JL, Thanassi DG (2006). Deletion of TolC orthologs in *Francisella tularensis *identifies roles in multidrug resistance and virulence.. Proc Natl Acad Sci USA.

[B47] McDonough MA, Butterton JR (1999). Spontaneous tandem amplification and deletion of the shiga toxin operon in *Shigella dysenteriae *1.. Mol Microbiol.

[B48] Vinogradov E, Conlan WJ, Gunn JS, Perry MB (2004). Characterization of the lipopolysaccharide O-antigen of *Francisella novicida *(U112).. Carbohydr Res.

[B49] Gerdes K, Christensen SK, Lobner-Olesen A (2005). Prokaryotic toxin-antitoxin stress response loci.. Nat Rev Microbiol.

[B50] Whipp MJ, Davis JM, Lum G, de Boer J, Zhou Y, Bearden SW, Petersen JM, Chu MC, Hogg G (2003). Characterization of a *novicida*-like subspecies of *Francisella tularensis *isolated in Australia.. J Med Microbiol.

[B51] Pechous R, Celli J, Penoske R, Hayes SF, Frank DW, Zahrt TC (2006). Construction and characterization of an attenuated purine auxotroph in a *Francisella tularensis *live vaccine strain.. Infect Immun.

[B52] Larson CL, Wicht W, Jellison WL (1955). A new organism resembling *F. tularensis *isolated from water.. Public Health Rep.

[B53] Wood DW, Setubal JC, Kaul R, Monks DE, Kitajima JP, Okura VK, Zhou Y, Chen L, Wood GE, Almeida NF (2001). The genome of the natural genetic engineer *Agrobacterium tumefaciens *C58.. Science.

[B54] Hendrickson EL, Kaul R, Zhou Y, Bovee D, Chapman P, Chung J, Conway de Macario E, Dodsworth JA, Gillett W, Graham DE (2004). Complete genome sequence of the genetically tractable hydrogenotrophic methanogen *Methanococcus maripaludis*.. J Bacteriol.

[B55] Ewing B, Green P (1998). Base-calling of automated sequencer traces using phred. II. Error probabilities.. Genome Res.

[B56] Ewing B, Hillier L, Wendl MC, Green P (1998). Base-calling of automated sequencer traces using phred. I. Accuracy assessment.. Genome Res.

[B57] Gordon D, Abajian C, Green P (1998). Consed: a graphical tool for sequence finishing.. Genome Res.

[B58] Gordon D, Desmarais C, Green P (2001). Automated finishing with autofinish.. Genome Res.

[B59] Frank AC, Lobry JR (2000). Oriloc: prediction of replication boundaries in unannotated bacterial chromosomes.. Bioinformatics.

[B60] Kurtz S, Phillippy A, Delcher AL, Smoot M, Shumway M, Antonescu C, Salzberg SL (2004). Versatile and open software for comparing large genomes.. Genome Biol.

[B61] Zhang Z, Schwartz S, Wagner L, Miller W (2000). A greedy algorithm for aligning DNA sequences.. J Comput Biol.

[B62] Delcher AL, Harmon D, Kasif S, White O, Salzberg SL (1999). Improved microbial gene identification with GLIMMER.. Nucleic Acids Res.

[B63] Altschul SF, Madden TL, Schaffer AA, Zhang J, Zhang Z, Miller W, Lipman DJ (1997). Gapped BLAST and PSI-BLAST: a new generation of protein database search programs.. Nucleic Acids Res.

[B64] Rivera MC, Jain R, Moore JE, Lake JA (1998). Genomic evidence for two functionally distinct gene classes.. Proc Natl Acad Sci USA.

[B65] Lerat E, Ochman H (2005). Recognizing the pseudogenes in bacterial genomes.. Nucleic Acids Res.

[B66] Lerat E, Ochman H (2004). Psi-Phi: exploring the outer limits of bacterial pseudogenes.. Genome Res.

[B67] PFAM database. http://pfam.wustl.edu/.

[B68] Prosite database. http://www.expasy.org/prosite/.

[B69] cdd database. ftp://ftp.ncbi.nih.gov/pub/mmdb/cdd/.

[B70] TCDB database. http://www.tcdb.org/.

[B71] Martin DM, Berriman M, Barton GJ (2004). GOtcha: a new method for prediction of protein function assessed by the annotation of seven genomes.. BMC Bioinformatics.

[B72] Karp PD, Paley S, Romero P (2002). The Pathway Tools software.. Bioinformatics.

[B73] Westover BP, Buhler JD, Sonnenburg JL, Gordon JI (2005). Operon prediction without a training set.. Bioinformatics.

[B74] Lowe TM, Eddy SR (1997). tRNAscan-SE: a program for improved detection of transfer RNA genes in genomic sequence.. Nucleic Acids Res.

[B75] Gardy JL, Laird MR, Chen F, Rey S, Walsh CJ, Ester M, Brinkman FS (2005). PSORTb v.2.0: expanded prediction of bacterial protein subcellular localization and insights gained from comparative proteome analysis.. Bioinformatics.

[B76] Bendtsen JD, Nielsen H, von Heijne G, Brunak S (2004). Improved prediction of signal peptides: SignalP 3.0.. J Mol Biol.

[B77] Siguier P, Perochon J, Lestrade L, Mahillon J, Chandler M (2006). ISfinder: the reference centre for bacterial insertion sequences.. Nucleic Acids Res.

