# Supplementary material for: Comparison of Francisella tularensis genomes reveals evolutionary events associated with the emergence of human pathogenic strains
Source: Genome Biol. 2007 Jun 5;8(6):R102. doi: 10.1186/gb-2007-8-6-r102 (PMC2394750; doi:10.1186/gb-2007-8-6-r102)
Supplement: Additional data file 1 — A total of 1,745 genes (functional or inactivated) were identified in Francisella tularensis subspecies novicida U112; its orthologous counterpart in the genome of Francisella tularensis subspecies tularensis Schu S4 and Francisella tularensis subspecies holarctica LVS is listed when available. [file gb-2007-8-6-r102-S1.pdf]

Supplemental Table 1: List of 1745 genes (functional or inactivated) identified in *F.t. novicida U112, and their* orthologous counterparts in the genome of *F.t. tularensis* Schu S4 and *F.t. holarctica* LVS when availabl

Locus tags for genes in U112[1]	Locus tags of orthologs in Schu S4[1]	Locus tags for orthologs in LVS[1]	gene location in U112 genome (5'-3')		Predicted protein size (aa)	Gene name	Gene description	Functional category and subcategory	Predicted protein localization	Inactivation in ancestor of Schu S4 and LVS
FTN 0001	FTT0001	FTL 0001	145	1617	491	dnaA	chromosomal replication initiator protein	DNA replication, recombination, modification and repair - restriction/modification	cytoplasm	
FTN 0002	FTT0002	FTL 0002	1657	2757	367	dnaN	DNA polymerase III, beta subunit	DNA replication, recombination, modification and repair - restriction/modification	cytoplasm	
FTN 0003	(FTT0003c)	(FTL 0003)	4044	2794	417	-	metabolite:H+ symporter (MHS) family protein	transport	cytoplasmic membrane	yes
FTN 0004	Schunoseq	LVSnoseq	4244	5764	507	-	aspartate/glutamate transporter	transport - amino-acid	cytoplasmic membrane	
FTN 0005	Schunoseq	LVSnoseq	5832	6800	323	corA	divalent inorganic cation transporter	transport	unknown	
FTN 0006	Schunoseq	LVSnoseq	7283	6819	155	-	hypothetical protein	hypothetical - novel	unknown	
FTN 0007	Schunoseq	LVSnoseq	7678	7364	105	-	hypothetical protein	hypothetical - novel	unknown	
FTN 0008	Schunoseq	LVSnoseq	8640	7771	290	-	10 TMS drug/metabolite exporter protein	transport - drugs / antibacterial compounds	unknown	
FTN 0009	(1738516-1738597)	(22290-22472)	8784	8963	60	-	hypothetical protein	hypothetical - novel	unknown	yes
FTN 0010	Schunoseq	LVSnoseq	9806	9375	144	-	phage terminase, small subunit	mobile and extrachromosomal element functions - phage or plasmid related proteins	unknown	
FTN 0011	Schunoseq	LVSnoseq	10220	10044	59	-	hypothetical protein	hypothetical - novel	unknown	
FTN 0012	Schunoseq	LVSnoseq	10438	10220	73	-	hypothetical protein	hypothetical - novel	unknown	
FTN 0013	Schunoseq	LVSnoseq	11835	10939	299	-	hypothetical protein	hypothetical - novel	unknown	
FTN 0014	Schunoseq	LVSnoseq	12728	11835	298	-	conserved hypothetical protein	hypothetical - conserved	unknown	
FTN 0015	Schunoseq	LVSnoseq	12911	12735	59	-	regulatory protein, AlpA family	signal transduction and regulation	unknown	
FTN 0016	Schunoseq	LVSnoseq	13781	13110	224	-	hypothetical protein	hypothetical - novel	unknown	
FTN 0017	Schunoseq	LVSnoseq	15027	13789	413	-	phage integrase	mobile and extrachromosomal element functions - phage or plasmid related proteins	unknown	
FTN 0018	FTT1668	FTL 0024	16908	15688	407	sdaC	serine permease	transport - amino-acid	unknown	
FTN 0019	FTT1665	FTL 0028	17975	17052	308	pyrB	aspartate carbamoyltransferase	nucleotides and nucleosides metabolism	unknown	
FTN 0020	FTT1664	FTL 0029	21401	18120	1094	carB	carbamoyl-phosphate synthase large chain	nucleotides and nucleosides metabolism	unknown	
FTN 0021	FTT1663	FTL 0030	22572	21409	388	carA	carbamoyl-phosphate synthase small chain	nucleotides and nucleosides metabolism	unknown	
FTN 0022	(FTT1662c)	FTL 0031	22992	24044	351	-	histidine acid phosphatase	putative enzymes	unknown	
FTN 0023	FTT1661	FTL 0032	24748	24071	226	tmpT	thiopurine S-methyltransferase	putative enzymes	cytoplasm	
FTN 0024	FTT1660c	FTL 0033	24880	26223	448	pyrC	dihydroorotase	nucleotides and nucleosides metabolism	unknown	
FTN 0025	Schunoseq	LVSnoseq	26797	26213	195	-	hypothetical protein	hypothetical - novel	cytoplasm	
FTN 0026	FTT1658c	36948-37201	27076	27327	84	-	hypothetical protein	hypothetical - novel	unknown	
FTN 0027	FTT1656c	FTL 0036	28044	28592	183	-	conserved protein of unknown function	unknown function - conserved	unknown	
FTN 0028	FTT1655	FTL 0037	29763	28714	350	-	conserved hypothetical membrane protein	hypothetical - conserved	cytoplasmic membrane	
FTN 0029	FTT1654	FTL 0038	30715	29753	321	-	membrane fusion protein, HlyD family	transport	periplasm	
FTN 0030	FTT1653	FTL 0039	31209	30805	135	-	hypothetical membrane protein	hypothetical - novel	unknown	
FTN 0031	FTT1652c	FTL 0040	31335	32201	289	-	transcriptional regulator, LysR family	signal transduction and regulation	unknown	
FTN 0032	FTT1651	(FTL_0041-42747)	32831	32208	208	-	protein of unknown function	unknown function - novel	unknown	
FTN 0033	FTT1650c	FTL 0043	32974	33519	182	-	conserved protein of unknown function	unknown function - conserved	unknown	
FTN 0034	(FTT1649)	FTL 0044	36951	33580	1124	-	conserved protein of unknown function	unknown function - conserved	unknown	
FTN 0035	FTT1648c	FTL 0045	37218	37859	214	pyrF	orotidine-5'-phosphate decarboxylase	nucleotides and nucleosides metabolism	unknown	
FTN 0036	FTT1647c	FTL 0046	37864	38601	246	pyrD	dihydroorotate oxidase	nucleotides and nucleosides metabolism	unknown	
FTN 0037	Schunoseq	LVSnoseq	40347	40006	114	-	hypothetical protein	hypothetical - novel	unknown	
FTN 0038	Schunoseq	LVSnoseq	40650	40369	94	-	hypothetical protein	hypothetical - novel	unknown	
FTN 0039	Schunoseq	LVSnoseq	41078	40656	141	-	protein of unknown function	unknown function - novel	unknown	
FTN 0040	Schunoseq	LVSnoseq	44347	41096	1084	-	protein of unknown function	unknown function - conserved	unknown	
FTN 0041	Schunoseq	LVSnoseq	46582	44369	738	-	protein of unknown function	unknown function - novel	outer membrane	
FTN 0042	Schunoseq	LVSnoseq	46882	47436	185	-	conserved protein of unknown function	unknown function - conserved	unknown	
FTN 0043	Schunoseq	LVSnoseq	47436	48965	510	-	conserved protein of unknown function	unknown function - conserved	unknown	
FTN 0044	Schunoseq	LVSnoseq	49002	49646	215	-	protein of unknown function	unknown function - novel	unknown	
FTN 0045	Schunoseq	LVSnoseq	49665	50741	359	-	protein of unknown function	unknown function - novel	unknown	
FTN 0046	Schunoseq	LVSnoseq	50758	53733	992	-	protein of unknown function	unknown function - novel	unknown	
FTN 0047	Schunoseq	LVSnoseq	53754	57527	1258	-	protein of unknown function	putative enzymes	unknown	
FTN 0048	Schunoseq	LVSnoseq	57540	58757	406	-	conserved protein of unknown function	unknown function - conserved	unknown	
FTN 0049	Schunoseq	LVSnoseq	58770	59591	274	-	hypothetical protein	hypothetical - novel	unknown	
FTN 0050	Schunoseq	LVSnoseq	59569	61089	507	-	protein of unknown function	unknown function - novel	unknown	
FTN 0051	FTT1706	FTL 1165	61102	61707	202	-	conserved protein of unknown function	unknown function - conserved	unknown	
FTN 0052	Schunoseq	LVSnoseq	61717	63777	687	-	protein of unknown function	unknown function - novel	cytoplasm	
FTN 0053	Schunoseq	LVSnoseq	63789	64391	201	-	hypothetical protein	hypothetical - novel	cytoplasm	
FTN 0054	Schunoseq	LVSnoseq	64387	64764	126	-	hypothetical protein	hypothetical - novel	unknown	
FTN 0055	Schunoseq	(FTL_0047-FTL 0048)	65683	64850	278	tyrA	prephenate dehydrogenase	amino acid metabolism - biosynthesis	unknown	
FTN 0056	(FTT1646)	FTL 0049	66388	65954	145	dtd	D-tyrosyl-tRNA(Tyr) deacylase	translation, ribosomal structure and biogenesis	unknown	
FTN 0057	FTT1645	(FTL_0050-FTL 0051)	67894	66665	410	-	major facilitator superfamily (MFS) transport protein	transport	cytoplasmic membrane	
FTN 0058	(FTT1644)	(FTL_0052-FTL 0053)	69628	67916	571	-	beta-fructofuranosidase	carbohydrate metabolism - degradation, utilization, assimilation	unknown	
FTN 0059	Schunoseq	(FTL 1887)	70783	69707	359	leuB	3-isopropylmalate dehydrogenase	amino acid metabolism - biosynthesis	unknown	yes
FTN 0060	Schunoseq	FTL 1888	71354	70788	189	leuD	isopropylmalate isomerase small subunit	amino acid metabolism - biosynthesis	unknown	
FTN 0061	Schunoseq	FTL 1889	72766	71360	469	leuC	isopropylmalate isomerase	amino acid metabolism - biosynthesis	unknown	
FTN 0062	FTT0252	(FTL_0129-FTL 0130)	74348	72771	526	leuA	2-isopropylmalate synthase	amino acid metabolism - biosynthesis	unknown	yes
FTN 0063	FTT0251	FTL 0131	75246	74362	295	ilvE	branched-chain amino acid aminotransferase protein (class IV)	amino acid metabolism - biosynthesis	unknown	
FTN 0064	FTT0250	FTL 0132	78278	75648	877	ppdK	phosphoenolpyruvate synthase/pyruvate phosphate dikinase	energy metabolism	unknown	
FTN 0065	(263437-263752)	(137454-137139)	78869	78558	104	-	protein of unknown function	unknown function - novel	unknown	
FTN 0066	FTT0249	FTL 0133	81317	79071	749	feoB	ferrous iron transport protein B	transport	unknown	
FTN 0067	(FTT0227c-FTT0248)	(FTL_0134-FTL 0184)	82032	81421	204	-	protein of unknown function	unknown function - novel	unknown	yes
FTN 0068	FTT0228c	FTL 0183	82583	82050	178	orn	oligoribonuclease (3'->5' exoribonuclease)	transcription	unknown	
FTN 0069	FTT0229c	FTL 0182	83155	82589	189	efp	elongation factor P	translation, ribosomal structure and biogenesis	unknown	
FTN 0070	FTT0230c	FTL 0181	83603	83199	135	pilE	Type IV pili, pilus assembly protein	motility, attachment and secretion structure	unknown	
FTN 0071	FTT0231c	FTL 0180	84539	83619	307	-	LPS fatty acid acyltransferase	cell wall / LPS / capsule	unknown	
FTN 0072	FTT0232c	FTL 0179	85499	84603	299	-	LPS fatty acid acyltransferase	cell wall / LPS / capsule	unknown	
FTN 0073	FTT0233c	FTL 0178	87161	85509	551	-	conserved membrane protein of unknown function	unknown function - conserved	cytoplasmic membrane	
FTN 0074	(FTT0234c)	FTL 0177	87418	87173	82	-	conserved hypothetical protein	hypothetical - conserved	unknown	
FTN 0075	FTT0235c	FTL 0176	87747	87397	117	rnpA	ribonuclease P protein component	translation, ribosomal structure and biogenesis	cytoplasm	
FTN 0076	FTT0236c	FTL 0175	87871	87740	44	rpmH	50S ribosomal protein L34	translation, ribosomal structure and biogenesis	cytoplasm	
FTN 0077	FTT0237c	FTL 0174	88421	87966	152	-	protein of unknown function	unknown function - novel	unknown	
FTN 0078	FTT0238	FTL 0173	88548	89318	257	aroE	shikimate 5-dehydrogenase	amino acid metabolism - biosynthesis	unknown	
FTN 0079	FTT0239	FTL 0172	89393	90745	451	murC	UDP-N-acetylmuramate-alanine ligase	cell wall / LPS / capsule	unknown	
FTN 0080	FTT0240	FTL 0171	90741	91610	290	-	SAM-dependent methyltransferase	putative enzymes	unknown	
FTN 0081	(FTT0241c)	(173791-FTL 0170)	92358	91627	244	-	protein of unknown function	unknown function - novel	unknown	yes
FTN 0082	FTT0242	FTL 0169	92435	92995	187	-	predicted hydrolase of the HAD superfamily	putative enzymes	cytoplasm	
FTN 0083	FTT0243	FTL 0168	93070	93825	252	-	conserved protein of unknown function	unknown function - conserved	unknown	
FTN 0084	FTT0244	FTL 0167	93832	96570	913	-	DNA/RNA helicase	DNA replication, recombination, modification and repair - replication	unknown	
FTN 0085	FTT0245	FTL 0166	96649	97482	278	uspA	universal stress protein	signal transduction and regulation	cytoplasm	
FTN 0086	(FTT0225c-FTT0246c)	(FTL_0162-FTL 0165)	97601	98827	409	-	metabolite:H+ symporter (MHS) family protein	transport	cytoplasmic membrane	yes
FTN 0087	(FTT0224c)	165732-166346	98862	99476	205	-	allophanate hydrolase subunit 1	other metabolism - degradation, utilization, assimilation	unknown	
FTN 0088	FTT0223c	(FTL 0160)	99479	100174	232	-	protein of unknown function, LamB/YcsF family	unknown function - conserved	unknown	
FTN 0089	FTT0222c	FTL 0159	100182	100991	270	-	allophanate hydrolase subunit 2	other metabolism - degradation, utilization, assimilation	unknown	
FTN 0090	FTT0221	FTL 0158	102545	101004	514	-	acid phosphatase	fatty acids and lipids metabolism	unknown	
FTN 0091	FTT0220c	FTL 0157	102854	103507	218	-	conserved protein of unknown function	unknown function - conserved	cytoplasm	
FTN 0092	FTT0219c	FTL 0156	103528	104520	331	-	inorganic phosphate transporter (PiT) family protein	transport	unknown	
FTN 0093	(FTT0218c)	(160167-160511)	104604	105116	171	-	cytochrome b561 family protein	energy metabolism	unknown	
FTN 0094	(FTT1691-FTT0217)	(FTL 0055)	106073	105129	315	-	transcriptional regulator, LysR family	signal transduction and regulation	unknown	
FTN 0095	FTT1690	FTL 0056	106807	106145	221	-	nitroreductase	energy metabolism	unknown	
FTN 0096	FTT1689c	FTL 0057	107210	107899	230	-	conserved hypothetical membrane protein	hypothetical - conserved	cytoplasmic membrane	
FTN 0097	FTT1688	FTL 0058	109104	107902	401	-	hydroxy/aromatic amino acid permease (HAAAP) family protein	transport - amino-acid	cytoplasmic membrane	

FTN 0098	FTT1687c	FTL 0059	109204	109818	205	gidB	glucose-inhibited cell division protein	cell wall / LPS / capsule	unknown	
FTN 0099	FTT1686c	FTL 0060	109818	110483	222	-	conserved protein of unknown function	unknown function - conserved	unknown	
FTN 0100	(FTT1685)	(FTL_0061- FTL 0061)	111495	110482	338	-	hypothetical membrane protein	hypothetical - novel	unknown	yes
FTN 0101	FTT1684	(FTL_0062- 62252)	112462	111569	298	-	transcriptional regulator, LysR family	signal transduction and regulation	unknown	
FTN 0102	FTT1683c	FTL 0063	112565	113716	384	-	drug:H+ antiporter-1 (DHA1) family protein	transport - drugs / antibacterial compounds	cytoplasmic membrane	
FTN 0103	(FTT1682)	(63762- 66042)	116088	113788	767	-	protein of unknown function	unknown function - novel	unknown	
FTN 0104	FTT1681c	FTL 0068	116266	116859	198	-	phosphoheptose isomerase	carbohydrate metabolism	unknown	
FTN 0105	FTT1680c	FTL 0069	116870	117223	118	-	outer membrane lipoprotein	putative enzymes	unknown	
FTN 0106	FTT1679	FTL 0070	117572	117303	90	rpsT	ribosomal protein S20	translation, ribosomal structure and biogenesis	unknown	
FTN 0107	FTT1678c	FTL 0071	117842	119623	594	lepA	GTP-binding protein LepA	cell wall / LPS / capsule	unknown	
FTN 0108	FTT1677c	FTL 0072	119640	120716	359	trmU	tRNA(5-methylaminomethyl-2-thiouridylate) methyltransferase	translation, ribosomal structure and biogenesis	unknown	
FTN 0109	FTT1676	FTL 0073	121717	120740	326	-	protein of unknown function	unknown function - novel	unknown	
FTN 0110	FTT1675	FTL 0074	122336	121815	174	-	peptide deformylase	translation, ribosomal structure and biogenesis	cytoplasm	
FTN 0111	FTT1674	FTL 0075	122776	122336	147	ribH	riboflavin synthase beta-chain	cofactors, prosthetic groups, electron carriers metabolism	unknown	
FTN 0112	FTT1673	FTL 0076	123997	122789	403	ribAB	3,4-dihydroxy-2-butanone 4-phosphate synthase/GTP cyclohydrolase II	cofactors, prosthetic groups, electron carriers metabolism	unknown	
FTN 0113	FTT1672	FTL 0077	124595	123993	201	ribC	riboflavin synthase alpha chain	cofactors, prosthetic groups, electron carriers metabolism	unknown	
FTN 0114	FTT1671	FTL 0078	125655	124591	355	ribD	pyrimidine reductase/pyrimidine deaminase	cofactors, prosthetic groups, electron carriers metabolism	unknown	
FTN 0115	(FTT1670c- FTT1743)	(FTL 0005)	126046	127293	416	-	Na+/H+ antiporter	transport	cytoplasmic membrane	yes
FTN 0116	FTT1744c	(FTL 0006)	129003	127309	565	ipdC	indolepyruvate decarboxylase	amino acid metabolism - degradation, utilization, assimilation	unknown	
FTN 0117	(FTT1745c)	(FTL 0007)	129457	129107	117	-	ferredoxin	energy metabolism	cytoplasm	yes
FTN 0118	FTT1746	FTL 0008	129531	130451	307	-	serine peptidase, S49 family	post-translational modification, protein turnover, chaperones	unknown	
FTN 0119	FTT1747	FTL 0009	130544	131080	179	-	conserved outer membrane protein of unknown function	unknown function - conserved	outer membrane	
FTN 0120	FTT1748	FTL 0010	131219	131638	140	-	rhodanese-related sulfurtransferase	other metabolism - biosynthesis	unknown	
FTN 0121	FTT1749	FTL 0011	131668	132114	149	secB1	preprotein translocase, subunit B	motility, attachment and secretion structure	unknown	
FTN 0122	FTT1750	FTL 0012	132141	133217	359	recA	recombinase A protein	DNA replication, recombination, modification and repair - restriction/modification	cytoplasm	
FTN 0123	FTT1751	FTL 0013	133207	133656	150	recX	inhibitor of RecA	signal transduction and regulation	unknown	
FTN 0124	FTT1752	FTL 0014	133833	134309	159	ssb	single-strand DNA binding protein	DNA replication, recombination, modification and repair - restriction/modification	cytoplasm	
FTN 0125	FTT1753	FTL 0015	134418	135569	384	ackA	propionate kinase 2 / acetate kinase A	carbohydrate metabolism - degradation, utilization, assimilation	unknown	
FTN 0126	FTT1754	FTL 0016	135582	137675	698	pta	phosphate acetyltransferase	carbohydrate metabolism - degradation, utilization, assimilation	unknown	
FTN 0127	(FTT0005- FTT1755)	(15337- FTL 1642)	137693	139123	477	gabD	succinate semialdehyde dehydrogenase (NAD(P)+ dependent)	amino acid metabolism - degradation, utilization, assimilation	unknown	
FTN 0128	FTT0006	FTL 0021	139163	140368	402	-	metabolite:H+ symporter (MHS) family protein	transport - carbohydrates (sugars, polysaccharides)	cytoplasmic membrane	
FTN 0129	FTT0007	FTL 0020	140546	142321	592	aspS	aspartyl-tRNA synthetase	other metabolism - biosynthesis	unknown	
FTN 0130	(FTT0008)	(FTL 0019)	142461	143402	314	-	glycosyl transferase, group 1	cell wall / LPS / capsule	unknown	
FTN 0131	(FTT0254c- FTT0255c)	(FTL 0136)	144894	143590	435	-	protein of unknown function	unknown function - novel	unknown	
FTN 0132	FTT0256c	FTL 0137	146200	145235	322	-	protein of unknown function	unknown function - novel	unknown	
FTN 0133	FTT0257	FTL 0138	146389	148242	618	-	ribonuclease II family protein	transcription	unknown	
FTN 0134	FTT0258	FTL 0139	148576	149241	222	-	carboxylesterase/phospholipase family protein	putative enzymes	unknown	
FTN 0135	FTT0259	FTL 0140	149241	150140	300	hemC	hydroxymethylbilane synthase (porphobilinogen deaminase)	cofactors, prosthetic groups, electron carriers metabolism	unknown	
FTN 0136	FTT0260	(FTL 0141)	150192	150533	114	crcB	CrcB family protein	cell cycle	unknown	
FTN 0137	FTT0261	FTL 0142	150533	150937	135	-	protein of unknown function	unknown function - novel	unknown	
FTN 0138	(FTT0262- FTT0263)	(148941- FTL 0143)	151080	151694	205	-	hypothetical membrane protein	hypothetical - novel	unknown	
FTN 0139	FTT0264c	FTL 0144	151867	151697	57	-	hypothetical protein	hypothetical - novel	unknown	
FTN 0140	FTT0265	FTL 0145	151997	153793	599	-	ABC-type anion transport system, duplicated permease component	transport	cytoplasmic membrane	
FTN 0141	FTT0266	FTL 0146	153815	155128	438	-	ABC transporter, ATP-binding protein	transport	unknown	
FTN 0142	(FTT0267)	FTL 0147	155218	156756	513	-	hypothetical protein	hypothetical - novel	unknown	
FTN 0143	FTT0268	FTL 0148	156853	158736	628	-	monovalent cation:proton antiporter	transport	cytoplasmic membrane	
FTN 0144	FTT0269	FTL 0149	158724	159260	179	paaY	carbonic anhydrases/acetyltransferases, isoleucine patch superfamily	putative enzymes	unknown	
FTN 0145	FTT0270	FTL 0150	159214	159855	214	lolB	outer membrane lipoprotein LolB	cell wall / LPS / capsule	unknown	
FTN 0146	FTT0271	FTL 0151	159846	160670	275	ispE	4-diphosphocytidyl-2-C-methyl-D-erythritol kinase	cofactors, prosthetic groups, electron carriers metabolism	unknown	
FTN 0148	(FTT0272- FTT0172)	(FHF0880.1)	160862	161488	209	-	hypothetical membrane protein	hypothetical - novel	cytoplasmic membrane	
FTN 0149	(FTT0173)	(FTL 1923)	161561	162472	304	-	conserved protein of unknown function	unknown function - conserved	unknown	yes
FTN 0150	FTT0174	FTL 1922	162491	163057	189	-	YGGT family membrane protein	unknown function - conserved	cytoplasmic membrane	
FTN 0151	FTT0175c	FTL 1921	163836	163057	260	-	ABC-type nitrate/sulfonate/bicarbonate transport system, ATPase component	transport	unknown	
FTN 0152	(FTT0176c)	(1852905- 1854525)	165589	163868	574	-	ABC-type anion transport system, duplicated permease component	transport	cytoplasmic membrane	
FTN 0153	FTT0177c	(FTL 1918)	166443	166021	141	-	RimI-like acetyltransferase	putative enzymes	unknown	
FTN 0154	FTT0178c	(FTL 1917)	167899	166433	489	rimK	glutathione synthase/ribosomal protein S6 modification enzyme	translation, ribosomal structure and biogenesis	unknown	
FTN 0155	(FTT0179)	FTL 1916	168063	170075	671	-	competence protein	transport	unknown	
FTN 0156	FTT0180	FTL 1915	170179	171063	295	plsC	1-acylglycerol-3-phosphate acyltransferase	fatty acids and lipids metabolism	unknown	
FTN 0157	FTT0181c	FTL 1914	172004	171471	178	-	membrane protein of unknown function	unknown function - novel	cytoplasmic membrane	
FTN 0158	FTT0182c	FTL 1913	172644	172105	180	-	Sua5/YciO/YrdC family protein	translation, ribosomal structure and biogenesis	unknown	
FTN 0159	FTT0183c	FTL 1912	174437	172770	556	rpsA	30S ribosomal protein S1	translation, ribosomal structure and biogenesis	unknown	
FTN 0160	FTT0184	FTL 1911	174585	175043	153	-	zinc-binding protein	putative enzymes	unknown	
FTN 0161	FTT0185	FTL 1910	175050	175937	296	ddlB	D-alanine-D-alanine ligase B	cell wall / LPS / capsule	unknown	
FTN 0162	FTT0186	FTL 1909	175937	176617	227	ftsQ	cell division protein FtsQ	cell cycle	unknown	
FTN 0163	FTT0187	FTL 1908	176722	177981	420	ftsA	cell division protein FtsA	cell cycle	cytoplasm	
FTN 0164	FTT0188	FTL 1907	178028	179170	381	ftsZ	cell division protein FtsZ	cell cycle	cytoplasm	
FTN 0165	FTT0189	FTL 1906	179199	180053	285	lpxC	UDP-3-O-acyl-N-acetylglucosamine deacetylase	cell wall / LPS / capsule	unknown	
FTN 0166	FTT0190c	FTL 1905	181710	180073	546	dnaX	DNA polymerase III, gamma/tau subunits	DNA replication, recombination, modification and repair - restriction/modification	cytoplasm	
FTN 0167	FTT0191	FTL 1904	181982	182956	325	prfB	peptide chain release factor 2	translation, ribosomal structure and biogenesis	unknown	
FTN 0168	FTT0192	FTL 1903	183091	184818	576	lysU	lysyl-tRNA synthetase	other metabolism - biosynthesis	unknown	
FTN 0169	FTT0193c	FTL 1902	185747	185208	180	-	conserved hypothetical membrane protein	hypothetical - conserved	cytoplasmic membrane	
FTN 0170	FTT0194c	(FTL 1901)	186718	185849	290	-	conserved hypothetical membrane protein	hypothetical - conserved	cytoplasmic membrane	
FTN 0171	FTT0195c	FTL 1900	188268	186730	513	glsA	glutaminase	amino acid metabolism - degradation, utilization, assimilation	unknown	
FTN 0172	FTT0196c	FTL 1899	189387	188353	345	glnA	glutamine synthetase	other metabolism - degradation, utilization, assimilation	unknown	
FTN 0173	FTT0197c	FTL 1898	190484	189510	325	holA	DNA polymerase III, delta subunit	DNA replication, recombination, modification and repair - restriction/modification	cytoplasm	
FTN 0174	(FTT0198)	FTL 1897	190570	191085	172	blc	outer membrane lipoprotein	cell wall / LPS / capsule	outer membrane	
FTN 0175	(FTT0199- FTT0200)	(FTL 1896)	191251	192873	541	-	hypothetical protein	hypothetical - novel	unknown	yes
FTN 0176	(FTT0201)	(1858996- 1859876)	193022	194287	422	-	serine permease	transport - amino-acid	cytoplasmic membrane	yes
FTN 0177	FTT0203c	FTL 1929	195903	194359	515	purH	AICAR transformylase/IMP cyclohydrolase	nucleotides and nucleosides metabolism	unknown	
FTN 0178	FTT0204	FTL 1930	196080	197363	428	purA	adenylosuccinate synthetase	nucleotides and nucleosides metabolism	unknown	
FTN 0179	FTT0205	FTL 1931	197370	197900	177	hpt	hypoxanthine-guanine phosphoribosyltransferase	nucleotides and nucleosides metabolism	unknown	
FTN 0180	(FTT0206c)	(1863624- 1864334)	198641	197925	239	-	dienelactone hydrolase family protein	putative enzymes	unknown	
FTN 0181	FTT0207c	FTL 1934	199581	198742	280	-	manganese/zinc/iron chelate uptake transporter (MZT) family protein	transport	unknown	
FTN 0182	FTT0208c	FTL 1935	200200	199526	225	-	ATP-binding cassette (ABC) superfamily protein	transport	cytoplasmic membrane	

FTN 0183	FTT0209c	FTL 1936	201119	200205	305	-	manganese/Zinc/Iron chelate uptake transporter family protein	transport	unknown	
FTN 0184	(FTT0210c)	(1866890-1868103)	202417	201197	407	-	major facilitator superfamily (MFS) transport protein	transport	unknown	
FTN 0185	FTT0211c	FTL 1939	203002	202541	154	-	outer membrane protein	cell wall / LPS / capsule	unknown	
FTN 0186	FTT0212c	FTL 1940	203609	203016	198	wrbA	trp repressor binding protein	signal transduction and regulation	unknown	
FTN 0187	FTT0213	FTL 1941	203616	204809	398	rbn	tRNA processing ribonuclease BN	nucleotides and nucleosides metabolism	unknown	
FTN 0188	(FTT0214)	FTL 1942	204831	205403	191	-	nicotinamide ribonucleoside (NR) uptake permease (PnuC) family protein	transport	unknown	
FTN 0189	FTT0215	FTL 1943	205403	207553	717	priA	primosomal protein N	DNA replication, recombination, modification and repair - restriction/modification	unknown	
FTN 0190	(FTT0274-FTT0275c)	(FTL 0186)	208801	207626	392	-	drug:H+ antiporter-1 (DHA1) family protein	transport - drugs / antibacterial compounds	cytoplasmic membrane	
FTN 0191	(FTT0276c)	FTL 0187	209609	208872	246	-	polar amino acid uptake transporter	transport - amino-acid	unknown	
209786-209691	FTT0277c	187216-187311	209786	209691						
FTN 0192	FTT0278c	FTL 0188	210995	209805	397	cydB	cytochrome bd-I terminal oxidase subunit II	energy metabolism	unknown	
FTN 0193	FTT0279c	FTL 0189	212760	211009	584	cydA	cytochrome bd-I terminal oxidase subunit I	energy metabolism	unknown	
FTN 0194	FTT0280c	FTL 0190	214584	213220	455	-	major facilitator superfamily (MFS) transport protein	transport	cytoplasmic membrane	
FTN 0195	FTT0281	FTL 0191	216593	217492	300	cyoA	cytochrome bo terminal oxidase subunit II	energy metabolism	unknown	
FTN 0196	FTT0282	FTL 0192	217534	219561	676	cyoB	cytochrome bo terminal oxidase subunit I	energy metabolism	cytoplasmic membrane	
FTN 0197	FTT0283	FTL 0193	219561	220160	200	cyoC	cytochrome bo terminal oxidase subunit III	energy metabolism	unknown	
FTN 0198	FTT0284	FTL 0194	220165	220494	110	cyoD	cytochrome bo terminal oxidase subunit IV	energy metabolism	unknown	
FTN 0199	FTT0285	FTL 0195	220528	221373	282	cyoE	heme O synthase	cofactors, prosthetic groups, electron carriers metabolism	unknown	
FTN 0200	FTT0286c	FTL 0196	222564	221524	347	-	UDP-3-O-[3-fatty acid] glucosamine N-acyltransferase	fatty acids and lipids metabolism	unknown	
FTN 0201	FTT0287c	FTL 0197	223334	222642	231	-	conserved hypothetical protein	hypothetical - conserved	unknown	
FTN 0202	FTT0288c	FTL 0198	224185	223337	283	pdxY	pyridoxal kinase	putative enzymes	unknown	
FTN 0203	FTT0289c	FTL 0199	224626	224195	144	-	protein of unknown function	unknown function - novel	unknown	
FTN 0204	FTT0290	FTL 0200	224772	225725	318	-	MoxR-like ATPase	putative enzymes	unknown	
FTN 0205	FTT0291	FTL 0201	225741	226649	303	-	conserved hypothetical protein	hypothetical - conserved	unknown	
FTN 0206	FTT0292	FTL 0202	226642	227115	158	-	protein of unknown function	unknown function - novel	unknown	
FTN 0207	FTT0293	FTL 0203	227115	228113	333	-	protein of unknown function containing a von Willebrand factor type A (vWA) domain	unknown function - conserved	unknown	
FTN 0208	(FTT0294)	FTL 0204	228118	229113	332	-	hypothetical membrane protein with von Willebrand factor type A domain	hypothetical - novel	cytoplasmic membrane	
FTN 0209	Schunoseq	(FTL 0205)	229107	229973	289	-	protein of unknown function with TPR repeat region and von Willebrand factor type A domain	unknown function - conserved	unknown	
FTN 0210	FTT0295	FTL 0206	229966	231588	541	-	conserved protein of unknown function	unknown function - conserved	cytoplasmic membrane	
FTN 0211	FTT0296	FTL 0207	231598	232263	222	pcp	pyrrolidone carboxylylate peptidase	post-translational modification, protein turnover, chaperones	unknown	
FTN 0212	FTT0297	FTL 0208	232274	232753	160	-	hypothetical membrane protein	hypothetical - novel	cytoplasmic membrane	
FTN 0213	FTT0298	FTL 0209	232787	233224	146	holC	DNA polymerase III, chi subunit	DNA replication, recombination, modification and repair - restriction/modification	cytoplasm	
FTN 0214	FTT0299	FTL 0210	234222	236978	919	valS	valyl-tRNA synthetase	other metabolism - biosynthesis	unknown	
FTN 0215	Schunoseq	LVSnoseq	237281	237943	221	-	hypothetical protein	hypothetical - novel	cytoplasm	
FTN 0216	FTT0302	(FTL 0213)	238094	238426	111	-	hypothetical membrane protein	hypothetical - novel	cytoplasmic membrane	
FTN 0217	FTT0303c	FTL 0214	239583	238429	385	-	L-lactate dehydrogenase	other metabolism - degradation, utilization, assimilation	unknown	
FTN 0218	FTT0304c	FTL 0215	241126	240476	217	nfnB	dihydropteridine reductase	energy metabolism	unknown	
FTN 0219	FTT0305	FTL 0216	241266	241640	125	-	MutT/nudix family protein	putative enzymes	unknown	
FTN 0220	FTT0306	FTL 0217	241817	243205	463	fumC	fumarate hydratase, class II	energy metabolism	unknown	
FTN 0221	FTT0307	FTL 0218	243339	244742	468	gltX	glutamyl-tRNA synthetase	other metabolism - biosynthesis	unknown	
FTN 0222	FTT0308	(219643-FTL 0219)	244745	245710	322	-	protein of unknown function	unknown function - novel	unknown	
FTN 0223	(FTT0310)	(FTL 0221)	246583	248118	512	-	amino acid-polyamine-organocation (APC) superfamily protein	transport - amino-acid	cytoplasmic membrane	yes
FTN 0225	FTT0311c	FTL 0222	249375	248455	307	-	conserved protein of unknown function	unknown function - conserved	cytoplasm	
FTN 0226	(FTT0312c)	FTL 0223	249999	249460	180	folA	dihydrofolate reductase type I	cofactors, prosthetic groups, electron carriers metabolism	unknown	
FTN 0227	FTT0313	FTL 0224	250191	250907	239	rpsB	30S ribosomal protein S2	translation, ribosomal structure and biogenesis	unknown	
FTN 0228	FTT0314	FTL 0225	250932	251798	289	tsf	protein chain elongation factor EF-Ts	translation, ribosomal structure and biogenesis	unknown	
FTN 0229	FTT0315	FTL 0226	251805	252551	249	pyrH	uridylate kinase	nucleotides and nucleosides metabolism	unknown	
FTN 0230	FTT0316	FTL 0227	252583	253137	185	frr	ribosome recycling factor	translation, ribosomal structure and biogenesis	unknown	
FTN 0231	FTT0317	FTL 0228	253173	253943	257	uppS	undecaprenyl pyrophosphate synthetase	cofactors, prosthetic groups, electron carriers metabolism	unknown	
FTN 0232	FTT0318	FTL 0229	253960	254751	264	cdsA	phosphatidate cytidylyltransferase	fatty acids and lipids metabolism	unknown	
FTN 0233	FTT0319	FTL 0230	254764	255207	148	dut	dUTP pyrophosphatase (Deoxyuridine 5'- triphosphate nucleotidohydrolase)	nucleotides and nucleosides metabolism	unknown	
FTN 0234	FTT0320	FTL 0231	255214	255807	198	pgsA	phosphatidylglycerophosphate synthetase	fatty acids and lipids metabolism	unknown	
FTN 0235	FTT0321	FTL 0232	255945	256316	124	rpsL	30S ribosomal protein S12	translation, ribosomal structure and biogenesis	unknown	
FTN 0236	FTT0322	FTL 0233	256358	256828	157	rpsG	30S ribosomal protein S7	translation, ribosomal structure and biogenesis	unknown	
FTN 0237	FTT0323	FTL 0234	256846	258957	704	fusA	elongation factor G (EF-G)	translation, ribosomal structure and biogenesis	unknown	
FTN 0238	FTT0324	FTL 0235	258981	259295	105	rpsJ	30S ribosomal protein S10	translation, ribosomal structure and biogenesis	unknown	
FTN 0239	FTT0325	FTL 0236	259402	260031	210	rplC	50S ribosomal protein L3	translation, ribosomal structure and biogenesis	unknown	
FTN 0240	FTT0326	FTL 0237	260067	260687	207	rplD	50S ribosomal protein L4	translation, ribosomal structure and biogenesis	unknown	
FTN 0241	FTT0327	FTL 0238	260687	260983	99	rplW	50S ribosomal protein L23	translation, ribosomal structure and biogenesis	cytoplasm	
FTN 0242	FTT0328	FTL 0239	261008	261829	274	rplB	50S ribosomal protein L2	translation, ribosomal structure and biogenesis	cytoplasm	
FTN 0243	FTT0329	FTL 0240	261847	262122	92	rpsS	30S ribosomal protein S19	translation, ribosomal structure and biogenesis	cytoplasm	
FTN 0244	FTT0330	FTL 0241	262141	262473	111	rplV	50S ribosomal protein L22	translation, ribosomal structure and biogenesis	cytoplasm	
FTN 0245	FTT0331	FTL 0242	262491	263159	223	rpsC	30S ribosomal protein S3	translation, ribosomal structure and biogenesis	cytoplasm	
FTN 0246	FTT0332	FTL 0243	263162	263572	137	rplP	50S ribosomal protein L16	translation, ribosomal structure and biogenesis	cytoplasm	
FTN 0248	FTT0334	FTL 0245	263788	264036	83	rpsQ	30S ribosomal protein S17	translation, ribosomal structure and biogenesis	cytoplasm	
FTN 0249	FTT0335	FTL 0246	264131	264496	122	rplN	50S ribosomal protein L14	translation, ribosomal structure and biogenesis	unknown	
FTN 0250	FTT0336	FTL 0247	264521	264835	105	rplX	50S ribosomal protein L24	translation, ribosomal structure and biogenesis	unknown	
FTN 0251	FTT0337	FTL 0248	264850	265386	179	rplE	50S ribosomal protein L5	translation, ribosomal structure and biogenesis	unknown	
FTN 0252	FTT0338	FTL 0249	265408	265710	101	rpsN	30S ribosomal protein S14	translation, ribosomal structure and biogenesis	unknown	
FTN 0253	FTT0339	FTL 0250	265730	266125	132	rpsH	30S ribosomal protein S8	translation, ribosomal structure and biogenesis	unknown	
FTN 0254	FTT0340	FTL 0251	266147	266680	178	rplF	50S ribosomal protein L6	translation, ribosomal structure and biogenesis	unknown	
FTN 0255	FTT0341	FTL 0252	266706	267056	117	rplR	50S ribosomal protein L18	translation, ribosomal structure and biogenesis	unknown	
FTN 0256	FTT0342	FTL 0253	267087	267584	166	rpsE	30S ribosomal protein S5	translation, ribosomal structure and biogenesis	unknown	
FTN 0257	FTT0343	FTL 0254	267594	267776	61	rpmD	50S ribosomal protein L30	translation, ribosomal structure and biogenesis	unknown	
FTN 0258	FTT0344	FTL 0255	267786	268214	143	rplO	50S ribosomal protein L15	translation, ribosomal structure and biogenesis	unknown	
FTN 0259	FTT0345	FTL 0256	268228	269550	441	secY	preprotein translocase, subunit Y, membrane protein	motility, attachment and secretion structure	cytoplasmic membrane	
FTN 0260	FTT0346	244587-244700	269577	269687	37	rpmJ	50S ribosomal protein L36	translation, ribosomal structure and biogenesis	unknown	
FTN 0261	FTT0347	FTL 0258	269807	270160	118	rpsM	30S ribosomal protein S13	translation, ribosomal structure and biogenesis	unknown	
FTN 0262	FTT0348	FTL 0259	270202	270588	129	rpsK	30S ribosomal protein S11	translation, ribosomal structure and biogenesis	unknown	
FTN 0263	FTT0349	FTL 0260	270613	271230	206	rpsD	30S ribosomal protein S4	translation, ribosomal structure and biogenesis	unknown	

FTN 0264	FTT0350	FTL 0261	271291	272259	323	rpoA	DNA-directed RNA polymerase, alpha subunit	transcription	cytoplasm	
FTN 0265	FTT0351	FTL 0262	272307	272741	145	rplQ	50S ribosomal protein L17	translation, ribosomal structure and biogenesis	unknown	
FTN 0266	FTT0356	FTL 0267	272930	274813	628	htpG	chaperone Hsp90, heat shock protein HtpG	post-translational modification, protein turnover, chaperones - chaperones	unknown	
FTN 0267	Schunoseq	LVSnoseq	274900	275400	167	-	protein of unknown function	unknown function - novel	cytoplasm	
(FTN_0268)	partialduplica tion	partialduplicat ion	275553	275780						
FTN 0269	FTT0375	(FTL_1312- FTL 1313)	277422	275914	503	-	C4-dicarboxylate anaerobic carrier	transport	cytoplasmic membrane	
FTN 0270	FTT0374c	FTL 1311	277740	279377	546	pyrG	CTP synthase (UTP-ammonia lyase)	nucleotides and nucleosides metabolism	unknown	
FTN 0271	FTT0373c	FTL 1310	279453	279872	140	ndk	nucleoside diphosphate kinase	nucleotides and nucleosides metabolism	unknown	
FTN 0272	FTT0372c	FTL 1309	279916	280815	300	accD	acetyl-CoA carboxylase, carboxytransferase subunit beta	fatty acids and lipids metabolism	unknown	
FTN 0273	FTT0371c	FTL 1308	280833	282011	393	folC	folylpoly-gamma-glutamate synthetase/ dihydrofolate synthetase	cofactors, prosthetic groups, electron carriers metabolism	unknown	
FTN 0274	FTT0370c	FTL 1307	281992	282399	136	-	conserved protein of unknown function	unknown function - conserved	unknown	
FTN 0275	FTT0369c	FTL 1306	282485	283540	352	-	conserved protein of unknown function	unknown function - conserved	unknown	
FTN 0276	FTT0368c	FTL 1305	283566	285104	513	mviN	multidrug/oligosaccharidyl-lipid/polysaccharide (MOP) transporter	transport - drugs / antibacterial compounds	cytoplasmic membrane	
FTN 0277	FTT0367c	FTL 1304	285160	286662	501	gshA	glutamate-cysteine ligase	cofactors, prosthetic groups, electron carriers metabolism	unknown	
FTN 0278	FTT0366	FTL 1303	287093	286881	71	rpmE	50S ribosomal protein L31	translation, ribosomal structure and biogenesis	unknown	
FTN 0279	FTT0365	FTL 1302	287946	287218	243	-	oxidoreductase	energy metabolism	unknown	
FTN 0280	Schunoseq	FTL 1300	288029	288421	131	-	hypothetical protein	hypothetical - novel	unknown	
FTN 0281	FTT0364c	FTL 1299	288433	288879	149	-	protein of unknown function	unknown function - novel	unknown	
FTN 0282	Schunoseq	LVSnoseq	288913	289806	298	-	conserved protein of unknown function	unknown function - conserved	unknown	
(FTN_0283)	Schunoseq	LVSnoseq	289901	291434						
FTN 0284	Schunoseq	LVSnoseq	291793	292794	334	-	prophage maintenance system killer protein (DOC)	mobile and extrachromosomal element functions - phage or plasmid related proteins	unknown	
FTN 0285	Schunoseq	LVSnoseq	292790	294007	406	-	type I restriction-modification system, subunit S	DNA replication, recombination, modification and repair - restriction/modification	unknown	
FTN 0286	Schunoseq	LVSnoseq	294223	294783	187	-	transposase	mobile and extrachromosomal element functions - transposition	unknown	
FTN 0287	(FTT1641c)	(FTL 1711)	294845	295609	255	-	type I restriction-modification system, subunit R (restriction)	DNA replication, recombination, modification and repair - restriction/modification	unknown	yes
FTN 0289	(FTT1640c)	FTL 1710	296016	296714	233	proQ	activator of osmoprotectant transporter ProP	signal transduction and regulation	unknown	
FTN 0290	FTT1639c	FTL 1709	296739	297281	181	-	protein of unknown function	unknown function - novel	unknown	
FTN 0291	FTT1638	FTL 1708	298753	297302	484	trkH	K+ transporter (Trk) family protein	transport	cytoplasmic membrane	
FTN 0292	FTT1637c	FTL 1707	298865	299065	67	-	protein of unknown function	unknown function - novel	unknown	
FTN 0293	FTT1636	FTL 1706	300084	299470	205	lolA	lipoprotein releasing system, subunit A, outer membrane lipoproteins carrier	cell wall / LPS / capsule	unknown	
FTN 0294	FTT1635	FTL 1705	302586	300088	833	ftsK	cell division protein FtsK	cell cycle	unknown	
FTN 0295	FTT1634c	FTL 1704	302708	303373	222	-	phosphatidic acid phosphatase, PAP2 superfamily	putative enzymes	unknown	
FTN 0296	FTT1633c	FTL 1703	303623	305074	484	lysP	lysine:H+ symporter	transport - amino-acid	cytoplasmic membrane	
FTN 0297	FTT1632c	FTL 1702	305086	305721	212	-	conserved protein of unknown function	unknown function - conserved	unknown	
FTN 0298	FTT1631c	FTL 1701	305744	306727	328	glpX	fructose 1,6-bisphosphatase II	energy metabolism	unknown	
FTN 0299	FTT1630c	FTL 1700	306996	308501	502	putP	proline:Na+ symporter	transport - amino-acid	cytoplasmic membrane	
FTN 0300	FTT1629c	FTL 1699	308570	310246	559	-	glycosyl transferase, group 2	cell wall / LPS / capsule	cytoplasmic membrane	
FTN 0301	FTT1628c	(FTL_1697- FTL 1698)	310260	311564	435	-	transporter-associated protein, HlyC/CorC family	transport	unknown	
FTN 0302	(1690082- 1690428)	FTL 1696	311638	311982	115	-	hypothetical protein	hypothetical - novel	cytoplasmic membrane	
FTN 0303	FTT1627c	FTL 1695	311969	312892	308	-	pilus assembly protein	motility, attachment and secretion structure	unknown	
FTN 0304	FTT1626c	(FTL 1694)	312892	314295	468	-	pilus assembly protein	motility, attachment and secretion structure	outer membrane	
FTN 0305	FTT1625c	(FTL 1693)	314309	314926	206	-	pilus assembly protein	motility, attachment and secretion structure	unknown	
FTN 0306	(FTT1623c- FTT1624c)	(FTL_1691- FTL 1692)	315040	315714	225	-	pilus assembly protein	motility, attachment and secretion structure	unknown	yes
FTN 0307	FTT1622c	FTL 1690	315736	316653	306	-	pilus assembly protein	motility, attachment and secretion structure	unknown	
FTN 0308	(FTT1621c)	(FTL_1682- FTL 1689)	316669	318474	602	-	membrane protein of unknown function	unknown function - novel	outer membrane	yes
FTN 0309	Schunoseq	LVSnoseq	318501	320303	601	-	hypothetical membrane protein	hypothetical - novel	outer membrane	
FTN 0310	FTT1616	FTL 1683	320389	321765	459	cysS	cysteinyl-tRNA synthetase	other metabolism - biosynthesis	unknown	
FTN 0311	FTT1617	FTL 1684	321772	322713	314	-	modification methylase, HemK family	translation, ribosomal structure and biogenesis	unknown	
FTN 0312	(FTT1618)	FTL 1685	322772	323959	396	-	drug:H+ antiporter-1 (DHA1) family protein	transport - drugs / antibacterial compounds	cytoplasmic membrane	
FTN 0313	(FTT1619)	FTL 1686	323976	324464	163	-	acetyltransferase	putative enzymes	unknown	
FTN 0315	Schunoseq	LVSnoseq	325591	324848	248	-	traT-like protein	mobile and extrachromosomal element functions - phage or plasmid related proteins	outer membrane	
FTN 0316	Schunoseq	LVSnoseq	326791	325739	351	-	alanine racemase	amino acid metabolism - degradation, utilization, assimilation	unknown	
FTN 0317	(FTT1585)	FTL 1642	326808	328169	454	-	amino acid transporter	transport - amino-acid	unknown	
FTN 0318	FTT1586c	FTL 1641	328455	328177	93	-	hypothetical protein	hypothetical - novel	unknown	
FTN 0319	(FTT1587c)	FTL 1640	329970	328471	500	-	amino acid-polyamine-organocation family protein	transport - amino-acid	cytoplasmic membrane	
FTN 0320	FTT1589c	FTL 1639	331079	330339	247	-	protein of unknown function	unknown function - novel	unknown	
FTN 0321	FTT1590c	FTL 1638	331777	331085	231	ubiG	3-demethylubiquinone 3-methyltransferase/ 2-octaprenyl-6-hydroxyphenol methylase	cofactors, prosthetic groups, electron carriers metabolism	unknown	
FTN 0322	FTT1591	FTL 1637	331919	333016	366	-	VacJ like lipoprotein	cell wall / LPS / capsule	cytoplasmic membrane	
FTN 0323	FTT1613	FTL 0512	333474	333241	78	-	BolA family protein	transcription	unknown	
FTN 0324	FTT1612	FTL 0513	333784	333494	97	-	conserved hypothetical protein	hypothetical - conserved	unknown	
FTN 0325	FTT1611	FTL 0514	334446	333787	220	-	membrane protein of unknown function	unknown function - conserved	cytoplasmic membrane	
FTN 0326	(FTT1610)	FTL 0515	335054	334524	177	-	conserved hypothetical protein	hypothetical - conserved	unknown	
FTN 0327	FTT1609	FTL 0516	335818	335057	254	-	ABC-type transport system permease protein	transport	unknown	
FTN 0328	FTT1608	FTL 0517	336625	335825	267	-	ATP-binding cassette (ABC) superfamily protein	transport	unknown	
FTN 0329	FTT1607	FTL 0518	336911	336642	90	minE	cell division topological specificity factor protein	cell cycle	unknown	
FTN 0330	FTT1606	FTL 0519	337738	336917	274	minD	septum formation inhibitor-activating ATPase	cell cycle	unknown	
FTN 0331	FTT1605	FTL 0520	338464	337781	228	minC	septum formation inhibitor	cell cycle	unknown	
FTN 0332	FTT1604	FTL 0521	338654	338502	51	rpmG	50S ribosomal protein L33	translation, ribosomal structure and biogenesis	unknown	
FTN 0333	FTT1603	FTL 0522	338917	338684	78	rpmB	50S ribosomal protein L28	translation, ribosomal structure and biogenesis	unknown	
FTN 0334	FTT1602	FTL 0523	339395	339054	114	-	conserved protein of unknown function	unknown function - conserved	periplasm	
FTN 0335	FTT1601c	FTL 0524	339525	341561	679	recG	ATP-dependent DNA helicase	DNA replication, recombination, modification and repair - restriction/modification	cytoplasm	
FTN 0336	1668698- 1668501	504412- 504606	341568	341762	65	-	hypothetical protein	hypothetical - novel	unknown	
FTN 0337	FTT1600c	FTL 0525	342333	343844	504	fumA	fumarate hydratase, class I	energy metabolism	unknown	
FTN 0338	FTT0823	FTL 0315	344391	345035	215	-	MutT/nudix family protein	putative enzymes	unknown	
FTN 0339	FTT0824	FTL 0316	345056	345406	117	-	arsenate reductase	cofactors, prosthetic groups, electron carriers metabolism	unknown	
FTN 0340	FTT0825c	FTL 0317	345768	345436	111	-	protein of unknown function	unknown function - novel	unknown	
FTN 0341	FTT0826c	FTL 0318	346190	345948	81	-	protein of unknown function	unknown function - novel	unknown	
FTN 0342	FTT0827c	FTL 0319	347585	346275	437	-	permease	transport	cytoplasmic membrane	
FTN 0343	(FTT0828c)	FTL 0320	349238	347655	528	-	aminotransferase	amino acid metabolism	unknown	
FTN 0344	FTT0829c	(307504- 309153)	350966	349254	571	-	aspartate:alanine exchanger (AAE) family protein	transport - amino-acid	cytoplasmic membrane	
FTN 0345	(FTT0830c)	(309375- 310408)	352147	351044	368	-	DNA uptake protein, SMF family	transport	unknown	
FTN 0346	FTT0831c	FTL 0325	353400	352150	417	-	OmpA family protein	cell wall / LPS / capsule	unknown	
FTN 0347	FTT0832	FTL 0326	353592	354053	154	fkpB	FKBP-type peptidyl-prolyl cis-trans isomerase	post-translational modification, protein turnover, chaperones - protein modification	unknown	
FTN 0348	FTT0833	FTL 0327	354037	354996	320	lytB	1-hydroxy-2-methyl-2-(E)-butenyl 4- diphosphate reductase	cofactors, prosthetic groups, electron carriers metabolism	unknown	
FTN 0349	FTT0834	FTL 0328	355001	355360	120	aroH	chorismate mutase	amino acid metabolism - biosynthesis	unknown	
FTN 0350	FTT0835	FTL 0329	355372	356190	273	pssA	phosphatidylserine synthase	fatty acids and lipids metabolism	unknown	
FTN 0351	FTT0836c	(FTL 0330)	356422	356201	74	-	hypothetical protein	hypothetical - novel	unknown	
FTN 0352	FTT0837	FTL 0331	356574	357278	235	tolQ	group A colicin translocation; tolQ protein	transport - drugs / antibacterial compounds	cytoplasmic membrane	
FTN 0353	FTT0838	FTL 0332	357297	357734	146	tolR	group A colicin translocation; tolR protein	transport - drugs / antibacterial compounds	cytoplasmic membrane	
FTN 0354	FTT0839	FTL 0333	357724	358632	303	tolA	group A colicin translocation; tolA protein	transport - drugs / antibacterial compounds	cytoplasmic membrane	

FTN 0355	FTT0840	FTL 0334	358647	359951	435	tolB	group A colicin translocation; tolB protein	transport - drugs / antibacterial compounds	periplasm	
FTN 0356	FTT0841	FTL 0335	359964	360206	81	-	protein of unknown function	unknown function - novel	unknown	
FTN 0357	FTT0842	FTL 0336	360228	360848	207	pal	peptidoglycan-associated lipoprotein, OmpA family	transport - drugs / antibacterial compounds	outer membrane	
FTN 0358	(FTT0843)	FTL 1686	360958	362274	439	-	tRNA-methylthiotransferase MiaB protein	translation, ribosomal structure and biogenesis	unknown	
FTN 0359	(FTT0844)	LVSnoseq	362380	363099	240	-	dehydrogenase related to short-chain alcohol dehydrogenases	putative enzymes	unknown	
FTN 0360	FTT0845	LVSnoseq	363126	363353	76	-	hypothetical protein	hypothetical - novel	unknown	
FTN 0361	FTT0846	(322045-FTL 0342)	363337	364833	499	-	deoxyribodipyrimidine photolyase	DNA replication, recombination, modification and repair - restriction/modification	cytoplasm	
FTN 0362	FTT0847	FTL 0343	364836	366362	509	-	deoxyribodipyrimidine photolyase-related protein	putative enzymes	unknown	
FTN 0363	FTT0849	FTL 0345	366625	367551	309	-	sodium bile acid symporter family protein	transport	unknown	
FTN 0364	FTT0850	(FTL 0346)	367604	368224	207	-	conserved protein of unknown function	unknown function - conserved	unknown	
FTN 0365	FTT0851	(FTL_0347-FTL 0347)	368251	369006	252	-	conserved hypothetical membrane protein	hypothetical - conserved	cytoplasmic membrane	
FTN 0367	Schunoseq	LVSnoseq	369322	370554	411	-	phage integrase	DNA replication, recombination, modification and repair - restriction/modification	unknown	
FTN 0368	Schunoseq	LVSnoseq	371729	372250	174	-	hypothetical protein	hypothetical - novel	unknown	
FTN 0369	Schunoseq	LVSnoseq	372857	373084	76	-	protein of unknown function	unknown function - novel	cytoplasm	
FTN 0370	Schunoseq	LVSnoseq	373090	373365	92	-	conserved hypothetical protein	hypothetical - conserved	unknown	
FTN 0371	Schunoseq	LVSnoseq	373472	374326	285	-	protein of unknown function	unknown function - novel	unknown	
FTN 0372	Schunoseq	LVSnoseq	374491	374706	72	-	regulatory protein, AlpA family	signal transduction and regulation	unknown	
FTN 0373	Schunoseq	LVSnoseq	374890	376083	398	-	phage integrase	mobile and extrachromosomal element functions - phage or plasmid related proteins	unknown	
FTN 0374	Schunoseq	LVSnoseq	376108	376314	69	-	hypothetical protein	hypothetical - novel	unknown	
FTN 0375	Schunoseq	LVSnoseq	376636	377250	205	-	hypothetical protein	hypothetical - novel	unknown	
FTN 0376	Schunoseq	LVSnoseq	378139	379017	293	-	conserved hypothetical protein	hypothetical - conserved	unknown	
FTN 0378	Schunoseq	LVSnoseq	379010	380461	484	-	hypothetical protein	hypothetical - novel	unknown	
FTN 0379	Schunoseq	LVSnoseq	380755	381126	124	-	hypothetical protein	hypothetical - novel	unknown	
FTN 0380	Schunoseq	LVSnoseq	381374	381616	81	-	conserved hypothetical protein	hypothetical - conserved	cytoplasm	
FTN 0381	(FTT0852)	(328262-329245)	382197	383180	328	-	protein of unknown function	unknown function - novel	unknown	
FTN 0382	FTT0853	(329419-330571)	383357	384592	412	-	arsenite-antimonite (ArsB) efflux family protein	transport	cytoplasmic membrane	
FTN 0383	FTT0854c	FTL 0352	384997	384611	129	-	conserved hypothetical membrane protein	hypothetical - conserved	cytoplasmic membrane	
FTN 0384	FTT0855c	FTL 0354	385376	385020	119	-	conserved hypothetical protein	hypothetical - conserved	unknown	
FTN 0385	FTT0856c	FTL 0355	386244	385393	284	ubiA	4-hydroxybenzoate octaprenyltransferase	cofactors, prosthetic groups, electron carriers metabolism	unknown	
FTN 0386	FTT0857c	FTL 0356	386753	386250	168	ubiC	chorismate pyruvate lyase	cofactors, prosthetic groups, electron carriers metabolism	unknown	
FTN 0387	FTT0858	FTL 0357	386852	387556	235	-	ribonuclease PH	translation, ribosomal structure and biogenesis	cytoplasm	
FTN 0388	FTT0859c	FTL 0358	387936	387616	107	-	protein of unknown function	unknown function - novel	unknown	
FTN 0389	(FTT0860c-FTT0861c)	(FTL 0359)	388991	388044	316	-	Type IV pili, pilus assembly protein	motility, attachment and secretion structure	unknown	yes
FTN 0390	FTT0862c	FTL 0360	390163	389099	355	htpX	Zn-dependent protease with chaperone function	post-translational modification, protein turnover, chaperones - chaperones	unknown	
FTN 0391	FTT0863c	FTL 0361	390742	390170	191	-	LemA-like protein	putative enzymes	unknown	
FTN 0392	FTT0864c	(337765-338658)	391710	390793	306	-	transcriptional regulator, LysR family	signal transduction and regulation	unknown	
FTN 0393	(FTT0865)	(338797-339849)	391849	392901	351	-	conserved protein of unknown function	unknown function - conserved	unknown	
FTN 0394	(FTT0866c)	(339857-342873)	395071	392909	721	-	heavy metal cation transport ATPase	transport	unknown	
FTN 0395	FTT0868c	FTL 0370	395433	395071	121	-	transcriptional regulator, ArsR family	signal transduction and regulation	unknown	
FTN 0396	FTT0869	FTL 0371	395576	396226	217	-	conserved protein of unknown function	unknown function - conserved	unknown	
FTN 0397	FTT0871	FTL 0372	396549	397544	332	gpsA	glycerol-3-phosphate-dehydrogenase-[NAD+]	fatty acids and lipids metabolism	unknown	
FTN 0398	FTT0872c	FTL 0373	398084	397551	178	-	hypothetical membrane protein	hypothetical - novel	cytoplasmic membrane	
FTN 0399	FTT0873c	FTL 0374	399459	398092	456	radA	DNA repair protein radA	DNA replication, recombination, modification and repair - repair	unknown	
FTN 0400	FTT0874c	FTL 0375	400237	399470	256	-	protein of unknown function	unknown function - novel	cytoplasm	
FTN 0401	FTT0875c	FTL 0376	400833	400237	199	-	hypothetical membrane protein	hypothetical - novel	cytoplasmic membrane	
FTN 0402	FTT0876c	FTL 0377	401894	400839	352	aroC	chorismate synthase	amino acid metabolism - biosynthesis	unknown	
FTN 0403	FTT0877c	FTL 0378	402688	402038	217	-	hypothetical membrane protein	hypothetical - novel	cytoplasmic membrane	
FTN 0404	FTT0878c	FTL 0379	403219	402710	170	-	peptide methionine sulfoxide reductase-related protein	post-translational modification, protein turnover, chaperones	unknown	
FTN 0405	FTT0879	FTL 0380	403380	403934	185	sodC	superoxide dismutase (Cu-Zn) precusor	other metabolism - degradation, utilization, assimilation	unknown	
FTN 0406	(FTT0880)	FTL 0381	403941	404729	263	-	sterol desaturase	fatty acids and lipids metabolism	unknown	
FTN 0407	FTT0881c	FTL 0382	406101	404710	464	-	amino acid transporter (AAT) family protein	transport - amino-acid	cytoplasmic membrane	
FTN 0408	(FTT0882)	(FTL 0383)	406141	406611	157	-	mannose-6-phosphate isomerase	carbohydrate metabolism - biosynthesis	unknown	yes
FTN 0409	(FTT0883)	(354508-355602)	406706	407815	370	adhC	Zn-dependent alcohol dehydrogenase	energy metabolism	unknown	
FTN 0410	FTT0884c	FTL 0387	409057	407870	396	-	aspartate/tyrosine/aromatic aminotransferase	amino acid metabolism - biosynthesis	unknown	
FTN 0411	(FTT0885)	FTL 0388	409260	410144	295	-	cation diffusion facilitator (CDF) family protein	transport	unknown	
FTN 0412	FTT0886	FTL 0389	410233	411879	549	recN	DNA repair protein	DNA replication, recombination, modification and repair - restriction/modification	cytoplasm	
FTN 0413	(FTT0887c-FTT0888c)	(FTL 0390)	413349	412120	410	pilV	Type IV pili, pilus assembly protein	motility, attachment and secretion structure	unknown	
FTN 0414	FTT0889c	FTL 0391	414019	413585	145	-	Type IV pili, pilus assembly protein	motility, attachment and secretion structure	unknown	
FTN 0415	FTT0890c	(FTL 0392)	414556	414152	135	pilA	Type IV pili, pilus assembly protein	motility, attachment and secretion structure	unknown	
FTN 0416	FTT0891	FTL 0393	414941	415657	239	lpxE	lipid A 1-phosphatase	fatty acids and lipids metabolism	unknown	
FTN 0417	FTT0892	FTL 0394	415800	416645	282	folD	methyleneTHF enzyme/ methenyltetrahydrofolate cyclohydrolase/ methylenetetrahydrofolate dehydrogenase	amino acid metabolism - degradation, utilization, assimilation	unknown	
FTN 0418	Schunoseq	LVSnoseq	416811	417098	96	-	endonuclease	DNA replication, recombination, modification and repair - degradation	unknown	
FTN 0419	FTT0893	FTL 0395	417319	418359	347	purM	phosphoribosylformylglycinamide cyclo-ligase	nucleotides and nucleosides metabolism	unknown	
FTN 0420	FTT0894	FTL 0396	418362	420671	770	-	SAICAR synthetase/phosphoribosylamine-glycine ligase	nucleotides and nucleosides metabolism	unknown	
FTN 0421	FTT0895	FTL 0397	420678	421250	191	purN	phosphoribosylglycinamide formyltransferase	nucleotides and nucleosides metabolism	unknown	
FTN 0422	FTT0896	FTL 0398	421370	421858	163	purE	N5-carboxyaminoimidazole ribonucleotide mutase	nucleotides and nucleosides metabolism	unknown	
FTN 0423	FTT0897	FTL 0399	421865	422959	365	purK	N5-carboxyaminoimidazole ribonucleotide synthase monomer	nucleotides and nucleosides metabolism	unknown	
FTN 0424	FTT0898c	(FTL 0418)	423312	422962	117	-	conserved protein of unknown function	unknown function - conserved	unknown	
FTN 0425	FTT0899c	FTL 0419	425399	423396	668	prlC	oligopeptidase A	amino acid metabolism	unknown	
FTN 0426	FTT0900	FTL 0420	425491	425859	123	-	conserved protein of unknown function	unknown function - conserved	unknown	
FTN 0427	FTT0901	FTL 0421	425944	426390	149	-	lipoprotein of unknown function	unknown function - novel	unknown	
FTN 0428	FTT0902	(389425-389827)	426551	427027	159	-	protein of unknown function	unknown function - novel	unknown	
FTN 0429	FTT0903	FTL 0423	427130	427630	167	-	conserved protein of unknown function	unknown function - conserved	unknown	
FTN 0430	FTT0904	(FTL_0424-FTL 0424)	427666	428130	155	-	protein of unknown function	unknown function - novel	unknown	
FTN 0431	FTT0905	FTL 0425	428206	429645	480	-	hypothetical membrane protein	hypothetical - novel	cytoplasmic membrane	
FTN 0432	FTT0906c	FTL 0426	431996	429705	764	topA	DNA topoisomerase I	DNA replication, recombination, modification and repair - restriction/modification	cytoplasm	
FTN 0433	FTT0907	FTL 0427	432195	432833	213	parA	chromosome partition protein A, ATPase	cell cycle	unknown	
FTN 0434	FTT0908	FTL 0428	432829	433740	304	parB	chromosome partition protein B	cell cycle	unknown	
FTN 0435	FTT0909	FTL 0429	433757	434461	235	-	glutamine amidotransferase, class I	other metabolism - biosynthesis	unknown	
FTN 0436	FTT0910	FTL 0430	434467	435345	293	-	lipolytic enzyme	fatty acids and lipids metabolism	extracellular	
FTN 0437	FTT0911	(FTL_0431-FTL 0432)	435345	435932	196	-	hydrolase, HD superfamily	putative enzymes	unknown	
FTN 0438	FTT0912c	FTL 0433	436615	435998	206	rrmJ	23S rRNA methylase	translation, ribosomal structure and biogenesis	unknown	
FTN 0439	FTT0913	FTL 0434	436773	437237	155	-	protein of unknown function	unknown function - novel	unknown	
FTN 0440	FTT0914c	FTL 0435	437720	437238	161	lspA	lipoprotein signal peptidase II	motility, attachment and secretion structure	unknown	
FTN 0441	FTT0915c	FTL 0436	440514	437710	935	ileS	isoleucyl-tRNA synthetase	other metabolism - biosynthesis	unknown	
FTN 0442	FTT0916c	FTL 0437	441527	440610	306	ribF	riboflavin kinase/FMN adenylyltransferase	cofactors, prosthetic groups, electron carriers metabolism	unknown	
FTN 0443	FTT0917	FTL 0438	441678	443489	604	maeA	NAD-dependent malic enzyme	energy metabolism	unknown	
FTN 0444	FTT0918	FTL_0439(fusioninLVS,butnotholarctica)	443654	445324	557	-	membrane protein of unknown function	unknown function - novel	unknown	
FTN 0445	FTT0919	FTL_0439(fusioninLVS,butnotholarctica)	445344	446786	481	-	protein of unknown function	unknown function - novel	outer membrane	
FTN 0446	FTT1306c	FTL 0414	446993	448387	465	engA	GTP-binding protein	putative enzymes	unknown	

FTN_0447	FTT1305c	FTL_0413	448359	449660	434	murA	UDP-N-acetylglucosamine enolpyruvoyl transferase	cell wall / LPS / capsule	unknown	
FTN_0448	FTT1304c	FTL_0412	449671	450516	282	murB	UDP-N-acetylmuramate dehydrogenase	cell wall / LPS / capsule	unknown	
FTN_0449	FTT1303c	FTL_0411	450598	451470	291	-	conserved protein of unknown function	unknown function - conserved	unknown	
FTN_0450	FTT1302	FTL_0410	452251	451478	258	-	protein of unknown function	unknown function - novel	unknown	
FTN_0451	Schunoseq	LVSnoseq	454379	452277	701	-	signal transduction protein with a PAS, a PAC, an EAL and a GGDEF domain	signal transduction and regulation	unknown	
FTN_0452	Schunoseq	LVSnoseq	454529	455929	467	-	hypothetical protein	hypothetical - novel	unknown	
FTN_0453	Schunoseq	LVSnoseq	455936	457249	438	-	glycosyl transferase	cell wall / LPS / capsule	unknown	
FTN_0454	Schunoseq	LVSnoseq	457266	458279	338	-	conserved protein of unknown function	unknown function - conserved	unknown	
FTN_0455	Schunoseq	LVSnoseq	458374	461271	966	-	CheB methylesterase/CheR methyltransferase	signal transduction and regulation	unknown	
FTN_0456	Schunoseq	LVSnoseq	461255	463570	772	-	signal transduction protein with a PAS, a PAC, an EAL and a GGDEF domain	signal transduction and regulation	unknown	
FTN_0457	(FTT1301c)	FTL_0409	463591	464247	219	-	conserved hypothetical protein	hypothetical - conserved	unknown	
FTN_0458	FTT1299	FTL_0408	465103	464768	112	hitA	histidine triad (HIT) family protein	putative enzymes	unknown	
FTN_0459	FTT1298	FTL_0407	466767	465112	552	ubiB	2-octaprenylphenol hydroxylase	cofactors, prosthetic groups, electron carriers metabolism	unknown	
FTN_0460	FTT1297	FTL_0406	467347	466763	195	-	conserved protein of unknown function	unknown function - conserved	unknown	
FTN_0461	FTT1296	FTL_0405	468101	467352	250	ubiE	ubiquinone/menaquinone biosynthesis methyltransferase	cofactors, prosthetic groups, electron carriers metabolism	unknown	
FTN_0462	FTT1295c	FTL_0404	468195	469208	338	glk	glucose kinase (glucokinase)	carbohydrate metabolism - degradation, utilization, assimilation	unknown	
FTN_0463	1319250-1319513	FTL_0403	469476	469216	87	-	hypothetical protein	hypothetical - novel	unknown	
FTN_0464	FTT1294	FTL_0402	470080	469553	176	-	intracellular septation protein A family protein	cell cycle	unknown	
FTN_0465	FTT1293c	(FTL_0401)	470141	470752	204	-	Sua5/YciO/YrdC family protein	putative enzymes	unknown	
FTN_0466	FTT1292c	FTL_0442	470805	471404	200	-	conserved hypothetical protein	hypothetical - conserved	unknown	
FTN_0467	FTT1291	FTL_0443	472627	471407	407	-	sugar transporter, MFS superfamily	transport - carbohydrates (sugars, polysaccharides)	cytoplasmic membrane	
FTN_0468	FTT1290	FTL_0444	474701	472647	685	metG	methionyl-tRNA synthetase	other metabolism - biosynthesis	unknown	
FTN_0471	(FTT1289)	FTL_0445	475760	475227	178	-	NADPH-dependent FMN reductase	other metabolism - degradation, utilization, assimilation	unknown	
FTN_0477	(FTT1343c)	(420903-FTL_0446)	481348	482232	295	-	conserved protein of unknown function	unknown function - conserved	unknown	
FTN_0478	FTT1342	FTL_0447	484438	483386	351	-	conserved hypothetical membrane protein	hypothetical - conserved	cytoplasmic membrane	
FTN_0479	FTT1341	FTL_0448	485208	484555	218	-	metallopeptidase, M50B family	post-translational modification, protein turnover, chaperones - protein degradation	cytoplasmic membrane	
FTN_0480	FTT0383	FTL_0449	486541	486873	111	-	protein of unknown function	unknown function - novel	unknown	
FTN_0481	FTT0384c	FTL_0450	487700	486852	283	psd	phosphatidylserine decarboxylase, proenzyme	fatty acids and lipids metabolism	unknown	
FTN_0482	FTT0385	FTL_0451	487800	488777	326	-	protein of unknown function	unknown function - novel	unknown	
FTN_0483	FTT0386	FTL_0452	488789	489829	347	-	bifunctional NMN adenylyltransferase/Nudix hydrolase	cofactors, prosthetic groups, electron carriers metabolism	unknown	
FTN_0484	FTT0387	FTL_0453	489846	491210	455	glmU	UDP-N-acetylglucosamine pyrophosphorylase/glucosamine-1-phosphate N-acetyltransferase	other metabolism - degradation, utilization, assimilation	unknown	
FTN_0485	FTT0388	FTL_0454	491234	493069	612	glmS	Glucosamine--fructose-6-phosphate aminotransferase	other metabolism - degradation, utilization, assimilation	unknown	
FTN_0486	FTT0389	FTL_0455	493144	493674	177	-	acetyltransferase	putative enzymes	unknown	
FTN_0487	FTT0390c	FTL_0456	493871	493677	65	-	30S ribosomal protein S21	translation, ribosomal structure and biogenesis	cytoplasm	
FTN_0488	FTT0391c	FTL_0457	494074	493874	67	cspC	cold shock protein, DNA-binding	signal transduction and regulation	unknown	
FTN_0489	FTT0392c	FTL_0458	494654	494328	109	-	conserved protein of unknown function	unknown function - conserved	unknown	
FTN_0490	FTT0393	FTL_0459	494781	495548	256	map	methionine aminopeptidase	post-translational modification, protein turnover, chaperones - protein modification	unknown	
FTN_0491	FTT0394	FTL_0460	495553	496398	282	-	hypothetical protein	hypothetical - novel	unknown	
FTN_0492	FTT0396	FTL_0462	496504	498729	742	parC	DNA topoisomerase IV subunit A	DNA replication, recombination, modification and repair - replication	unknown	
FTN_0493	FTT0397	FTL_0463	498729	499412	228	mtn	5'-methylthioadenosine/S-adenosylhomocysteine nucleosidase	nucleotides and nucleosides metabolism	unknown	
FTN_0494	FTT0398c	FTL_0464	500001	499405	199	-	hypothetical membrane protein	hypothetical - novel	cytoplasmic membrane	
FTN_0495	FTT0399c	FTL_0465	501112	499997	372	-	BNR/Asp-box repeat protein	putative enzymes	unknown	
FTN_0496	FTT0400	FTL_0466	501311	503284	658	slt	soluble lytic murein transglycosylase	cell wall / LPS / capsule	unknown	
FTN_0497	Schunoseq	(FTL_0469)	503723	503304	140	-	hypothetical protein	hypothetical - novel	unknown	
FTN_0498	(FTT0401)	(FTL_0470-FTL_0471)	504268	505494	409	-	hypothetical membrane protein	hypothetical - novel	cytoplasmic membrane	yes
FTN_0499	FTT0402	FTL_0472	505748	509224	1159	dnaE	DNA polymerase III, alpha subunit	DNA replication, recombination, modification and repair - restriction/modification	cytoplasm	
FTN_0500	FTT0403	FTL_0473	509303	509935	211	-	peptide deformylase	translation, ribosomal structure and biogenesis	unknown	
FTN_0502	FTT0404	FTL_0474	510648	511907	420	-	ABC transporter, involved in lipoprotein release, permease component	transport	unknown	
FTN_0503	FTT0405	FTL_0475	511903	512595	231	-	ABC transporter, ATPase component	transport	unknown	
FTN_0504	FTT0406	FTL_0476	512714	514852	713	-	lysine decarboxylase	amino acid metabolism - degradation, utilization, assimilation	unknown	
FTN_0505	FTT0407	FTL_0477	514946	516019	358	gcvT	glycine cleavage complex protein T (aminomethyltransferase)	amino acid metabolism - degradation, utilization, assimilation	unknown	
FTN_0506	FTT0408	FTL_0478	516080	516460	127	gcvH	glycine cleavage system H protein (lipoate-binding)	amino acid metabolism - degradation, utilization, assimilation	unknown	
FTN_0507	FTT0409	FTL_0479	516528	517892	455	gcvP1	glycine cleavage system P protein, subunit 1	amino acid metabolism - degradation, utilization, assimilation	unknown	
FTN_0508	FTT0410	FTL_0480	517901	519343	481	gcvP2	glycine cleavage system P protein, subunit 2	amino acid metabolism - degradation, utilization, assimilation	unknown	
FTN_0509	Schunoseq	LVSnoseq	519765	523154	1130	-	conserved protein of unknown function	unknown function - conserved	outer membrane	
FTN_0510	Schunoseq	LVSnoseq	523321	523683	121	-	hypothetical protein	hypothetical - novel	unknown	
FTN_0511	FTT0411c	FTL_0481	524659	523817	281	-	shikimate 5-dehydrogenase	amino acid metabolism - biosynthesis	unknown	
FTN_0512	FTT0412c	FTL_0482	527897	524688	1070	glgX	pullulanase	carbohydrate metabolism - degradation, utilization, assimilation	unknown	
FTN_0513	FTT0413c	FTL_0483	529853	527934	640	glgB	1,4-alpha-glucan branching enzyme	carbohydrate metabolism - biosynthesis	unknown	
FTN_0514	FTT0414	FTL_0484	530155	531786	544	pgm	phosphoglucomutase	carbohydrate metabolism - degradation, utilization, assimilation	unknown	
FTN_0515	(FTT0415)	FTL_0485	531823	533091	423	glgC	glucose-1-phosphate adenylyltransferase	carbohydrate metabolism - biosynthesis	unknown	
FTN_0516	FTT0416	FTL_0486	533104	534570	489	glgA	glycogen synthase	carbohydrate metabolism - biosynthesis	unknown	
FTN_0517	FTT0417	FTL_0487	534678	536948	757	glgP	glycogen phosphorylase	carbohydrate metabolism - degradation, utilization, assimilation	unknown	
FTN_0518	FTT0418	FTL_0488	536994	538454	487	malQ	4-alpha-glucanotransferase	carbohydrate metabolism - degradation, utilization, assimilation	unknown	
FTN_0519	FTT0419	FTL_0489	538549	539436	296	glyQ	glycyl-tRNA synthetase alpha chain	other metabolism - biosynthesis	unknown	
FTN_0520	FTT0420	FTL_0490	539650	541086	479	murE	UDP-N-acetylmuramoylalanyl-D-glutamate--2,6-diaminopimelate ligase	cell wall / LPS / capsule	unknown	
FTN_0521	(FTT0421)	FTL_0491	541170	541526	119	-	protein of unknown function	unknown function - novel	unknown	
FTN_0522	FTT0422	FTL_0492	541881	543236	452	murF	d-alanyl-d-alanine-adding enzyme	cell wall / LPS / capsule	unknown	
FTN_0523	(FTT0423-FTT0424)	FTL_0493	543248	544063	272	-	protein of unknown function	unknown function - novel	unknown	
FTN_0524	FTT0425c	FTL_0494	545216	544119	366	asd	aspartate semialdehyde dehydrogenase	amino acid metabolism - biosynthesis	unknown	
FTN_0525	(FTT0426)	(481283-482137)	545312	547729	806	thrA	aspartate kinase I/homoserine dehydrogenase I	amino acid metabolism - biosynthesis	unknown	
FTN_0526	(FTT0427)	(482301-482775)	547735	548685	317	thrB	homoserine kinase	amino acid metabolism - biosynthesis	unknown	
FTN_0527	FTT0428	FTL_0498	548701	549987	429	thrC	threonine synthase	amino acid metabolism - biosynthesis	unknown	
FTN_0528	FTT0436c	FTL_0506	550740	550021	240	lpxH	UDP-2,3-diacylglucosamine hydrolase	fatty acids and lipids metabolism	unknown	
FTN_0529	FTT0437c	FTL_0507	551356	550733	208	pyrE	orotate phosphoribosyltransferase	nucleotides and nucleosides metabolism	unknown	
FTN_0530	FTT0438	FTL_0508	551516	552883	456	mpl	UDP-N-acetylmuramate:L-alanyl-gamma-D-glutamyl-meso-diaminopimelate ligase	cell wall / LPS / capsule	unknown	
FTN_0531	FTT0439	FTL_0509	552883	553626	248	yjfH	tRNA/rRNA methyltransferase	translation, ribosomal structure and biogenesis	unknown	
FTN_0532	(FTT0441c)	FTL_1625	554289	553648	214	-	conserved hypothetical membrane protein	hypothetical - conserved	cytoplasmic membrane	
FTN_0533	FTT0442c	FTL_1624	555512	554322	397	-	drug:H+ antiporter-1 (DHA1) family protein	transport - drugs / antibacterial compounds	cytoplasmic membrane	
FTN_0534	FTT0443	FTL_1623	555784	556824	347	-	conserved hypothetical membrane protein	hypothetical - conserved	cytoplasmic membrane	
FTN_0535	FTT0444	FTL_1622	556910	558133	408	-	drug:H+ antiporter-1 (DHA1) family protein	transport - drugs / antibacterial compounds	cytoplasmic membrane	
FTN_0536	(FTT0445)	FTL_1621	558170	560008	613	yjjK	(putative) drug resistance ATPase-1 (Drug RA1) family protein	transport - drugs / antibacterial compounds	unknown	
FTN_0537	FTT0446	(FTL_1620)	560098	561507	470	-	proton-dependent oligopeptide transporter (POT) family protein, di- or tripeptide:H+ symporter	transport	cytoplasmic membrane	
FTN_0538	FTT0447c	(FTL_1618-FTL_1619)	562995	561538	486	-	conserved hypothetical membrane protein	hypothetical - conserved	cytoplasmic membrane	
FTN_0539	FTT0448c	FTL_1617	564662	563019	548	glnS	glutaminyl-tRNA synthetase	other metabolism - biosynthesis	unknown	
FTN_0540	FTT0449	FTL_1616	564807	566396	530	pckA	phosphoenolpyruvate carboxykinase	energy metabolism	unknown	
FTN_0541	FTT0450	FTL_1615	566474	567568	365	mraY	phospho-N-acetylmuramoyl-pentapeptidetransferase	cell wall / LPS / capsule	unknown	
FTN_0542	FTT0451	FTL_1614	567571	568821	417	murD	UDP-N-acetylmuramoylalanine--D-glutamate ligase	cell wall / LPS / capsule	unknown	

FTN_0543	FTT0452	FTL_1613	568834	570036	401	ftsW	cell division protein FtsW	cell cycle	unknown	
FTN_0544	FTT0453c	FTL_1612	570853	570050	268	-	conserved hypothetical protein	hypothetical - conserved	unknown	
FTN_0545	FTT0454	FTL_1611	570952	571905	318	-	glycosyl transferase, group 2	cell wall / LPS / capsule	unknown	
FTN_0546	FTT0455c	FTL_1609	574064	572304	587	-	dolichyl-phosphate-mannose-protein mannosyltransferase family protein	cell wall / LPS / capsule	cytoplasmic membrane	
FTN_0547	FTT0456c	FTL_1608	574419	574159	87	-	protein of unknown function	unknown function - conserved	unknown	
FTN_0548	FTT0457c	FTL_1607	574754	574443	104	-	conserved hypothetical protein	hypothetical - conserved	unknown	
FTN_0549	FTT0458	FTL_1606	574840	575469	210	sspA	stringent starvation protein A	signal transduction and regulation	cytoplasm	
FTN_0550	FTT0459	FTL_1605	575484	576497	338	sohB	peptidase family S49 protein	post-translational modification, protein turnover, chaperones - protein modification	unknown	
FTN_0551	FTT0460	FTL_1604	576508	577416	303	holB	DNA polymerase III, delta prime subunit	DNA replication, recombination, modification and repair - restriction/modification	cytoplasm	
FTN_0552	FTT0461	FTL_1603	577430	577705	92	yhbY	RNA-binding protein	translation, ribosomal structure and biogenesis	unknown	
FTN_0553	FTT0462	FTL_1602	577716	578687	324	hemB	delta-aminolevulinic acid dehydratase	cofactors, prosthetic groups, electron carriers metabolism	unknown	
FTN_0554	FTT0463	FTL_1601	578694	579158	155	-	RNA methyltransferase, SpoU family	translation, ribosomal structure and biogenesis	unknown	
FTN_0555	FTT0464	(FTL_1600)	579267	580328	354	ansB	periplasmic L-asparaginase II precursor	amino acid metabolism - biosynthesis	unknown	
FTN_0556	(FTT0465)	(FTL_1599)	580679	581239	187	-	hypothetical protein	hypothetical - novel	unknown	
FTN_0557	FTT0466c	FTL_1598	583038	581296	581	argS	arginyl-tRNA synthetase	other metabolism - biosynthesis	unknown	
FTN_0558	FTT0467	FTL_1597	583164	585767	868	ostA1	organic solvent tolerance protein, OstA	cell wall / LPS / capsule	unknown	
FTN_0559	FTT0468	FTL_1596	585770	587176	469	-	peptidyl-prolyl cis-trans isomerase (PPIase)	post-translational modification, protein turnover, chaperones	unknown	
FTN_0560	FTT0469	FTL_1595	587181	587966	262	ksgA	dimethyladenosine transferase	transport - amino-acid	unknown	
FTN_0561	FTT0470	FTL_1594	587981	588805	275	apaH	diadenosine tetraphosphatase	signal transduction and regulation	unknown	
FTN_0562	FTT0471	FTL_1593	588812	589246	145	aroD	3-dehydroquinate dehydratase	amino acid metabolism - biosynthesis	unknown	
FTN_0563	FTT0472	FTL_1592	589254	589733	160	accB	acetyl-CoA carboxylase, biotin carboxy carrier protein subunit	fatty acids and lipids metabolism	unknown	
FTN_0564	FTT0473	FTL_1591	589801	591153	451	accC	acetyl-CoA carboxylase, biotin carboxylase subunit	fatty acids and lipids metabolism	unknown	
FTN_0565	FTT0474	FTL_1590	591250	591927	226	-	conserved protein of unknown function	unknown function - conserved	unknown	
FTN_0566	FTT0475	(FTL_1588)	591956	593071	372	-	mechanosensitive ion channel protein	transport	cytoplasmic membrane	
FTN_0567	FTT0476c	FTL_1587	594002	593082	307	-	tRNA synthetase class II (D, K and N)	other metabolism - biosynthesis	unknown	
FTN_0568	FTT0477c	FTL_1586	594781	594002	260	-	birA-like protein	post-translational modification, protein turnover, chaperones - protein modification	unknown	
FTN_0569	FTT0478c	FTL_1585	596520	594781	580	recJ	single-stranded-DNA-specific exonuclease	DNA replication, recombination, modification and repair - restriction/modification	cytoplasm	
FTN_0570	FTT0479c	FTL_1584	597620	596517	368	perM	PerM family protein	transport	unknown	
FTN_0571	FTT0480c	FTL_1583	599131	597725	469	-	amino acid-polyamine-organocation (APC) superfamily protein	transport - amino-acid	unknown	
FTN_0572	FTT0481	FTL_1582	599215	600393	393	potF	ATP-binding cassette putrescine uptake system, periplasmic protein	transport	unknown	
FTN_0573	FTT0482c	FTL_1581	601452	600640	271	-	protein of unknown function	unknown function - novel	unknown	
FTN_0574	FTT0483c	FTL_1580	602558	601674	295	-	GTPase of unknown function	putative enzymes	unknown	
FTN_0575	FTT0484	FTL_1579	602712	603428	239	-	protein of unknown function	unknown function - novel	unknown	
FTN_0576	FTT0485	(FTL_1577-FTL_1578)	603499	604158	220	-	conserved protein of unknown function	unknown function - conserved	unknown	
FTN_0577	FTT0486	FTL_1576	604169	605968	600	mutL	DNA mismatch repair enzyme with ATPase activity	DNA replication, recombination, modification and repair - restriction/modification	cytoplasm	
FTN_0578	(FTT0487)	(FTL_1575)	606155	607375	407	-	major facilitator superfamily (MFS) transport protein	transport	cytoplasmic membrane	yes
FTN_0579	FTT0488c	(FTL_1573-FTL_1574)	608659	607388	424	-	major facilitator superfamily (MFS) transport protein	transport	cytoplasmic membrane	
FTN_0580	FTT0489c	FTL_1571	609717	608770	316	trxB	thioredoxin reductase	cofactors, prosthetic groups, electron carriers metabolism	unknown	
FTN_0581	FTT0490c	FTL_1570	611025	609811	405	-	phospholipase D family protein	fatty acids and lipids metabolism	unknown	
FTN_0582	FTT0491c	FTL_1569	611742	611071	224	gph	phosphoglycolate phosphatase	putative enzymes	unknown	
FTN_0583	FTT0492c	FTL_1568	612709	611786	308	-	transcriptional regulator, LysR family	signal transduction and regulation	unknown	
FTN_0584	(FTT0493)	FTL_1567	612820	613983	388	araJ	conserved inner membrane protein of unknown function	unknown function - conserved	cytoplasmic membrane	
FTN_0585	FTT0494c	FTL_1566	614723	614004	240	cutC	copper homeostasis protein CutC family protein	cofactors, prosthetic groups, electron carriers metabolism	unknown	
FTN_0586	FTT0495	(1493398-1493898)	615044	615559	172	-	conserved hypothetical protein	hypothetical - conserved	unknown	
FTN_0587	Schunoseq	LVSnoseq	615588	616814	409	-	deoxyguanosinetriphosphate triphosphohydrolase	nucleotides and nucleosides metabolism	unknown	
FTN_0588	(FTT0497c)	(1491700-1492618)	617740	616823	306	-	asparaginase	amino acid metabolism - degradation, utilization, assimilation	unknown	
FTN_0589	(FTT0498c)	(1490252-1491693)	619189	617747	481	-	proton-dependent oligopeptide transporter (POT) family protein, di- or tripeptide:H+ symporter	transport	cytoplasmic membrane	
FTN_0590	(FTT0499-FTT0500)	(FTL_1557-FTL_1558)	620283	621500	406	-	conserved protein of unknown function	unknown function - conserved	unknown	
FTN_0591	FTT0501c	FTL_1556	622667	621630	346	-	conserved hypothetical membrane protein	hypothetical - conserved	cytoplasmic membrane	
FTN_0592	FTT0502c	FTL_1555	623476	623054	141	-	hypothetical membrane protein	hypothetical - novel	cytoplasmic membrane	
FTN_0593	FTT0503c	FTL_1554	624460	623591	290	sucD	succinyl-CoA synthetase, alpha subunit	energy metabolism	unknown	
FTN_0594	FTT0504c	FTL_1553	625655	624495	387	sucC	succinyl-CoA synthetase, beta chain	energy metabolism	unknown	
FTN_0595	FTT0505	FTL_1552	625825	627738	638	-	outer membrane protein of unknown function	unknown function - novel	outer membrane	
FTN_0596	(FTT0506c)	(FTL_1551)	628633	627752	294	-	hypothetical protein	hypothetical - novel	unknown	yes
FTN_0597	FTT0507	FTL_1550	628731	629495	255	-	protein-disulfide isomerase	post-translational modification, protein turnover, chaperones - protein modification	unknown	
FTN_0598	FTT0508c	FTL_1549	630488	629508	327	-	tRNA-dihydrouridine synthase	translation, ribosomal structure and biogenesis	unknown	
FTN_0599	FTT0509c	FTL_1548	631322	630525	266	-	protein of unknown function	unknown function - novel	unknown	
FTN_0600	FTT0510	FTL_1547	631504	633912	803	gyrB	DNA gyrase subunit B	DNA replication, recombination, modification and repair - restriction/modification	cytoplasm	
FTN_0601	FTT0511	FTL_1546	634045	634905	287	-	pyridoxine/pyridoxal 5-phosphate biosynthesis protein	cofactors, prosthetic groups, electron carriers metabolism	unknown	
FTN_0602	FTT0512	FTL_1545	634911	635447	179	-	glutamine amidotransferase, SNO family	cofactors, prosthetic groups, electron carriers metabolism	unknown	
FTN_0603	FTT0693c	FTL_1543	636283	635462	274	mutM	formamidopyrimidine-DNA glycosylase	DNA replication, recombination, modification and repair - restriction/modification	cytoplasm	
FTN_0604	FTT0694	FTL_1542	636377	638473	699	-	AMP-binding protein	putative enzymes	unknown	
FTN_0605	FTT0695	FTL_1541	638482	639396	305	mraW	S-adenosylmethionine-dependent methyltransferase	cell wall / LPS / capsule	unknown	
FTN_0606	FTT0696	FTL_1540	639396	639743	116	-	hypothetical protein	hypothetical - novel	unknown	
FTN_0607	FTT0697	FTL_1539	639739	641430	564	ftsI	cell division protein, peptidoglycan synthetase (PBP)	cell cycle	unknown	
FTN_0608	FTT0698	FTL_1538	641539	641802	88	rpsO	30S ribosomal protein S15	translation, ribosomal structure and biogenesis	unknown	
FTN_0609	FTT0699	FTL_1537	641862	643940	693	pnp	polyribonucleotide nucleotidyltransferase	translation, ribosomal structure and biogenesis	unknown	
FTN_0610	FTT0700	FTL_1536	643949	644296	116	-	conserved protein of unknown function	unknown function - conserved	unknown	
FTN_0611	FTT0701	FTL_1535	644330	645154	275	kdsA	3-deoxy-D-manno-octulosonic acid 8-phosphate synthase	fatty acids and lipids metabolism	unknown	
FTN_0612	FTT0702	FTL_1534	645168	645830	221	udk	uridine kinase	nucleotides and nucleosides metabolism	unknown	
FTN_0613	FTT0703	FTL_1533	645826	646041	72	rpoZ	DNA-directed RNA polymerase, subunit K/omega	transcription	cytoplasm	
FTN_0615	FTT0704	FTL_1532	646311	646880	190	-	protein of unknown function	unknown function - novel	unknown	
FTN_0616	FTT0705	FTL_1531	646972	648318	449	-	RNA methyltransferase, trmA family	translation, ribosomal structure and biogenesis	unknown	
FTN_0617	(FTT0706)	LVSnoseq	648331	649293	321	-	ROK family protein	putative enzymes	unknown	
FTN_0618	Schunoseq	(1460405-1461037)	649296	650240	315	-	ROK family protein	putative enzymes	unknown	
(FTN_0619)	FTT0707	FTL_1529	650233	651024						
FTN_0620	FTT0708	FTL_1528	651130	652365	412	-	major facilitator superfamily (MFS) transport protein	transport	cytoplasmic membrane	
FTN_0621	FTT0709	FTL_1527	652477	653844	456	eno	enolase (2-phosphoglycerate dehydratase)	carbohydrate metabolism - degradation, utilization, assimilation	unknown	
FTN_0622	FTT0710	FTL_1526	653855	654142	96	ftsB	cell division protein, septum formation initiator	cell cycle	unknown	
FTN_0623	FTT0711	FTL_1525	654135	654821	229	ispD	2-C-methyl-D-erythritol 4-phosphate cytidylyltransferase	fatty acids and lipids metabolism	unknown	
FTN_0624	FTT0712c	FTL_1524	656091	654832	420	-	serine permease	transport - amino-acid	cytoplasmic membrane	
FTN_0625	FTT0713c	FTL_1523	657148	656096	351	tdh	L-threonine 3-dehydrogenase	amino acid metabolism - degradation, utilization, assimilation	unknown	
FTN_0626	FTT0714c	FTL_1522	658367	657174	398	kbl	2-amino-3-ketobutyrate coenzyme A ligase	amino acid metabolism - degradation, utilization, assimilation	unknown	
FTN_0627	FTT0715	FTL_1521	658901	661510	870	chiA	chitinase, glycosyl hydrolase family 18	carbohydrate metabolism - degradation, utilization, assimilation	unknown	
FTN_0628	FTT0716	FTL_1520	661707	662333	209	upp	uracil phosphoribosyltransferase	nucleotides and nucleosides metabolism	unknown	
FTN_0629	(FTT0717)	(1447622-1448451)	662421	663251	277	purU	formyltetrahydrofolate deformylase	nucleotides and nucleosides metabolism	unknown	yes
FTN_0630	FTT0718	FTL_1517	663345	663686	114	-	hypothetical protein	hypothetical - conserved	unknown	
FTN_0631	FTT0719	FTL_1502	663750	664967	406	-	metabolite:H+ symporter (MHS) family protein	transport	cytoplasmic membrane	

FTN 0632	FTT0720c	FTL 1503	666279	664957	441	-	dGTP triphosphohydrolase	nucleotides and nucleosides metabolism	unknown	
FTN 0633	FTT0721c	FTL 1504	668602	666386	739	katG	peroxidase/catalase	other metabolism - degradation, utilization, assimilation	unknown	
FTN 0634	FTT0723c	(FTL_1506-FTL 1507)	669595	668810	262	-	oxidoreductase, short chain dehydrogenase/reductase family	other metabolism - degradation, utilization, assimilation	unknown	
FTN 0635	(FTT0724c)	(1439291-FTL 1509)	670983	669592	464	-	serine-type D-Ala-D-Ala carboxypeptidase	cell wall / LPS / capsule	unknown	
FTN 0636	FTT0725c	FTL 1510	672315	671002	438	glpT	glycerol-3-phosphate transporter	transport	cytoplasmic membrane	
FTN 0637	FTT0726c	FTL 1511	674314	673289	342	ugpQ	glycerophosphoryl diester phosphodiesterase	cell wall / LPS / capsule	unknown	
FTN 0638	FTT1339c	FTL 1499	674546	676087	514	-	sulfate permease family protein	transport	cytoplasmic membrane	
FTN 0639	FTT1338c	FTL 1498	676090	676467	126	-	endoribonuclease L-PSP	translation, ribosomal structure and biogenesis	unknown	
FTN 0640	FTT1337c	FTL 1497	676608	677855	416	dctA	C4-dicarboxylate transport protein	transport	cytoplasmic membrane	
FTN 0641	FTT1336	FTL 1496	679509	677863	549	cydC	ABC-type transport ATP-binding protein CydC	transport	unknown	
FTN 0642	FTT1335	FTL 1495	681287	679509	593	cydD	ABC-type transport ATP-binding protein CydD	transport	unknown	
FTN 0643	FTT1334c	FTL 1494	681664	682170	169	-	protein of unknown function	unknown function - novel	unknown	
FTN 0644	FTT1333c	1422886-1423083	682201	682398	66	-	protein of unknown function	unknown function - novel	unknown	
FTN 0645	FTT1332	FTL 1493	683664	682408	419	-	multidrug/oligosaccharidyl-lipid/polysaccharide (MOP) transporter	transport - drugs / antibacterial compounds	cytoplasmic membrane	
FTN 0646	FTT1331	FTL 1492	684641	683745	299	cscK	ROK family protein	putative enzymes	unknown	
FTN 0647	FTT1330	FTL 1491	686031	684754	426	serS	seryl-tRNA synthetase	other metabolism - biosynthesis	unknown	
FTN 0648	FTT1329	FTL 1490	687584	686049	512	gpmI	phosphoglycerate mutase, cofactor independent	carbohydrate metabolism - degradation, utilization, assimilation	unknown	
FTN 0649	(FTT1328c)	FTL 1489	687803	690832	1010	-	4Fe-4S ferredoxin, FAD dependent	energy metabolism	unknown	
FTN 0651	FTT1327	FTL 1488	691433	691035	133	cdd	cytidine deaminase	other metabolism - degradation, utilization, assimilation	unknown	
FTN 0652	FTT1326	FTL 1487	692257	691457	267	udp	uridine phosphorylase	other metabolism - degradation, utilization, assimilation	unknown	
FTN 0653	FTT1325c	FTL 1486	692373	692981	203	miaE	tRNA-(ms(2)io(6)a)-hydroxylase	translation, ribosomal structure and biogenesis	unknown	
FTN 0654	FTT1324	FTL 1485	693686	693039	216	-	conserved hypothetical membrane protein	hypothetical - conserved	cytoplasmic membrane	
FTN 0655	FTT1323	FTL 1484	694298	693723	192	-	N6-adenine-specific methylase	DNA replication, recombination, modification and repair	unknown	
FTN 0656	FTT1322	FTL 1483	695522	694302	407	-	Zn-dependent peptidase, M16 family	post-translational modification, protein turnover, chaperones - protein modification	unknown	
FTN 0657	FTT1321	FTL 1482	696782	695532	417	-	metallopeptidase, M16 family	post-translational modification, protein turnover, chaperones - protein modification	unknown	
FTN 0658	FTT1320	FTL 1481	697851	696793	353	-	conserved membrane protein of unknown function	unknown function - conserved	unknown	
FTN 0659	FTT1319	FTL 1480	698933	697854	360	-	conserved membrane protein of unknown function	unknown function - conserved	cytoplasmic membrane	
FTN 0660	FTT1318c	FTL 1479	699099	700535	479	pepA	cytosol aminopeptidase	amino acid metabolism	unknown	
FTN 0661	FTT1317c	FTL 1478	700640	702097	486	guaB	IMP dehydrogenase/GMP reductase	nucleotides and nucleosides metabolism	unknown	
FTN 0662	FTT1316c	FTL 1477	702166	702816	217	-	predicted thiamine pyrophosphokinase	cofactors, prosthetic groups, electron carriers metabolism	unknown	
FTN 0663	FTT1315c	FTL 1476	702806	704425	540	pgi	glucose-6-phosphate isomerase	carbohydrate metabolism - degradation, utilization, assimilation	unknown	
FTN 0664	FTT1314c	FTL 1475	704429	705010	194	fimT	Type IV pili, pilus assembly protein	motility, attachment and secretion structure	unknown	
FTN 0665	FTT1313c	FTL 1474	705047	705526	160	greA	transcriptional elongation factor	transcription	unknown	
FTN 0666	FTT1312c	FTL 1473	705568	708387	940	uvrA	excinuclease ABC, subunit A	DNA replication, recombination, modification and repair - restriction/modification	cytoplasm	
FTN 0667	FTT1311	(FTL_1469-FTL 1472)	709784	708399	462	-	drug:H+ antiporter-1 (DHA2) family protein	transport - drugs / antibacterial compounds	cytoplasmic membrane	
FTN 0668	FTT1310c	FTL 1468	709997	711940	648	hflB	ATP-dependent metalloprotease	cell cycle	unknown	
FTN 0669	FTT0766	FTL 1461	712184	712903	240	deoD	purine nucleoside phosphorylase	nucleotides and nucleosides metabolism	unknown	
FTN 0670	FTT0767c	FTL 1460	713352	712906	149	-	protein of unknown function	unknown function - novel	unknown	
FTN 0671	FTT0768c	FTL 1459	714384	713359	342	-	hypothetical protein	hypothetical - novel	unknown	
FTN 0672	FTT0769	FTL 1458	714516	717233	906	secA	preprotein translocase, subunit A (ATPase, RNA helicase)	motility, attachment and secretion structure	unknown	
FTN 0673	(FTT0770)	(1380870-1381433)	717345	717908	188	tag	3-methyladenine DNA glycosylase	DNA replication, recombination, modification and repair - repair	unknown	yes
FTN 0674	(FTT0771c)	(1379751-1380872)	719030	717906	375	glxK	glycerate kinase	other metabolism - degradation, utilization, assimilation	unknown	
FTN 0675	FTT0772	FTL 1453	719301	719612	104	rplU	ribosomal protein L21	translation, ribosomal structure and biogenesis	unknown	
FTN 0676	FTT0773	FTL 1452	719646	719897	84	rpmA	ribosomal protein L27	translation, ribosomal structure and biogenesis	unknown	
FTN 0677	FTT0774	FTL 1451	721006	721380	125	-	conserved protein of unknown function	unknown function - conserved	unknown	
FTN 0678	(FTT0775c)	(FTL 1450)	722594	721383	404	-	drug:H+ antiporter-1 (DHA1) family protein	transport - drugs / antibacterial compounds	cytoplasmic membrane	
FTN 0679	FTT0776c	FTL 1449	723717	722626	364	rnd	ribonuclease D	translation, ribosomal structure and biogenesis	unknown	
FTN 0680	FTT0777	FTL 1448	723838	725673	612	uvrC	excinuclease ABC, subunit C	DNA replication, recombination, modification and repair - restriction/modification	cytoplasm	
FTN 0681	(FTT0778-FTT1480c)	(FTL 1447)	725743	726366	208	-	acid phosphatase/phosphotransferase	putative enzymes	unknown	yes
FTN 0682	FTT1479c	FTL 1400	726759	726944	62	-	conserved protein of unknown function	unknown function - conserved	unknown	
FTN 0683	FTT1478c	FTL 1399	726940	727689	250	kdsB	3-deoxy-D-manno-octulosonate cytidyltransferase	fatty acids and lipids metabolism	unknown	
FTN 0684	FTT1477c	FTL 1398	727685	728077	131	-	HIT family protein	putative enzymes	unknown	
FTN 0685	FTT1476	FTL 1397	729225	728080	382	galK	galactokinase	carbohydrate metabolism - degradation, utilization, assimilation	unknown	
FTN 0686	FTT1475	FTL 1396	730253	729231	341	galT	galactose-1-phosphate uridylyltransferase	carbohydrate metabolism - degradation, utilization, assimilation	unknown	
FTN 0687	FTT1474c	FTL 1395	730344	731723	460	galP1	galactose-proton symporter, major facilitator superfamily (MFS) transport protein	transport - carbohydrates (sugars, polysaccharides)	cytoplasmic membrane	
FTN 0688	FTT1473c	FTL 1394	731758	733149	464	galP2	galactose-proton symporter, major facilitator superfamily (MFS) transport protein	transport - carbohydrates (sugars, polysaccharides)	cytoplasmic membrane	
FTN 0689	FTT1472c	FTL 1393	733247	733522	92	ppiC	parvulin-like peptidyl-prolyl isomerase domain	post-translational modification, protein turnover, chaperones - protein modification	unknown	
FTN 0690	FTT1471c	FTL 1392	733590	735296	569	deaD	DEAD-box subfamily ATP-dependent helicase	DNA replication, recombination, modification and repair - restriction/modification	unknown	
FTN 0691	FTT1470c	FTL 1391	735416	735985	190	gmk	guanylate kinase	nucleotides and nucleosides metabolism	unknown	
FTN 0692	FTT1469c	FTL 1390	736009	737031	341	nadA	quinolinate sythetase A	cofactors, prosthetic groups, electron carriers metabolism	unknown	
FTN 0693	FTT1468c	FTL 1389	737043	737903	287	nadC	nicotinate-nucleotide pyrophosphorylase	cofactors, prosthetic groups, electron carriers metabolism	unknown	
FTN 0694	FTT1467c	FTL 1388	737909	739393	495	nadB	L-aspartate oxidase	cofactors, prosthetic groups, electron carriers metabolism	unknown	
FTN 0695	(FTT1466c)	(FTL_0401-1317505)	739415	740452	346	add	deoxyadenosine deaminase/adenosine deaminase	nucleotides and nucleosides metabolism	unknown	yes
FTN 0696	(FTT0731c)	(FTL 1385)	741076	740453	208	-	hypothetical membrane protein	hypothetical - novel	cytoplasmic membrane	yes
FTN 0697	FTT0732	FTL 1384	741230	741517	96	-	conserved protein of unknown function	unknown function - conserved	unknown	
FTN 0698	FTT0733	FTL 1383	741553	742020	156	-	glutathione peroxidase	post-translational modification, protein turnover, chaperones - protein modification	unknown	
FTN 0699	(FTT0734)	(1312838-1314003)	742065	743228	388	-	conserved hypothetical protein	hypothetical - conserved	cytoplasmic membrane	yes
FTN 0700	(FTT0735)	(1311864-1312838)	743228	744295	356	-	predicted enzyme of enolase superfamily	putative enzymes	unknown	yes
FTN 0701	FTT0736	FTL 1377	744311	744955	215	-	conserved hypothetical protein	hypothetical - conserved	unknown	
745097-744972	FTT0737c	1310969-1311094	745097	744972						
FTN 0702	FTT0738	FTL 1376	745266	746765	500	-	YjeF-related protein of unknown function	unknown function - conserved	unknown	
FTN 0703	Schunoseq	LVSnoseq	746848	747417	190	-	type I restriction-modification system, subunit S	DNA replication, recombination, modification and repair - restriction/modification	unknown	
FTN 0704	Schunoseq	LVSnoseq	747676	749160	495	-	type I restriction-modification system, subunit M (methyltransferase)	DNA replication, recombination, modification and repair - restriction/modification	unknown	
FTN 0705	Schunoseq	LVSnoseq	749168	750076	303	-	abortive infection bacteriophage resistance protein	mobile and extrachromosomal element functions - phage or plasmid related proteins	unknown	
FTN 0706	Schunoseq	LVSnoseq	750173	750673	167	-	hypothetical membrane protein	hypothetical - novel	cytoplasmic membrane	
FTN 0707	Schunoseq	LVSnoseq	750859	752076	406	-	type I restriction-modification system, subunit S	DNA replication, recombination, modification and repair - restriction/modification	unknown	
FTN 0708	Schunoseq	LVSnoseq	752105	752347	81	-	hypothetical protein	hypothetical - novel	unknown	
FTN 0709	Schunoseq	LVSnoseq	752359	753102	248	-	hypothetical protein	hypothetical - novel	unknown	
FTN 0710	(FTT1642c)	(FTL 1712)	753221	756328	1036	-	type I restriction-modification system, subunit R (restriction)	DNA replication, recombination, modification and repair - restriction/modification	unknown	
FTN 0711	Schunoseq	LVSnoseq	756324	756980	219	-	predicted metal-dependent hydrolase	putative enzymes	unknown	
FTN 0712	FTT0739c	FTL 1375	757410	757045	122	hslR	heat shock protein 15 (HSP15)	translation, ribosomal structure and biogenesis	unknown	

FTN_0713	(FTT0740c-FTT0741c)	(FTL_1373-FTL_1374)	760250	757653	866	ostA2	organic solvent tolerance protein OstA	cell wall / LPS / capsule	unknown	
FTN_0714	(FTT0742)	(FTL_1372)	760571	766126	1852	-	protein of unknown function	unknown function - novel	outer membrane	yes
FTN_0715	(763934-765245)	(FTL_1371)	766940	770707	1256	-	protein of unknown function	unknown function - novel	outer membrane	
FTN_0716	FTT0743	FTL_1370	771325	772137	271	-	protein of unknown function	unknown function - novel	unknown	
FTN_0717	(FTT0744c-FTT0745c-FTT0746c)	(FTL_1367-FTL_1368)	773937	772243	565	-	conserved hypothetical membrane protein	hypothetical - conserved	cytoplasmic membrane	
FTN_0718	FTT0747c	(FTL_1365-FTL_1366)	774791	773937	285	-	membrane fusion protein	motility, attachment and secretion structure	periplasm	
FTN_0719	769938-770141	1298202-1298399	774996	774796	67	-	hypothetical protein	hypothetical - novel	unknown	
FTN_0720	FTT0748	FTL_1364	775195	775908	238	-	transcriptional regulator, IclR family	signal transduction and regulation	unknown	
FTN_0721	FTT0749c	FTL_1363	777051	776068	328	-	conserved hypothetical protein	hypothetical - conserved	unknown	
FTN_0722	FTT0750	FTL_1362	777263	778246	328	-	aminomutase	amino acid metabolism	unknown	
FTN_0723	FTT0751c	FTL_1361	778522	778322	67	cspA	cold shock protein, DNA-binding	transcription	unknown	
FTN_0724	(FTT0752c)	(1294269-1294594)	779050	778724	109	-	hypothetical protein	hypothetical - novel	unknown	
FTN_0725	FTT0753	FTL_1360	779296	779493	66	-	30S ribosomal protein S21	translation, ribosomal structure and biogenesis	cytoplasm	
FTN_0727	FTT0755	FTL_1359	780006	780572	189	-	hypothetical membrane protein	hypothetical - novel	cytoplasmic membrane	
FTN_0728	FTT0756	FTL_1358	780666	781802	379	-	predicted Co/Zn/Cd cation transporter	transport	unknown	
FTN_0729	FTT0757	FTL_1357	781822	782682	287	galU	UTP--glucose-1-phosphate uridylyltransferase	carbohydrate metabolism - degradation, utilization, assimilation	unknown	
FTN_0730	(FTT0758)	(1288818-1290154)	782807	784741	645	acs	acyl-coenzyme A synthetase/AMP-(fatty) acid ligases	other metabolism - degradation, utilization, assimilation	unknown	yes
FTN_0731	FTT0759	FTL_1354	784756	785670	305	-	conserved hypothetical membrane protein	hypothetical - conserved	cytoplasmic membrane	
FTN_0732	FTT0760c	(1287676-1287883)	785882	785679	68	-	hypothetical protein	hypothetical - novel	unknown	
FTN_0733	FTT0761c	FTL_1353	786260	785970	97	-	hypothetical protein	hypothetical - novel	unknown	
FTN_0734	FTT0762c	FTL_1352	787331	786285	349	recF	RecFOR complex, RecF component	DNA replication, recombination, modification and repair - restriction/modification	cytoplasm	
FTN_0735	FTT0763c	(FTL_1351)	788081	787338	248	-	Mg-dependent DNase	DNA replication, recombination, modification and repair - degradation	unknown	
FTN_0736	FTT0764	FTL_1350	788274	790355	694	glyS	glycyl-tRNA synthetase beta subunit	other metabolism - biosynthesis	unknown	
FTN_0737	FTT0564	FTL_0679	791376	790573	268	potI	ATP-binding cassette putrescine uptake system, membrane protein, subunit I	transport	unknown	
FTN_0738	FTT0563	FTL_0680	792307	791393	305	potH	ATP-binding cassette putrescine uptake system, membrane protein, subunit H	transport	unknown	
FTN_0739	FTT0562	FTL_0681	793392	792274	373	potG	ATP-binding cassette putrescine uptake system, ATP-binding protein	transport	unknown	
FTN_0740	FTT0566	(FTL_1347)	793798	794055	86	-	protein of unknown function	unknown function - novel	unknown	
FTN_0741	(FTT0567c)	(1279566-1281013)	795628	794174	485	-	proton-dependent oligopeptide transporter (POT) family protein, di- or tripeptide:H+ symporter	transport	cytoplasmic membrane	
FTN_0742	FTT0568	(FTL_1343-FTL_1344)	796240	796887	216	serB	phosphoserine phosphatase	amino acid metabolism - biosynthesis	unknown	
FTN_0743	FTT0569c	FTL_1342	797901	796882	340	-	conserved hypothetical membrane protein	hypothetical - conserved	cytoplasmic membrane	
FTN_0744	FTT0570	FTL_1341	797981	798376	132	-	protein of unknown function	unknown function - novel	cytoplasm	
FTN_0745	FTT0571	FTL_1340	798391	799095	235	-	protein of unknown function	unknown function - novel	unknown	
FTN_0746	FTT0573	FTL_1338	799235	800329	365	alr	alanine racemase	amino acid metabolism - degradation, utilization, assimilation	unknown	
FTN_0747	(FTT0574)	(1271712-1273287)	800413	802020	536	-	amino acid-polyamine-organocation (APC) superfamily protein	transport - amino-acid	cytoplasmic membrane	
FTN_0748	FTT0575	FTL_1336	802109	802948	280	pheA	prephenate dehydratase	amino acid metabolism - biosynthesis	unknown	
FTN_0749	FTT0576	FTL_1335	802977	803642	222	-	predicted metal-dependent hydrolase	putative enzymes	unknown	
FTN_0750	FTT0577	FTL_1334	803701	805071	457	sdaA	L-serine dehydratase	amino acid metabolism - degradation, utilization, assimilation	unknown	
FTN_0751	FTT0578	FTL_1333	805177	806397	407	sufS	selenocysteine lyase	amino acid metabolism	unknown	
FTN_0752	FTT0579	FTL_1332	806421	806774	118	-	conserved protein of unknown function	unknown function - conserved	unknown	
FTN_0753	FTT0580	FTL_1331	806791	807339	183	-	conserved protein of unknown function	unknown function - conserved	unknown	
FTN_0754	FTT0581	FTL_1330	807354	807839	162	coaD	phosphopantetheine adenylyltransferase	cofactors, prosthetic groups, electron carriers metabolism	unknown	
FTN_0755	FTT0582	FTL_1329	807847	808089	81	-	4Fe-4S ferredoxin	energy metabolism	cytoplasm	
FTN_0756	FTT0583	FTL_1328	808202	809377	392	fopA	OmpA family protein	cell wall / LPS / capsule	unknown	
FTN_0757	(FTT0584)	(1258764-FTL_1327)	810052	814938	1629	-	membrane protein of unknown function	unknown function - novel	outer membrane	
FTN_0758	(FTT0585)	(1257828-1258780)	814922	815875	318	-	conserved hypothetical protein	hypothetical - conserved	unknown	yes
FTN_0759	(605628-605922)	FTL_1321	816036	816326	97	-	conserved hypothetical protein	hypothetical - conserved	unknown	
FTN_0760	FTT0586	FTL_1320	816310	816897	196	-	conserved hypothetical protein	hypothetical - conserved	unknown	
FTN_0761	Schunoseq	LVSnoseq	818940	820022	361	-	protein of unknown function with radical SAM domain	unknown function - conserved	unknown	
FTN_0762	Schunoseq	LVSnoseq	820018	820263	82	grxC	glutaredoxin like protein	post-translational modification, protein turnover, chaperones	unknown	
FTN_0763	Schunoseq	LVSnoseq	820263	820709	149	-	protein of unknown function, thioesterase superfamily	unknown function - conserved	unknown	
FTN_0764	FTT1110	FTL_1088	821078	820728	117	-	hypothetical protein	hypothetical - novel	unknown	
FTN_0765	FTT1109	(FTL_1089)	822211	821246	322	-	choloylglycine hydrolase family protein	cell wall / LPS / capsule	unknown	
FTN_0766	FTT1108	FTL_1090	823058	822309	250	spoU	rRNA methyltransferase	translation, ribosomal structure and biogenesis	unknown	
FTN_0767	(FTT1107c)	(1039160-1041033)	823200	825149	650	betT	betaine/carnitine/choline transporter (BCCT) family protein	transport	cytoplasmic membrane	
FTN_0768	FTT1106	LVSnoseq	825621	825148	158	tspO	tryptophan-rich sensory protein	signal transduction and regulation	unknown	
FTN_0769	FTT1105c	FTL_1093	825750	826601	284	-	peptide methionine sulfoxide reductase	post-translational modification, protein turnover, chaperones - protein modification	unknown	
FTN_0770	(FTT1104)	(1042390-1043548)	827803	826604	400	-	drug:H+ antiporter-1 (DHA1) family protein	transport - drugs / antibacterial compounds	cytoplasmic membrane	yes
FTN_0771	FTT1103	FTL_1096	829204	828086	373	-	protein-disulfide isomerase	post-translational modification, protein turnover, chaperones - protein modification	unknown	
FTN_0772	(FTT1102)	FTL_1097	829515	829228	96	-	conserved protein of unknown function	unknown function - conserved	unknown	
FTN_0773	(FTT1101)	(1045338-1046421)	830691	829606	362	-	4Fe-4S ferredoxin	energy metabolism	unknown	yes
FTN_0774	FTT1100	FTL_1100	831029	830697	111	-	conserved protein of unknown function	unknown function - conserved	cytoplasm	
FTN_0775	FTT1099c	(FTL_1101-FTL_1101)	831132	831464	111	-	hypothetical protein	hypothetical - novel	unknown	
FTN_0776	(FTT1098c)	(1047224-1049106)	831494	833557	688	-	DNA and RNA helicases Superfamily I protein	DNA replication, recombination, modification and repair - replication	unknown	
FTN_0777	FTT1097	FTL_1105	834003	833560	148	-	protein of unknown function	unknown function - novel	unknown	
FTN_0778	FTT1096c	FTL_1106	834159	836753	865	alaS	alanyl-tRNA synthetase	other metabolism - biosynthesis	unknown	
FTN_0779	FTT1095c	FTL_1107	836764	838143	460	-	conserved hypothetical protein	hypothetical - conserved	unknown	
FTN_0780	FTT1094c	FTL_1108	838149	839525	459	pepB	cytosol aminopeptidase	post-translational modification, protein turnover, chaperones - protein degradation	unknown	
FTN_0781	FTT1093c	FTL_1109	839608	840624	339	talA	transaldolase	energy metabolism	unknown	
FTN_0782	(FTT1092c)	(FTL_1110)	840650	841222	191	-	protein of unknown function	unknown function - novel	unknown	
FTN_0783	FTT1091	(FTL_1111)	841751	841266	162	-	isochorismatase family protein	putative enzymes	unknown	
(FTN_0784)	FTT1090	FTL_1113	842442	842069						
FTN_0785	FTT1089	FTL_1114	842945	842442	168	-	isochorismatase family protein	putative enzymes	unknown	
FTN_0786	FTT1088c	FTL_1115	843533	844276	248	-	protein of unknown function	hypothetical - novel	unknown	
FTN_0787	FTT1087c	FTL_1116	844273	846285	671	rep	UvrD/REP superfamily I DNA and RNA helicases	DNA replication, recombination, modification and repair	unknown	
FTN_0788	FTT1086c	FTL_1117	846344	847171	276	-	conserved protein of unknown function	unknown function - conserved	unknown	
FTN_0789	FTT1085	FTL_1118	848631	847645	329	-	putative rhodanese, sulfurtransferase	putative enzymes	unknown	
FTN_0790	FTT1084c	FTL_1119	848825	849682	286	rdgC	recombination associated protein	DNA replication, recombination, modification and repair - restriction/modification	cytoplasm	
FTN_0791	FTT1083c	FTL_1120	849690	849944	85	-	protein of unknown function	unknown function - novel	cytoplasm	
FTN_0792	FTT1082	(1066124-1066546)	850385	849966	140	-	hypothetical protein	hypothetical - novel	unknown	
FTN_0793	(FTT1077c-FTT1078c-FTT1081c)	(FTL_0952-FTL_1124)	850477	851043	189	-	hypothetical protein	hypothetical - novel	unknown	
(FTN_0794)	FTT1076	FTL_1125	851999	852079						
FTN_0795	FTT1075	FTL_1126	852310	852101	70	-	transcriptional regulator	signal transduction and regulation	unknown	
FTN_0797	FTT0922	FTL_1288	853524	854069	182	-	fatty acid hydroxylase	fatty acids and lipids metabolism	unknown	
FTN_0798	FTT0923	FTL_1287	854146	856299	718	-	conserved protein of unknown function	unknown function - conserved	unknown	
FTN_0799	Schunoseq	LVSnoseq	856643	856317	109	emrE	putative membrane transporter of cations and cationic drugs, multidrug resistance protein	transport - drugs / antibacterial compounds	unknown	
FTN_0800	Schunoseq	LVSnoseq	858587	857562	342	-	ArsB arsenite/antimonite exporter	transport	unknown	
FTN_0801	Schunoseq	LVSnoseq	858947	858600	116	-	transcriptional regulator, ArsR family	signal transduction and regulation	unknown	

FTN_0802	FTT0924	FTL_1286	859091	859486	132	-	protein of unknown function	unknown function - novel	unknown	
FTN_0803	FTT0925	FTL_1285	859494	860432	313	fmt	methionyl-tRNA formyltransferase	translation, ribosomal structure and biogenesis	cytoplasm	
FTN_0804	FTT0926	FTL_1284	860439	861410	324	gshB	glutathione synthetase	cofactors, prosthetic groups, electron carriers metabolism	unknown	
FTN_0805	FTT0927	FTL_1283	861403	862701	433	hemL	glutamate-1-semialdehyde-2,1-aminomutase	cofactors, prosthetic groups, electron carriers metabolism	unknown	
FTN_0806	FTT0928c	FTL_1282	864387	862723	555	-	glycosyl hydrolase family 3	carbohydrate metabolism - biosynthesis	unknown	
FTN_0807	(FTT0929c)	(FTL_1280-FTL_1281)	865230	864451	260	-	short chain dehydrogenase	putative enzymes	unknown	yes
FTN_0808	(FTT0930c)	(FTL_1279)	865992	865255	246	-	acetoacetate decarboxylase	other metabolism - biosynthesis	unknown	yes
FTN_0809	FTT0931	FTL_1278	866270	867517	416	-	fucose: H+ symporter (FHS) family protein	transport - carbohydrates (sugars, polysaccharides)	cytoplasmic membrane	
FTN_0810	FTT0932	FTL_1277	867532	868413	294	-	ROK family protein	putative enzymes	unknown	
FTN_0811	(FTT0933)	(FTL_1276)	868439	869398	320	birA	biotin--acetyl-CoA-carboxylase ligase	fatty acids and lipids metabolism	unknown	
FTN_0812	FTT0934c	FTL_1275	870021	869347	225	bioD	dethiobiotin synthetase	cofactors, prosthetic groups, electron carriers metabolism	unknown	
FTN_0813	FTT0935c	FTL_1274	870763	870029	245	bioC	biotin synthesis protein BioC	cofactors, prosthetic groups, electron carriers metabolism	unknown	
FTN_0814	FTT0936c	FTL_1273	871868	870744	375	bioF	8-amino-7-oxononanoate synthase	cofactors, prosthetic groups, electron carriers metabolism	unknown	
FTN_0815	FTT0937c	FTL_1272	872803	871865	313	bioB	biotin synthase	cofactors, prosthetic groups, electron carriers metabolism	unknown	
FTN_0816	FTT0938	FTL_1271	872849	874177	443	bioA	adenosylmethionine-8-amino-7-oxononanoate aminotransferase	cofactors, prosthetic groups, electron carriers metabolism	unknown	
FTN_0817	FTT0940c	FTL_1267	874682	874185	166	-	conserved protein of unknown function	unknown function - conserved	cytoplasm	
FTN_0818	FTT0941c	FTL_1266	875618	874701	306	-	lipase/esterase	fatty acids and lipids metabolism	unknown	
FTN_0819	FTT0942c	FTL_1265	876896	875634	421	-	6-hydroxymethyl-7,8-dihydropterin pyrophosphokinase/dihydropteroate synthase	cofactors, prosthetic groups, electron carriers metabolism	unknown	
FTN_0820	FTT0943c	FTL_1264	877251	876901	117	folB	dihydroneopterin aldolase	cofactors, prosthetic groups, electron carriers metabolism	unknown	
FTN_0821	FTT0944	FTL_1263	877333	878700	456	-	AMP-binding enzyme	putative enzymes	unknown	
FTN_0822	FTT0945	FTL_1262	878696	880456	587	-	para-aminobenzoate synthase component I	cofactors, prosthetic groups, electron carriers metabolism	unknown	
FTN_0823	FTT0946	FTL_1261	880461	881012	184	pabA	para-aminobenzoate synthase component II	cofactors, prosthetic groups, electron carriers metabolism	unknown	
FTN_0824	(FTT0947c)	(FTL_1258-1203488)	882230	881019	404	-	major facilitator superfamily (MFS) transport protein	transport	cytoplasmic membrane	yes
FTN_0825	Schunoseq	LVSnoseq	883320	882250	357	-	aldo/keto reductase family protein	putative enzymes	unknown	
FTN_0826	FTT0948c	(FTL_1258)	884463	883402	354	-	aldo/keto reductase family protein	putative enzymes	unknown	
FTN_0827	FTT0949ca	(1200220-1200921)	885481	884558	308	-	carbon-nitrogen hydrolase family protein	putative enzymes	unknown	
FTN_0828	FTT0949cb	1199718-1200146	885984	885562	141	-	protein of unknown function	unknown function - novel	unknown	
FTN_0829	(FTT0950c)	(1199102-1199575)	886842	886126	239	-	conserved hypothetical membrane protein	hypothetical - conserved	cytoplasmic membrane	yes
FTN_0830	FTT0951c	FTL_1253	887452	886835	206	folE	GTP cyclohydrolase I	cofactors, prosthetic groups, electron carriers metabolism	unknown	
FTN_0831	FTT0952	FTL_1252	887579	888901	441	-	ATP-dependent RNA helicase	nucleotides and nucleosides metabolism	unknown	
FTN_0832	FTT0953c	FTL_1251	890442	888904	513	-	proton-dependent oligopeptide transporter (POT) family protein, di- or tripeptide:H+ symporter	transport	cytoplasmic membrane	
FTN_0833	FTT0954c	FTL_1249	890900	890583	106	-	conserved protein of unknown function	unknown function - conserved	unknown	
FTN_0834	FTT0955c	FTL_1248	892272	890914	453	gor	glutathione reductase	energy metabolism	unknown	
FTN_0835	FTT0956c	FTL_1247	893027	892311	239	-	protein of unknown function	unknown function - novel	unknown	
FTN_0836	FTT0957c	FTL_1246	893530	893030	167	-	kinase-like protein	putative enzymes	unknown	
FTN_0837	Schunoseq	LVSnoseq	894228	893533	232	-	hypothetical protein	hypothetical - novel	unknown	
FTN_0838	FTT0959c	FTL_1244	895017	894232	262	xthA	exodeoxyribonuclease III	DNA replication, recombination, modification and repair - restriction/modification	cytoplasm	
FTN_0839	FTT0960	(FTL_1243)	895194	896291	366	-	conserved protein of unknown function	unknown function - conserved	cytoplasm	
FTN_0840	FTT0961	LVSnoseq	896313	896900	196	mdaB	NADPH-quinone reductase (modulator of drug activity B)	putative enzymes	unknown	
FTN_0841	FTT0962	(FTL_1242)	897004	897678	225	-	ThiJ/PfpI family protein	putative enzymes	unknown	
FTN_0842	FTT0963c	FTL_1240	898863	897754	370	aroG	phospho-2-dehydro-3-deoxyheptonate aldolase	amino acid metabolism - biosynthesis	unknown	
FTN_0843	FTT0964c	FTL_1239	900271	898898	458	ffh	signal recognition particle GTPase	motility, attachment and secretion structure	unknown	
FTN_0844	976932-977198	FTL_1238	900618	900355	88	-	hypothetical membrane protein	hypothetical - novel	cytoplasmic membrane	
FTN_0845	FTT0965c	FTL_1237	901457	900621	279	-	conserved hypothetical membrane protein	hypothetical - conserved	cytoplasmic membrane	
FTN_0846	FTT0966	FTL_1236	901617	901832	72	infA	translation initiation factor IF-1	translation, ribosomal structure and biogenesis	unknown	
FTN_0847	(FTT0967c)	(1183203-1183748)	902790	901867	308	-	conserved hypothetical protein	hypothetical - conserved	unknown	
FTN_0848	FTT0968c	FTL_1233	904216	902798	473	-	amino acid antiporter	transport - amino-acid	cytoplasmic membrane	
FTN_0849	FTT0969c	FTL_1232	905648	904278	457	trkA	Trk system potassium uptake protein TrkA	transport	unknown	
FTN_0850	FTT0970	FTL_1231	905800	906207	136	-	transcriptional regulator	signal transduction and regulation	unknown	
FTN_0851	FTT0971	FTL_1230	906217	907659	481	sufB	sufS activator complex, sufB subunit	transport	unknown	
FTN_0852	FTT0972	FTL_1229	907701	908447	249	sufC	sufS activator complex, sufC subunit	transport	unknown	
FTN_0853	FTT0973	FTL_1228	908463	909605	381	sufD	sufS activator complex, sufD subunit	post-translational modification, protein turnover, chaperones	cytoplasmic membrane	
FTN_0854	(FTT0974)	(1175135-1175913)	909700	910479	260	-	conserved protein of unknown function	unknown function - conserved	unknown	
FTN_0855	FTT0975	FTL_1225	910576	911262	229	-	protein of unknown function	unknown function - novel	unknown	
FTN_0856	FTT0976	FTL_1224	911269	911592	108	-	thioredoxin	cofactors, prosthetic groups, electron carriers metabolism	unknown	
FTN_0857	(988165-988616)	(1174016-1173579)	911588	912034	149	-	conserved hypothetical protein	hypothetical - conserved	unknown	yes
FTN_0858	(FTT0977c)	(FTL_1222-1173766)	912289	911999	97	-	transcriptional regulator, MarR family	signal transduction and regulation	unknown	yes
FTN_0859	FTT0978c	FTL_1221	912898	912296	201	-	hypothetical membrane protein	hypothetical - novel	cytoplasmic membrane	
FTN_0860	FTT0979c	FTL_1220	914316	912967	450	-	amino acid transporter (AAT) family protein	transport - amino-acid	cytoplasmic membrane	
FTN_0861	FTT0980	FTL_1219	914550	915788	413	-	conserved protein of unknown function	unknown function - conserved	unknown	
FTN_0862	FTT0981	FTL_1218	916124	917245	374	-	hypothetical protein	hypothetical - novel	unknown	
FTN_0863	(FTT0982-FTT0983)	LVSnoseq	917341	917775	145	-	hypothetical membrane protein	hypothetical - novel	cytoplasmic membrane	
FTN_0864	Schunoseq	FTL_1217	918329	918697	123	-	protein of unknown function	unknown function - novel	unknown	
FTN_0865	FTT0984c	(1166465-1166674)	919291	918875	139	mutT	mutator protein	DNA replication, recombination, modification and repair - restriction/modification	cytoplasm	
FTN_0866	FTT0985	FTL_1216	919330	919911	194	-	conserved hypothetical protein	hypothetical - conserved	unknown	
FTN_0867	FTT0986	FTL_1215	919916	920323	136	-	conserved protein of unknown function	unknown function - conserved	cytoplasm	
FTN_0868	(FTT0987-FTT0988)	(FTL_1214-1165131)	920419	920994	192	-	membrane protein of unknown function	unknown function - conserved	outer membrane	yes
FTN_0869	FTT0989	FTL_1213	921132	923024	631	-	conserved protein of unknown function	unknown function - conserved	unknown	
FTN_0870	FTT0990	FTL_1212	923135	925573	813	leuS	leucyl-tRNA synthetase	other metabolism - biosynthesis	unknown	
FTN_0871	FTT0991	FTL_1211	925584	926156	191	-	rare lipoprotein B family protein	cell wall / LPS / capsule	unknown	
FTN_0872	FTT0992	(FTL_1209-FTL_1210)	926220	927407	396	-	small conductance mechanosensitive ion channel (MscS) family protein	transport	cytoplasmic membrane	
FTN_0873	FTT0993c	FTL_1208	927983	927420	188	dcd	deoxycytidine triphosphate deaminase	nucleotides and nucleosides metabolism	unknown	
FTN_0874	FTT0994c	FTL_1207	928855	927998	286	-	nucleotide-binding protein	cell cycle	unknown	
FTN_0875	FTT0995	(FTL_1205-FTL_1206)	928929	930158	410	-	metabolite:H+ symporter (MHS) family	transport	cytoplasmic membrane	
FTN_0876	(FTT0996)	FTL_1204	930215	931639	475	-	cardiolipin synthase	fatty acids and lipids metabolism	unknown	
FTN_0877	FTT0997	FTL_1203	931734	933161	476	cls	cardiolipin synthetase	fatty acids and lipids metabolism	unknown	
FTN_0878	FTT0998	FTL_1202	933333	934319	329	-	protein of unknown function	hypothetical - novel	unknown	
FTN_0879	(FTT0999c)	FTL_1201	935090	934347	248	-	zinc (Zn2+)-iron (Fe2+) permease (ZIP) family protein	transport	unknown	
FTN_0880	FTT1000c	FTL_1200	936310	935096	405	-	conserved protein of unknown function	unknown function - conserved	unknown	
FTN_0881	FTT1001	FTL_1199	936452	936880	143	-	Fe2+/Zn2+ uptake regulator protein	signal transduction and regulation	cytoplasm	
FTN_0882	FTT1002c	FTL_1198	939298	936929	790	pheT	phenylalanine tRNA synthetase, beta-subunit	other metabolism - biosynthesis	unknown	
FTN_0883	FTT1003c	FTL_1197	940331	939321	337	pheS	phenylalanyl-tRNA synthetase alpha-chain	other metabolism - biosynthesis	unknown	
FTN_0884	FTT1004c	FTL_1196	941310	940420	297	-	drug/metabolite transporter superfamily protein	transport - drugs / antibacterial compounds	unknown	
FTN_0885	FTT1005c	(FTL_1086)	942810	941338	491	-	proton-dependent oligopeptide transporter (POT) family protein, di- or tripeptide:H+ symporter	transport	cytoplasmic membrane	
FTN_0886	FTT1006	(FTL_1083-FTL_1085)	942996	944261	422	-	sugar:cation symporter family protein	transport - carbohydrates (sugars, polysaccharides)	cytoplasmic membrane	
FTN_0887	(FTT1007c-FTT1008c)	(1032677-FTL_1082)	945208	944252	319	-	hypothetical protein	hypothetical - novel	unknown	yes
FTN_0888	FTT1009	(FTL_1079-FTL_1080)	945336	945869	178	-	hypothetical membrane protein	hypothetical - novel	cytoplasmic membrane	
FTN_0889	FTT1010	FTL_1079	945887	946087	67	-	transcriptional regulator, cro/C1-type HTH domain	signal transduction and regulation	unknown	
FTN_0890	(FTT1011-FTT1012)	(FTL_0698-1031684)	946569	947333	255	-	conserved protein of unknown function	unknown function - conserved	unknown	yes
FTN_0891	FTT1013c	FTL_1077	948424	947381	348	ruvB	holliday junction DNA helicase, subunit B	DNA replication, recombination, modification and repair - restriction/modification	cytoplasm	

FTN 0892	FTT1014c	FTL 1076	949213	948491	241	-	short chain dehydrogenase	putative enzymes	unknown	
FTN 0893	FTT1015	FTL 1075	949331	949918	196	-	protein of unknown function	unknown function - novel	unknown	
FTN 0894	FTT1016c	FTL 1074	950560	949934	209	-	GDSL-like lipolytic enzyme	fatty acids and lipids metabolism	unknown	
FTN 0895	FTT1017c	FTL 1037	950917	950585	111	-	hypothetical protein	hypothetical - novel	unknown	
FTN 0896	FTT1018c	FTL 1072	952780	950936	615	dxs	1-deoxyxylulose-5-phosphate synthase	cofactors, prosthetic groups, electron carriers metabolism	unknown	
FTN 0897	FTT1019c	FTL 1071	954412	952865	516	guaA	GMP synthetase (glutamine-hydrolysing)	nucleotides and nucleosides metabolism	unknown	
FTN 0898	FTT1020c	(FTL_1069- FTL 1070)	956044	954503	514	-	amino acid permease	transport - amino-acid	cytoplasmic membrane	
FTN 0899	FTT1021c	FTL 1068	956876	956103	258	truA	tRNA pseudouridine synthase A	translation, ribosomal structure and biogenesis	unknown	
FTN 0900	FTT1022c	FTL 1067	958711	956882	610	-	protein of unknown function with predicted hydrolase and phosphorylase activity	unknown function - conserved	unknown	
FTN 0901	FTT1023c	FTL 1066	959317	958727	197	-	isomerase	putative enzymes	unknown	
FTN 0902	FTT1024c	FTL 1065	960097	959369	243	yhbG	ABC transporter, ATP-binding protein	transport	unknown	
FTN 0903	FTT1025c	FTL 1064	961004	960174	277	-	conserved protein of unknown function	unknown function - conserved	unknown	
FTN 0904	FTT1026c	FTL 1063	961617	960994	208	-	conserved protein of unknown function	unknown function - conserved	unknown	
FTN 0905	FTT1027c	FTL 1062	962173	961628	182	yrbI	3-deoxy-D-manno-octulosonate 8-phosphate phosphatase	fatty acids and lipids metabolism	unknown	
FTN 0906	FTT1028c	FTL 1061	962785	962267	173	ppa	inorganic pyrophosphatase	carbohydrate metabolism - degradation, utilization, assimilation	unknown	
FTN 0907	FTT1029	FTL 1060	962942	964273	444	-	D-alanyl-D-alanine carboxypeptidase	cell wall / LPS / capsule	unknown	
FTN 0908	FTT1030	FTL 1059	964309	964590	94	-	conserved protein of unknown function	unknown function - conserved	unknown	
FTN 0909	FTT1031	FTL 1058	964603	965220	206	lipB	lipoate-protein ligase B	cofactors, prosthetic groups, electron carriers metabolism	unknown	
FTN 0910	(FTT1032)	(1010740- 1012200)	965706	967175	490	-	sugar:cation symporter family protein	transport - carbohydrates (sugars, polysaccharides)	cytoplasmic membrane	
FTN 0911	(FTT1033)	(FTL_1052- 1010734)	967156	969192	679	-	glycosyl hydrolases family 31 protein	carbohydrate metabolism - biosynthesis	unknown	yes
FTN 0912	FTT1034c	FTL 1051	970570	969311	420	ndh	NADH dehydrogenase	energy metabolism	unknown	
FTN 0913	FTT1035c	FTL 1050	972410	970680	577	rpoD	RNA polymerase sigma-70 factor	transcription	unknown	
FTN 0914	FTT1036c	FTL 1049	974252	972438	605	dnaG	DNA primase	DNA replication, recombination, modification and repair - restriction/modification	cytoplasm	
FTN 0915	FTT1037c	FTL 1048	974719	974276	148	-	conserved protein of unknown function	unknown function - conserved	cytoplasm	
FTN 0916	FTT1038c	FTL 1047	974977	974783	65	-	30S ribosomal protein S21	translation, ribosomal structure and biogenesis	cytoplasm	
FTN 0917	FTT1039	FTL 1046	975232	976632	467	-	serine-type D-Ala-D-Ala carboxypeptidase	cell wall / LPS / capsule	unknown	
FTN 0918	FTT1040	FTL 1045	976636	977244	203	-	conserved protein of unknown function	unknown function - conserved	unknown	
FTN 0919	FTT1041	FTL 1044	977263	977676	138	-	thioesterase	putative enzymes	unknown	
FTN 0920	FTT1042	FTL 1043	977672	978472	267	-	predicted ATPase of the PP-loop superfamily	cell cycle	cytoplasm	
FTN 0921	FTT1043	FTL 1042	978489	979337	283	-	FKBP-type peptidyl-prolyl cis-trans isomerase	post-translational modification, protein turnover, chaperones - protein modification	unknown	
FTN 0922	FTT1044c	FTL 1041	980335	979361	325	ispB	octaprenyl diphosphate synthase	cofactors, prosthetic groups, electron carriers metabolism	unknown	
FTN 0923	FTT1045c	FTL 1040	980855	980427	143	-	protein of unknown function	unknown function - novel	unknown	
FTN 0924	FTT1046c	FTL 1039	981559	980939	207	-	hypothetical membrane protein	hypothetical - novel	cytoplasmic membrane	
FTN 0925	(FTT1047c- FTT1048c)	(FTL_1037- FTL 1038)	983200	982223	326	-	protein of unknown function	unknown function - novel	unknown	yes
FTN 0926	(FTT1049c)	(FTL 1036)	984254	983655	200	cysC	adenylylsulfate kinase	other metabolism - degradation, utilization, assimilation	unknown	yes
FTN 0927	FTT1050c	(FTL_1034- FTL 1035)	985686	984277	470	cysN	sulfate adenylate transferase, subunit 1	other metabolism - degradation, utilization, assimilation	unknown	
FTN 0928	Schunoseq	LVSnoseq	989818	988925	298	cysD	sulfate adenylyltransferase subunit 2	other metabolism - degradation, utilization, assimilation	unknown	
FTN 0929	Schunoseq	LVSnoseq	990945	989845	367	-	hypothetical protein	hypothetical - novel	cytoplasm	
FTN 0930	Schunoseq	LVSnoseq	991774	990929	282	-	protein of unknown function	unknown function - novel	unknown	
FTN 0931	Schunoseq	LVSnoseq	991793	992749	319	-	conserved protein of unknown function	unknown function - conserved	unknown	
FTN 0932	Schunoseq	LVSnoseq	992752	994548	599	-	ABC transporter, ATP-binding protein	transport	cytoplasmic membrane	
FTN 0933	Schunoseq	LVSnoseq	994548	994829	94	-	protein of unknown function	unknown function - novel	cytoplasm	
FTN 0934	Schunoseq	LVSnoseq	994829	995320	164	-	protein of unknown function	unknown function - novel	unknown	
FTN 0935	Schunoseq	LVSnoseq	995336	997186	617	asnB	asparagine synthase	amino acid metabolism - biosynthesis	unknown	
FTN 0936	Schunoseq	LVSnoseq	997186	998415	410	-	hypothetical protein	hypothetical - novel	cytoplasmic membrane	
FTN 0937	Schunoseq	LVSnoseq	998435	999334	300	-	conserved hypothetical protein	hypothetical - conserved	unknown	
FTN 0938	Schunoseq	LVSnoseq	999626	999438	63	-	hypothetical protein	hypothetical - novel	unknown	
FTN 0939	Schunoseq	LVSnoseq	1000177	1000022	52	-	hypothetical protein	hypothetical - novel	unknown	
FTN 0940	FTT1052c	(991063- 991257)	1000887	1000696	64	-	hypothetical protein	hypothetical - novel	unknown	
FTN 0941	(1062923- 1063055)	(990653- 990787)	1001330	1001163	56	-	hypothetical protein	hypothetical - novel	unknown	yes
FTN 0942	(1063209- 1063154)	990480- 990620	1001510	1001370	47	-	hypothetical protein	hypothetical - novel	unknown	
FTN 0943	FTT1054c	FTL 1032	1002259	1001822	146	rimI	ribosomal-protein-alanine acetyltransferase	translation, ribosomal structure and biogenesis	unknown	
FTN 0944	FTT1055c	FTL 1031	1002662	1002240	141	-	protein of unknown function	unknown function - novel	unknown	
FTN 0945	FTT1056c	FTL 1030	1003477	1002665	271	rsuA	16S rRNA pseudouridine synthase	translation, ribosomal structure and biogenesis	unknown	
FTN 0946	FTT1057c	FTL 1029	1004375	1003473	301	pilF	Type IV pili, pilus assembly protein	motility, attachment and secretion structure	unknown	
FTN 0947	FTT1058c	FTL 1028	1005477	1004368	370	-	protein of unknown function with radical SAM domain	unknown function - conserved	unknown	
FTN 0948	FTT1059c	FTL 1027	1006905	1005511	465	dnaB	replicative DNA helicase	DNA replication, recombination, modification and repair - restriction/modification	cytoplasm	
FTN 0949	FTT1060c	FTL 1026	1007448	1006996	151	rplI	50S ribosomal protein L9	translation, ribosomal structure and biogenesis	unknown	
FTN 0950	FTT1061c	FTL 1025	1007685	1007470	72	rpsR	30S ribosomal protein S18	translation, ribosomal structure and biogenesis	unknown	
FTN 0951	FTT1062c	FTL 1024	1008036	1007704	111	rpsF	30S ribosomal protein S6	translation, ribosomal structure and biogenesis	unknown	
FTN 0952	(1070770- 1071244)	(FTL 1023)	1008644	1008165	160	-	hypothetical protein	hypothetical - novel	unknown	yes
FTN 0953	FTT1063	FTL 1022	1008733	1009656	308	hemF	coproporphyrinogen III oxidase	cofactors, prosthetic groups, electron carriers metabolism	unknown	
FTN 0954	(FTT1064)	(FTL 1021)	1009684	1010889	402	-	histidine acid phosphatase	putative enzymes	unknown	
FTN 0955	FTT0560c	FTL 1018	1010967	1012016	350	serC	phosphoserine aminotransferase	amino acid metabolism - biosynthesis	unknown	
FTN 0956	FTT0559c	FTL 1017	1012006	1012668	221	cmk	cytidylate kinase	nucleotides and nucleosides metabolism	unknown	
FTN 0957	FTT0558	FTL 1016	1013299	1012691	203	-	short chain dehydrogenase	putative enzymes	unknown	
FTN 0958	FTT0557	FTL 1015	1013826	1013305	174	-	peroxiredoxin, AhpC-TSA family protein	other metabolism - degradation, utilization, assimilation	unknown	
FTN 0959	FTT0556c	FTL 1014	1013924	1014790	289	oxyR	oxidative stress transcriptional regulator	signal transduction and regulation	unknown	
FTN 0960	FTT0555	FTL 1013	1015527	1014799	243	-	conserved hypothetical membrane protein	hypothetical - conserved	cytoplasmic membrane	
FTN 0961	FTT0554	FTL 1012	1015813	1015544	90	-	conserved hypothetical protein	hypothetical - conserved	cytoplasm	
FTN 0962	FTT0553	(FTL_1009- 973406)	1016691	1015900	264	-	conserved protein of unknown function	unknown function - conserved	unknown	
FTN 0963	FTT0552	(FTL 1009)	1018192	1016699	498	-	NAD-dependent aldehyde dehydrogenase	other metabolism - degradation, utilization, assimilation	unknown	
FTN 0964	(FTT0551)	(971307- 972343)	1019321	1018209	371	-	dehydrogenase	putative enzymes	unknown	yes
FTN 0965	Schunoseq	LVSnoseq	1019741	1021147	469	-	metal-dependent exopeptidase	post-translational modification, protein turnover, chaperones - protein degradation	unknown	
FTN 0966	FTT0550	FTL 1005	1022996	1021590	469	-	conserved protein of unknown function	unknown function - conserved	unknown	
FTN 0967	FTT0549	FTL 1004	1023456	1022986	157	vanY	D-alanyl-D-alanine carboxypeptidase	cell wall / LPS / capsule	unknown	
FTN 0968	FTT0548	FTL 1003	1024142	1023453	230	dnaQ	DNA polymerase III, epsilon subunit	DNA replication, recombination, modification and repair - restriction/modification	cytoplasm	
FTN 0969	(FTT0546- FTT0547)	(FTL_1001- FTL 1002)	1025695	1024199	499	-	hypothetical protein	hypothetical - novel	unknown	yes
FTN 0970	FTT0545	FTL 1000	1025914	1025702	71	-	hypothetical protein	hypothetical - novel	unknown	
FTN 0971	FTT0544	(FTL 0999)	1026252	1025923	110	phnA	phosphonoacetate hydrolase	other metabolism - degradation, utilization, assimilation	unknown	
FTN 0972	FTT0543	(FTL_0997- FTL 0998)	1027734	1026361	458	-	hypothetical protein	hypothetical - novel	outer membrane	
FTN 0973	(FTT0542)	FTL 0996	1028398	1027802	199	-	peroxiredoxin of the AhpC/TSA family	cofactors, prosthetic groups, electron carriers metabolism	unknown	
FTN 0974	FTT0541c	FTL 0995	1028537	1029115	193	-	haloacid dehalogenase-like hydrolase	putative enzymes	cytoplasm	
FTN 0975	FTT0540c	FTL 0994	1029173	1030660	496	-	protein of unknown function	unknown function - novel	unknown	
FTN 0976	(FTT0539c)	(FTL_1642- FTL 0993)	1030667	1031407	247	-	ThiF family protein	putative enzymes	unknown	yes
FTN 0977	FTT0538c	FTL 0990	1031487	1032053	189	-	conserved protein of unknown function	unknown function - conserved	unknown	
FTN 0978	FTT0537	FTL 0989	1032700	1032068	211	-	ubiquinone biosynthesis protein COQ7	cofactors, prosthetic groups, electron carriers metabolism	unknown	
FTN 0979	FTT0536	FTL 0988	1033228	1032719	170	-	major facilitator superfamily (MFS) transport protein	transport	cytoplasmic membrane	
FTN 0980	FTT0535c	FTL 0987	1033379	1034335	319	mdh	malate dehydrogenase	energy metabolism	unknown	
FTN 0981	FTT0534c	FTL 0986	1034542	1036311	590	nrdA	ribonucleoside-diphosphate reductase, alpha subunit	nucleotides and nucleosides metabolism	unknown	

FTN 0982	FTT0533c	FTL 0985	1036318	1036575	86	grxA	glutaredoxin 1	post-translational modification, protein turnover, chaperones	unknown	
FTN 0983	FTT0532c	FTL 0984	1036585	1037814	410	-	bifunctional protein: glutaredoxin 3 /ribonucleotide reductase beta subunit	nucleotides and nucleosides metabolism	unknown	
FTN 0984	(FTT0531)	(952812-953855)	1039706	1037817	630	-	ABC transporter, ATP-binding protein	transport	unknown	
FTN 0985	FTT0530c	FTL 0982	1039817	1040416	200	-	DJ-1/PfpI family protein	putative enzymes	unknown	
FTN 0986	(FTT0529c)	FTL 0981	1040426	1041472	349	dinP	DNA-damage inducible protein P	DNA replication, recombination, modification and repair - restriction/modification	cytoplasm	
FTN 0987	FTT0519	FTL 0975	1043415	1042432	328	-	tRNA-dihydrouridine synthase	translation, ribosomal structure and biogenesis	cytoplasm	
FTN 0988	FTT0518	FTL 0974	1044250	1043408	281	prmA	50S ribosomal protein L11, methyltransferase	translation, ribosomal structure and biogenesis	unknown	
FTN 0989	(FTT0517)	942525-943679	1045569	1044415	385	yqhD	Fe-dependent alcohol dehydrogenase	energy metabolism	unknown	
FTN 0990	(FTT0516)	(FTL_0971-942507)	1048394	1045587	936	-	4Fe-4S ferredoxin, FAD dependent	energy metabolism	unknown	yes
FTN 0991	(FTT0514)	FTL 0970	1049855	1048716	380	lldD	L-lactate dehydrogenase	other metabolism - degradation, utilization, assimilation	unknown	
FTN 0992	FTT0691	FTL 0968	1051477	1050290	396	tyrS	tyrosyl-tRNA synthetase	other metabolism - biosynthesis	unknown	
FTN 0993	FTT0690c	FTL 0967	1051591	1052490	300	lplA	lipoate-protein ligase A	cofactors, prosthetic groups, electron carriers metabolism	unknown	
FTN 0994	FTT0689	FTL 0966	1053245	1052499	249	-	hypothetical membrane protein	hypothetical - novel	cytoplasmic membrane	
FTN 0995	FTT0688c	FTL 0965	1053437	1053985	183	hslV	ATP-dependent protease HslVU, peptidase subunit	post-translational modification, protein turnover, chaperones	unknown	
FTN 0996	FTT0687c	FTL 0964	1053996	1055360	455	hslU	ATP-dependent protease HslVU, ATPase subunit	post-translational modification, protein turnover, chaperones	unknown	
FTN 0997	FTT0686c	FTL 0963	1056320	1057768	483	-	proton-dependent oligopeptide transporter (POT) family protein, di- or tripeptide:H+ symporter	transport	cytoplasmic membrane	
FTN 0998	FTT0685c	(FTL_0961-FTL 0962)	1057851	1059008	386	-	potassium channel protein	transport	unknown	
FTN 0999	FTT0684c	FTL 0960	1059107	1060504	466	udhA	soluble pyridine nucleotide transhydrogenase	cofactors, prosthetic groups, electron carriers metabolism	unknown	
FTN 1000	FTT0683c	FTL 0959	1060511	1061356	282	pilD	Type IV pili leader peptidase and methylase	motility, attachment and secretion structure	unknown	
FTN 1001	FTT0682c	FTL 0958	1061369	1062211	281	-	protein of unknown function	unknown function - novel	periplasm	
FTN 1002	FTT0681c	FTL 0957	1062223	1063104	294	blaA	beta-lactamase class A	other metabolism - degradation, utilization, assimilation	unknown	
FTN 1003	FTT0680c	FTL 0956	1063100	1063672	191	pth	peptidyl-tRNA hydrolase	translation, ribosomal structure and biogenesis	unknown	
FTN 1004	FTT0679c	FTL 0955	1063694	1064782	363	ychF	GTP-binding protein	putative enzymes	unknown	
FTN 1005	FTT0678c	FTL 0954	1064800	1065075	92	-	hypothetical protein	hypothetical - novel	unknown	
FTN 1006	FTT0676	FTL 0951	1066374	1065118	419	-	transporter-associated protein, HlyC/CorC family	transport	cytoplasmic membrane	
FTN 1007	FTT0675	FTL 0950	1066760	1066473	96	rplY	50S ribosomal protein L25	translation, ribosomal structure and biogenesis	unknown	
FTN 1008	FTT0674	FTL 0949	1067828	1066863	322	prsA	ribose-phosphate pyrophosphokinase	nucleotides and nucleosides metabolism	unknown	
FTN 1009	FTT0673c	(FTL 0948)	1068039	1069043	335	-	conserved protein of unknown function	unknown function - conserved	unknown	
FTN 1010	(FTT0672c)	(916130-916999)	1069039	1070319	427	-	major facilitator superfamily (MFS) transport protein	transport	cytoplasmic membrane	
FTN 1011	FTT0671	(FTL 0946)	1071615	1070332	428	-	sugar transporter, MFS superfamily	transport - carbohydrates (sugars, polysaccharides)	cytoplasmic membrane	
FTN 1012	(FTT0670c)	(FTL 0945)	1071927	1072757	277	-	small conductance mechanosensitive ion channel family protein	transport	unknown	yes
FTN 1013	FTT0669	FTL 0943	1073913	1072741	391	-	monovalent cation:proton antiporter family protein	transport	cytoplasmic membrane	
FTN 1014	FTT0668	FTL 0942	1074643	1074014	210	-	nicotinamide ribonucleoside (NR) uptake permease (PnuC) family protein	transport	unknown	
FTN 1015	Schunoseq	LVSnoseq	1075146	1074646	167	-	isochorismatase family protein	putative enzymes	unknown	
FTN 1016	FTT0667	FTL 0941	1075756	1075256	167	-	conserved protein of unknown function	unknown function - conserved	cytoplasm	
(1075884-1076465)	FTT0666c	FTL 0940	1075884	1076465						
FTN 1018	FTT0665c	FTL 0939	1076556	1077251	232	-	aldolase/adducin class II family protein	carbohydrate metabolism - degradation, utilization, assimilation	unknown	
FTN 1019	FTT0664c	FTL 0938	1077269	1078402	378	hdc	pyridoxal-dependent decarboxylase	putative enzymes	unknown	
FTN 1020	FTT0663	FTL 0937	1079204	1078395	270	-	conserved protein of unknown function	unknown function - conserved	cytoplasm	
FTN 1021	(FTT0662c)	(FTL 0936)	1079294	1079827	178	-	conserved hypothetical protein	hypothetical - conserved	unknown	yes
FTN 1022	FTT0661c	FTL 0935	1079866	1080159	98	-	protein of unknown function	unknown function - novel	unknown	
FTN 1023	FTT0660	(FTL 0934)	1080771	1080187	195	-	hypothetical membrane protein	hypothetical - novel	cytoplasmic membrane	
FTN 1024	FTT0659	FTL 0933	1082235	1080823	471	-	RmuC family protein	putative enzymes	unknown	
FTN 1025	FTT0658	FTL 0932	1082911	1082264	216	ruvA	holliday junction DNA helicase, subunit A	DNA replication, recombination, modification and repair - restriction/modification	cytoplasm	
FTN 1026	(FTT0657)	FTL 0931	1084117	1082927	397	-	sugar transporter, MFS superfamily	transport - carbohydrates (sugars, polysaccharides)	cytoplasmic membrane	
FTN 1027	FTT0656	FTL 0930	1084714	1084202	171	ruvC	holliday junction endodeoxyribonuclease	DNA replication, recombination, modification and repair - restriction/modification	cytoplasm	
FTN 1028	FTT0655	FTL 0929	1085557	1084814	248	-	conserved protein of unknown function	unknown function - conserved	unknown	
FTN 1029	FTT0654	FTL 0928	1086232	1085576	219	-	conserved protein of unknown function	unknown function - conserved	unknown	
FTN 1030	FTT0653	FTL 0927	1087245	1086265	327	lipA	lipoic acid synthetase	cofactors, prosthetic groups, electron carriers metabolism	unknown	
FTN 1031	FTT0652c	FTL 0926	1087455	1087952	166	ftnA	ferric iron binding protein, ferritin-like	putative enzymes	unknown	
FTN 1032	FTT0651	(FTL_0924-FTL 0925)	1089430	1087961	490	-	proton-dependent oligopeptide transporter (POT) family protein, di- or tripeptide:H+ symporter	transport	cytoplasmic membrane	
FTN 1033	FTT0650c	FTL 0923	1089601	1090242	214	grxB	glutaredoxin 2	post-translational modification, protein turnover, chaperones	unknown	
FTN 1034	FTT0649c	FTL 0922	1090247	1090873	209	rnfB	iron-sulfur cluster-binding protein	putative enzymes	unknown	
FTN 1035	FTT0648c	(FTL_0921-FTL 0922)	1090869	1091504	212	nth	endonuclease III	DNA replication, recombination, modification and repair - restriction/modification	cytoplasm	
FTN 1036	FTT0647c	FTL 0920	1091504	1091932	143	-	protein of unknown function	unknown function - conserved	unknown	
FTN 1037	FTT0646c	FTL 0919	1091935	1093191	419	-	hypothetical membrane protein	hypothetical - novel	cytoplasmic membrane	
FTN 1038	FTT0645c	FTL 0918	1093310	1093993	228	-	conserved hypothetical membrane protein	hypothetical - conserved	cytoplasmic membrane	
FTN 1039	FTT0644c	FTL 0917	1094041	1097463	1141	mfd	transcription-repair coupling factor	transcription	unknown	
FTN 1040	(FTT0643)	FTL 0916	1098628	1097588	347	ilvC	ketol-acid reductoisomerase	amino acid metabolism - biosynthesis	unknown	
FTN 1041	FTT0642	FTL 0915	1098973	1098659	105	ilvN	acetolactate synthase small subunit	amino acid metabolism - biosynthesis	unknown	
FTN 1042	(FTT0641)	(886518-888109)	1100675	1098981	565	ilvB	acetolactate synthase large subunit	amino acid metabolism - biosynthesis	unknown	yes
FTN 1043	FTT0640	(885102-886368)	1102504	1100825	560	ilvD	dihydroxy-acid dehydratase	amino acid metabolism - biosynthesis	unknown	
FTN 1044	(FTT0637-FTT0638-FTT0639)	(883211-FTL 0910)	1104324	1102531	598	-	conserved protein of unknown function	unknown function - conserved	unknown	yes
FTN 1045	FTT0636	FTL 0906	1105060	1104470	197	engB	ATP/GTP-binding protein	putative enzymes	unknown	
FTN 1046	FTT0635	FTL 0905	1105542	1105060	161	wzb	low molecular weight (LMW) phosphotyrosine protein phosphatase	post-translational modification, protein turnover, chaperones - protein modification	unknown	
FTN 1047	FTT0634	FTL 0904	1106469	1105546	308	hflC	HflK-HflC membrane protein complex, HflC	post-translational modification, protein turnover, chaperones - protein degradation	unknown	
FTN 1048	FTT0633	FTL 0903	1107538	1106474	355	hflK	HflK-HflC membrane protein complex, HflK	post-translational modification, protein turnover, chaperones - protein degradation	unknown	
FTN 1049	FTT0632c	(FTL_0900-FTL 0902)	1107723	1108898	392	-	oxidoreductase	putative enzymes	unknown	
FTN 1050	FTT0631	FTL 0899	1110222	1108915	436	hflX	protease, GTP-binding subunit	putative enzymes	unknown	
FTN 1051	FTT0630	FTL 0898	1110600	1110274	109	hfq	host factor I for bacteriophage Q beta replication	mobile and extrachromosomal element functions - phage or plasmid related proteins	unknown	
FTN 1052	FTT0629	FTL 0897	1111637	1110714	308	miaA	tRNA delta(2)-isopentenylpyrophosphate transferase	translation, ribosomal structure and biogenesis	cytoplasm	
FTN 1053	FTT0628	FTL 0896	1113071	1111641	477	-	outer membrane protein of unknown function	unknown function - novel	outer membrane	
FTN 1054	FTT0627	FTL 0895	1113456	1113187	90	hupB	DNA-binding protein HU-beta	DNA replication, recombination, modification and repair - restriction/modification	cytoplasm	
FTN 1055	FTT0626	FTL 0894	1115867	1113546	774	lon	DNA-binding, ATP-dependent protease La	post-translational modification, protein turnover, chaperones	unknown	
FTN 1056	FTT0625	FTL 0893	1117145	1115895	417	clpX	ATP-dependent Clp protease subunit X	post-translational modification, protein turnover, chaperones - protein degradation	cytoplasm	
FTN 1057	FTT0624	FTL 0892	1117774	1117172	201	clpP	ATP-dependent Clp protease subunit P	post-translational modification, protein turnover, chaperones - protein degradation	cytoplasm	
FTN 1058	FTT0623	FTL 0891	1119116	1117803	438	tig	trigger factor (TF) protein (peptidyl-prolyl cis/trans isomerase)	post-translational modification, protein turnover, chaperones - protein modification	cytoplasm	
FTN 1059	FTT0622c	868267-867803	1119271	1119732	154	-	hypothetical protein	hypothetical - novel	unknown	
FTN 1060	FTT0621	FTL 0890	1120371	1119781	197	tdk	thymidine kinase	nucleotides and nucleosides metabolism	cytoplasm	

FTN 1061	FTT0620	(866408- FTL 0889)	1121136	1120390	249	-	acid phosphatase, HAD superfamily protein	putative enzymes	cytoplasm	
FTN 1062	FTT0619	FTL 0887	1121990	1121319	224	-	o-methyltransferase family protein	putative enzymes	cytoplasm	
FTN 1063	FTT0618c	FTL 0886	1122215	1123540	442	-	tRNA-methylthiotransferase MiaB protein	translation, ribosomal structure and biogenesis	cytoplasm	
FTN 1064	FTT0617c	FTL 0885	1123547	1124527	327	-	PhoH family protein, putative ATPase	signal transduction and regulation	unknown	
FTN 1065	FTT0616c	FTL 0884	1124523	1125008	162	-	conserved protein of unknown function	unknown function - conserved	cytoplasm	
FTN 1066	FTT0615c	FTL 0883	1125001	1125840	280	-	transporter-associated protein, HlyC/CorC family	transport	cytoplasm	
FTN 1067	FTT0614c	FTL 0882	1125843	1127330	496	-	apolipoprotein N-acyltransferase	cell wall / LPS / capsule	cytoplasmic membrane	
FTN 1068	FTT0613c	(859722- FTL 0881)	1127581	1128000	140	-	hypothetical protein	hypothetical - novel	unknown	
FTN 1069	Schunoseq	LVSnoseq	1130653	1128158	832	-	protein of unknown function	unknown function - novel	unknown	
FTN 1070	Schunoseq	LVSnoseq	1132213	1130864	450	-	protein of unknown function	unknown function - novel	cytoplasm	
FTN 1071	(FTT0612)	(FTL 0880)	1133899	1132424	492	-	protein of unknown function	unknown function - novel	cytoplasm	
FTN 1072	FTT0611c	FTL 0879	1134341	1135201	287	-	beta-lactamase class A	other metabolism - degradation, utilization, assimilation	periplasm	
FTN 1073	FTT0610	FTL 0878	1136259	1135198	354	-	DNA/RNA endonuclease G	nucleotides and nucleosides metabolism	extracellular	
FTN 1074	FTT0609	FTL 0877	1138117	1136360	586	-	X-prolyl aminopeptidase 2	post-translational modification, protein turnover, chaperones - protein modification	cytoplasm	
FTN 1075	FTT0608	FTL 0876	1138876	1138172	235	-	conserved protein of unknown function	unknown function - conserved	unknown	
FTN 1076	FTT0607	FTL 0875	1140108	1138894	405	ispG	1-hydroxy-2-methyl-2-(E)-butenyl 4-diphosphate synthase	cofactors, prosthetic groups, electron carriers metabolism	cytoplasm	
FTN 1077	(FTT0606c)	(852097- 852336)	1140193	1140936	248	-	conserved protein of unknown function	unknown function - conserved	unknown	
(FTN_1078)	Schunoseq	LVSnoseq	1140939	1141115						
FTN 1079	(FTT0600)	FTL 0865	1142637	1141288	450	-	sugar porter (SP) family	transport - carbohydrates (sugars, polysaccharides)	cytoplasmic membrane	
FTN 1080	FTT0599	FTL 0864	1143699	1142674	342	-	phosphosugar binding protein	cell wall / LPS / capsule	unknown	
FTN 1081	FTT0598c	FTL 0863	1143901	1145172	424	-	dicarboxylate/amino acid:cation (Na+ or H+) symporter	transport - amino-acid	cytoplasmic membrane	
FTN 1082	FTT0597	FTL 0862	1146601	1145210	464	-	conserved protein of unknown function	unknown function - conserved	unknown	
FTN 1083	FTT0596c	FTL 0860	1146713	1147327	205	-	hypothetical protein	hypothetical - novel	cytoplasm	
FTN 1084	FTT0595c	FTL 0859	1147417	1147584	56	rubA	rubredoxin	energy metabolism	unknown	
FTN 1085	FTT0594c	FTL 0858	1147596	1148465	290	-	conserved hypothetical protein	hypothetical - conserved	unknown	
FTN 1086	FTT0593c	FTL 0857	1148532	1149134	201	-	hypothetical membrane protein	hypothetical - novel	unknown	
FTN 1087	FTT0592	FTL 0856	1150110	1149427	228	cynT	carbonic anhydrase	other metabolism - degradation, utilization, assimilation	unknown	
FTN 1088	FTT0591	FTL 0855	1151218	1150184	345	ansA	L-asparaginase	amino acid metabolism - degradation, utilization, assimilation	unknown	
FTN 1089	FTT0590	FTL 0854	1151678	1151223	152	rnhA	ribonuclease H	DNA replication, recombination, modification and repair - restriction/modification	cytoplasm	
FTN 1090	FTT0589	FTL 0853	1152243	1151656	196	-	membrane protein of unknown function	unknown function - novel	cytoplasmic membrane	
FTN 1091	FTT0588	FTL 0852	1153523	1152249	425	aroA	3-phosphoshikimate 1-carboxyvinyltransferase	amino acid metabolism - biosynthesis	unknown	
FTN 1092	FTT1112c	FTL 0851	1154492	1153617	292	rpoH	RNA polymerase sigma-32 factor	transcription	unknown	
FTN 1093	FTT1113c	FTL 0850	1155004	1154519	162	-	protein of unknown function	unknown function - novel	unknown	
FTN 1094	FTT1114c	FTL 0849	1156022	1155081	314	secF	preprotein translocase, subunit F, membrane protein	motility, attachment and secretion structure	cytoplasmic membrane	
FTN 1095	FTT1115c	FTL 0848	1157978	1156044	645	secD	preprotein translocase, subunit D, membrane protein	motility, attachment and secretion structure	unknown	
FTN 1096	FTT1116c	FTL 0847	1158391	1158038	118	yajC	preprotein translocase family protein	motility, attachment and secretion structure	unknown	
FTN 1097	FTT1117c	FTL 0846	1159515	1158946	190	-	isochorismatase family protein	putative enzymes	unknown	
FTN 1098	FTT1118c	(FTL 0845)	1160402	1159530	291	-	conserved hypothetical membrane protein	hypothetical - conserved	cytoplasmic membrane	
FTN 1099	FTT1119	(FTL 0844)	1160507	1161379	291	-	transcriptional regulator, LysR family	signal transduction and regulation	cytoplasm	
FTN 1100	FTT1120c	FTL 0843	1162492	1161392	367	tgt	queuine tRNA-ribosyltransferase.	translation, ribosomal structure and biogenesis	unknown	
FTN 1101	Schunoseq	LVSnoseq	1163790	1162960	277	-	protein of unknown function	unknown function - novel	unknown	
FTN 1103	Schunoseq	LVSnoseq	1165492	1164647	282	-	protein of unknown function	unknown function - novel	unknown	
FTN 1104	FTT1122c	(FTL_0840- FTL 0841)	1166067	1165522	182	-	hypothetical protein	hypothetical - novel	unknown	
FTN 1105	FTT1123	FTL 0839	1166699	1167310	204	-	conserved membrane protein of unknown function	unknown function - conserved	cytoplasmic membrane	
FTN 1106	FTT1124	FTL 0838	1167276	1168349	358	metN	methionine uptake transporter (MUT) family protein	transport - amino-acid	unknown	
FTN 1107	FTT1125	FTL 0837	1168330	1169799	490	metIQ	methionine uptake transporter (MUT) family protein, membrane and periplasmic protein	transport - amino-acid	unknown	
FTN 1108	FTT1126	(FTL_0835- FTL 0836)	1169873	1171081	403	-	hydroxy/aromatic amino acid permease (HAAAP) family protein	transport - amino-acid	cytoplasmic membrane	
FTN 1109	FTT1127	FTL 0834	1171158	1171904	249	-	rhodanese-like family protein	putative enzymes	unknown	
FTN 1110	FTT1128	FTL 0833	1171920	1172396	159	ispF	2-C-methyl-D-erythritol 2,4-cyclodiphosphate synthase	cell wall / LPS / capsule	unknown	
FTN 1111	FTT1129c	FTL 0832	1174089	1172407	561	-	Mur ligase family protein	cell wall / LPS / capsule	unknown	
FTN 1112	FTT1130c	FTL 0831	1176937	1174118	940	cphA	cyanophycin synthetase	cell wall / LPS / capsule	unknown	
FTN 1113	(FTT1131)	FTL 0830	1177061	1177801	247	-	conserved protein of unknown function	unknown function - conserved	unknown	
FTN 1114	FTT1132c	FTL 0829	1178545	1177796	250	glpQ	glycerophosphoryl diester phosphodiesterase	other metabolism - degradation, utilization, assimilation	cytoplasm	
FTN 1115	FTT1133	FTL 0828	1178703	1180478	592	pilB	Type IV pili ATPase	motility, attachment and secretion structure	cytoplasm	
FTN 1116	FTT1134	FTL 0827	1180496	1181722	409	pilC	Type IV pili polytopic inner membrane protein	motility, attachment and secretion structure	unknown	
FTN 1117	(FTT1135c)	(806009- 807160)	1182965	1181802	388	-	ATP binding protein	putative enzymes	unknown	yes
FTN 1118	FTT1136c	FTL 0823	1183318	1182986	111	-	hypothetical protein	hypothetical - novel	unknown	
FTN 1119	FTT1137c	FTL 0822	1183586	1183338	83	-	protein of unknown function	unknown function - novel	unknown	
FTN 1120	FTT1138	FTL 0821	1183729	1184727	333	hemH	ferrochelatase	cofactors, prosthetic groups, electron carriers metabolism	unknown	
FTN 1121	(FTT1139)	(802010- 803239)	1184715	1186106	464	phrB	deoxyribodipyrimidine photolyase	DNA replication, recombination, modification and repair - restriction/modification	cytoplasm	
FTN 1122	(FTT1140)	(FTL 0816)	1186844	1187230	129	-	hypothetical protein	hypothetical - novel	unknown	yes
FTN 1123	(FTT1141)	(FTL_0814- FTL 0815)	1187288	1187590	101	-	conserved hypothetical protein	hypothetical - conserved	unknown	
FTN 1124	FTT1143	(799768- 799971)	1187659	1187901	81	-	hypothetical protein	hypothetical - novel	unknown	
FTN 1125	(FTT1144)	(798721- 799607)	1188089	1188949	287	-	short chain dehydrogenase	putative enzymes	unknown	
FTN 1126	FTT1145	FTL 0810	1188964	1189191	76	chaB	putative membrane ion antiporter regulator	signal transduction and regulation	unknown	
FTN 1127	(FTT1146c)	(797400- 798364)	1190299	1189307	331	galM	aldose 1-epimerase	carbohydrate metabolism - degradation, utilization, assimilation	unknown	yes
FTN 1128	FTT1147c	FTL 0808	1191471	1190299	391	dfp	P-pantothenate cysteine ligase/P-pantothenoylcysteine decarboxylase	cofactors, prosthetic groups, electron carriers metabolism	unknown	
FTN 1129	FTT1148c	FTL 0807	1192668	1191487	394	-	major facilitator superfamily (MFS) transport protein	transport	cytoplasmic membrane	
FTN 1130	FTT1149c	FTL 0806	1194252	1192747	502	-	amino acid-polyamine-organocation (APC) superfamily	transport - amino-acid	cytoplasmic membrane	
FTN 1131	FTT1150c	FTL 0805	1198477	1194416	1354	putA	bifunctional proline dehydrogenase, pyrroline-5-carboxylate dehydrogenase	amino acid metabolism - degradation, utilization, assimilation	unknown	
FTN 1132	FTT1151c	FTL 0804	1198781	1198497	95	-	conserved protein of unknown function	unknown function - conserved	unknown	
FTN 1133	FTT1152	FTL 0803	1198995	1199375	127	-	protein of unknown function	unknown function - novel	unknown	
FTN 1134	FTT1153c	(788009- 788255)	1199690	1199448	81	-	hypothetical protein	hypothetical - novel	unknown	
FTN 1135	FTT1154c	FTL 0802	1200805	1199729	359	aroB	3-dehydroquinate synthetase	amino acid metabolism - biosynthesis	unknown	
FTN 1136	FTT1155c	FTL 0801	1201332	1200805	176	aroK	shikimate kinase I	amino acid metabolism - biosynthesis	cytoplasm	
FTN 1137	FTT1156c	FTL 0800	1203242	1201461	594	pilQ	Type IV pili secretin component	motility, attachment and secretion structure	outer membrane	
FTN 1138	FTT1157c	FTL 0799	1203845	1203258	196	pilP	Type IV pili periplasmic component	motility, attachment and secretion structure	unknown	
FTN 1139	FTT1158c	FTL 0798	1204447	1203854	198	pilO	Type IV pili glycosylation protein	motility, attachment and secretion structure	unknown	
FTN 1140	FTT1159c	FTL 0797	1205004	1204444	187	pilN	Type IV pili associated protein	motility, attachment and secretion structure	unknown	
FTN 1141	FTT1160c	FTL 0796	1206010	1205009	334	pilM	Type IV pili, pilus assembly protein	motility, attachment and secretion structure	unknown	
FTN 1142	FTT1161	FTL 0795	1206137	1206790	218	adk	adenylate kinase	nucleotides and nucleosides metabolism	cytoplasm	
FTN 1143	(FTT1162c)	(777639- 780356)	1210072	1207355	906	-	4Fe-4S ferredoxin, FAD dependent	energy metabolism	unknown	
FTN 1144	FTT1163c	(777223- FTL 0791)	1210549	1210136	138	-	hypothetical membrane protein	hypothetical - novel	cytoplasmic membrane	
FTN 1145	FTT1164c	FTL 0790	1211457	1210567	297	era	GTP-binding protein	putative enzymes	cytoplasm	
FTN 1146	FTT1165c	FTL 0789	1212764	1211574	397	-	aspartate aminotransferase	other metabolism - degradation, utilization, assimilation	unknown	
FTN 1147	FTT1166c	FTL 0788	1213580	1212777	268	-	glutamine amidotransferases class-II family protein	putative enzymes	unknown	
FTN 1148	FTT1167c	FTL 0787	1214222	1213587	212	-	glycoprotease family protein	post-translational modification, protein turnover, chaperones	unknown	
FTN 1149	FTT1168c	FTL 0786	1215359	1214229	377	nagA	N-acetylglucosamine-6-phosphate deacetylase	carbohydrate metabolism - degradation, utilization, assimilation	unknown	
FTN 1150	FTT1169	FTL 0785	1215422	1216291	290	-	GTP-binding protein	putative enzymes	unknown	
FTN 1151	FTT1170	FTL 0784	1216361	1218106	582	-	conserved protein of unknown function	unknown function - conserved	extracellular	
FTN 1152	FTT1171c	(767186- 768513)	1219547	1218102	482	-	type I restriction-modification system, subunit M (methyltransferase)	DNA replication, recombination, modification and repair - repair	cytoplasm	
FTN 1153	Schunoseq	LVSnoseq	1220226	1219531	232	-	conserved protein of unknown function	unknown function - conserved	cytoplasm	

FTN	1154	(FTT1173c)	(765521-766728)	1222016	1220796	407	-	type I restriction-modification system, subunit S	DNA replication, recombination, modification and repair - repair	unknown	yes
FTN	1155	(FTT1176c)	(761880-764213)	1224969	1222624	782	-	type I restriction-modification system, subunit R (restriction)	DNA replication, recombination, modification and repair - repair	unknown	yes
FTN	1156	(FTT1177c-FTT1178c)	(760253-FTL 0769)	1226655	1225105	517	-	hypothetical protein	hypothetical - novel	cytoplasm	yes
FTN	1157	FTT1179	FTL 0768	1227077	1228891	605	-	GTP binding translational elongation factor Tu and G family protein	putative enzymes	unknown	
FTN	1158	(FTT1180)	FTL 0767	1229066	1229818	251	-	methyltransferase	putative enzymes	unknown	
FTN	1159	FTT1181c	FTL 0766	1231642	1229840	601	ggt	gamma-glutamyltranspeptidase	cofactors, prosthetic groups, electron carriers metabolism	unknown	
FTN	1160	(FTT1182c)	FTL 0765	1232618	1231701	306	-	VacJ like lipoprotein	cell wall / LPS / capsule	cytoplasmic membrane	
FTN	1161	FTT1183c	FTL 0764	1233897	1232698	400	-	conserved hypothetical protein	hypothetical - conserved	unknown	
FTN	1162	FTT1184c	FTL 0763	1234244	1233942	101	-	conserved protein of unknown function	unknown function - conserved	cytoplasm	
FTN	1163	FTT1185c	FTL 0762	1234675	1234247	143	-	oligoketide cyclase/lipid transport protein	fatty acids and lipids metabolism	unknown	
FTN	1164	FTT1186	FTL 0761	1234750	1235220	157	smpB	SsrA (tmRNA)-binding protein	translation, ribosomal structure and biogenesis	unknown	
FTN	1165	FTT1187	FTL 0760	1235224	1235982	253	-	predicted ATPase of the PP-loop superfamily	cell cycle	cytoplasm	
FTN	1166	(FTT1189c)	(748466-749718)	1237257	1235989	423	-	metabolite:H+ symporter (MHS) family protein	transport	cytoplasmic membrane	
FTN	1167	1205964-1205775	FTL 0755	1237555	1237358	66	-	protein of unknown function	unknown function - novel	unknown	
FTN	1168	FTT1190c	FTL 0754	1238924	1237542	461	xseA	exodeoxyribonuclease VII large subunit	DNA replication, recombination, modification and repair - restriction/modification	cytoplasm	
FTN	1169	FTT1191	(FTL_0753-746646)	1239077	1240234	386	-	peptidase, M20 family	post-translational modification, protein turnover, chaperones - protein modification	unknown	
FTN	1170	(FTT1192c-FTT1193c)	FTL 0752	1242037	1240289	583	-	conserved protein of unknown function	unknown function - conserved	unknown	
FTN	1171	(FTT1194c)	(742654-FTL 0751)	1243040	1242057	328	-	conserved protein of unknown function	unknown function - conserved	unknown	yes
FTN	1172	(FTT1195c)	(741519-742675)	1244494	1243043	484	-	conserved protein of unknown function	unknown function - conserved	unknown	
FTN	1173	FTT1196c	FTL 0748	1245932	1244658	425	-	metabolite:H+ symporter (MHS) family protein	transport	cytoplasmic membrane	
FTN	1174	FTT1197c	FTL 0747	1246820	1246026	265	murI	glutamate racemase	cell wall / LPS / capsule	unknown	
FTN	1175	FTT1198c	FTL 0746	1247617	1246823	265	-	membrane protein of unknown function	unknown function - novel	cytoplasmic membrane	
FTN	1176	FTT1199c	FTL 0745	1249660	1247657	668	uvrB	excinuclease ABC, subunit B	DNA replication, recombination, modification and repair - restriction/modification	cytoplasm	
FTN	1177	FTT1200c	FTL 0744	1251110	1249665	482	sbcB	exodeoxyribonuclease I	DNA replication, recombination, modification and repair - restriction/modification	cytoplasm	
FTN	1178	FTT1201c	FTL 0743	1251966	1251247	240	-	short-chain dehydrogenase	other metabolism - degradation, utilization, assimilation	unknown	
FTN	1179	FTT1202	(FTL 0742)	1252082	1252978	299	-	transcriptional regulator, LysR family	signal transduction and regulation	unknown	
FTN	1180	FTT1203c	FTL 0741	1253897	1252995	301	-	hypothetical membrane protein	hypothetical - novel	cytoplasmic membrane	
FTN	1181	FTT1204c	FTL 0740	1254966	1253887	360	-	hypothetical membrane protein	hypothetical - novel	cytoplasmic membrane	
FTN	1182	FTT1205	FTL 0739	1255109	1256989	627	gidA	glucose inhibited division protein A	cell cycle	unknown	
FTN	1183	FTT1206	FTL 0738	1257004	1257393	130	-	protein of unknown function	unknown function - novel	unknown	
FTN	1184	FTT1207	FTL 0737	1257410	1258777	456	-	hypothetical membrane protein	hypothetical - novel	cytoplasmic membrane	
FTN	1185	FTT1208	FTL 0736	1258782	1259453	224	rpiA	ribose 5-phospate isomerase A	carbohydrate metabolism - degradation, utilization, assimilation	unknown	
FTN	1186	(FTT1209c)	(FTL_1401-725360)	1261567	1259507	687	pepO	M13 family metallopeptidase	post-translational modification, protein turnover, chaperones - protein degradation	unknown	yes
FTN	1187	FTT0821	FTL 1403	1262360	1261659	234	-	protein of unknown function	unknown function - conserved	cytoplasmic membrane	
FTN	1188	FTT0820	FTL 1404	1262758	1262405	118	rplT	50S ribosomal protein L20	translation, ribosomal structure and biogenesis	unknown	
FTN	1189	FTT0819	FTL 1405	1262991	1262797	65	rpmI	50S ribosomal protein L35	translation, ribosomal structure and biogenesis	unknown	
FTN	1190	(FTT0818)	(FTL 1406)	1263570	1263037	178	infC	translation initiation factor IF-3	translation, ribosomal structure and biogenesis	cytoplasm	
FTN	1191	FTT0817	FTL 1407	1265481	1263580	634	thrS	threonyl-tRNA synthetase	other metabolism - biosynthesis	cytoplasm	
FTN	1192	(FTT0814c-FTT0815c-FTT0816c)	FTL 1408	1265920	1267707	596	-	chitin-binding protein	putative enzymes	unknown	
FTN	1193	FTT0813	(1338073-1338367)	1268091	1267798	98	-	hypothetical protein	hypothetical - novel	unknown	
FTN	1194	FTT0812	FTL 1409	1268461	1268084	126	gcvH1	glycine cleavage system protein H	amino acid metabolism - degradation, utilization, assimilation	unknown	
FTN	1195	FTT0811c	FTL 1410	1268565	1269677	371	murG	UDP-N-acetylglucosamine--N-acetylmuramyl-(pentapeptide) pyrophosphoryl-undecaprenol N-acetylglucosamine transferase	cell wall / LPS / capsule	cytoplasmic membrane	
FTN	1196	FTT0810c	FTL 1411	1269690	1270025	112	-	conserved protein of unknown function	unknown function - conserved	unknown	
FTN	1197	FTT0809c	FTL 1412	1270028	1270627	200	recR	RecFOR complex, RecR component	DNA replication, recombination, modification and repair - restriction/modification	cytoplasm	
FTN	1198	FTT0808	FTL 1413	1272752	1270641	704	spoT	GDP diphosphokinase/guanosine-3',5'-bis(diphosphate) 3'-diphosphatase	other metabolism - biosynthesis	cytoplasm	
FTN	1199	FTT0807	FTL 1414	1274032	1272824	403	-	conserved protein of unknown function	unknown function - conserved	unknown	
FTN	1200	FTT0806	FTL 1415	1274498	1274037	154	capC	capsule biosynthesis protein CapC	cell wall / LPS / capsule	unknown	
FTN	1201	FTT0805	FTL 1416	1275740	1274526	405	capB	capsule biosynthesis protein CapB	cell wall / LPS / capsule	unknown	
FTN	1202	FTT0804	FTL 1417	1277072	1275819	418	-	metabolite:H+ symporter (MHS) family protein	transport	cytoplasmic membrane	
FTN	1208	FTT0803	FTL 1418	1278606	1277746	287	ans	asparaginase	amino acid metabolism - degradation, utilization, assimilation	unknown	
FTN	1209	FTT0802	FTL 1419	1279639	1278824	272	cphB	cyanophycinase	post-translational modification, protein turnover, chaperones - protein modification	unknown	
FTN	1210	FTT0801c	FTL 1420	1279841	1280944	368	-	ribokinase, pfkB family	carbohydrate metabolism	unknown	
FTN	1211	FTT0800	FTL 1421	1281900	1281241	220	-	haloacid dehalogenase-like hydrolase	putative enzymes	unknown	
FTN	1212	FTT0799	FTL 1422	1282932	1281922	337	-	glycosyl transferase, group 1	cell wall / LPS / capsule	unknown	
FTN	1213	FTT0798	FTL 1423	1283942	1282938	335	-	glycosyl transferase, family 2	cell wall / LPS / capsule	unknown	
FTN	1214	FTT0797	FTL 1424	1284918	1283962	319	-	glycosyl transferase, family 2	cell wall / LPS / capsule	unknown	
FTN	1215	Schunoseq	LVSnoseq	1286112	1284955	386	kpsC	capsule polysaccharide export protein KpsC	cell wall / LPS / capsule	cytoplasm	
FTN	1216	Schunoseq	LVSnoseq	1286848	1286108	247	-	protein of unknown function	unknown function - novel	unknown	
FTN	1217	FTT0793	FTL 1428	1288573	1286888	562	-	ATP-binding cassette (ABC) superfamily protein	transport	unknown	
FTN	1218	FTT0792	FTL 1429	1289799	1288573	409	-	glycosyl transferase, group 1	cell wall / LPS / capsule	unknown	
FTN	1219	FTT0791	FTL 1430	1290822	1289806	339	galE	UDP-glucose 4-epimerase	carbohydrate metabolism - degradation, utilization, assimilation	unknown	
FTN	1220	FTT0790	FTL 1431	1292209	1290818	464	-	sugar transferase involved in lipopolysaccharide synthesis	cell wall / LPS / capsule	cytoplasmic membrane	
FTN	1221	FTT0789	FTL 1432	1292858	1292193	222	rpe	D-ribulose-phosphate 3-epimerase	energy metabolism	cytoplasm	
FTN	1222	FTT0788c	FTL 1433	1292995	1293963	323	kpsF	phosphosugar isomerase	cell wall / LPS / capsule	unknown	
FTN	1223	FTT0787	FTL 1434	1294560	1293973	196	-	conserved hypothetical membrane protein	hypothetical - conserved	cytoplasmic membrane	
FTN	1224	FTT0786	(FTL_1435-FTL 1436)	1295269	1294574	232	-	conserved hypothetical protein	hypothetical - conserved	unknown	
FTN	1225	FTT0785	FTL 1437	1296078	1295269	270	-	conserved hypothetical protein	hypothetical - conserved	cytoplasm	
FTN	1226	FTT0784	FTL 1438	1296404	1296078	109	-	protein of unknown function	unknown function - novel	unknown	
FTN	1227	FTT0783	(FTL_1439-FTL 1440)	1297378	1296419	320	-	metallo-beta-lactamase superfamily protein	putative enzymes	unknown	
FTN	1228	FTT0782	FTL 1442	1298307	1297528	260	fabI	enoyl-ACP reductase I	fatty acids and lipids metabolism	unknown	
FTN	1229	FTT0781c	FTL 1443	1298595	1299305	237	-	conserved protein of unknown function	unknown function - conserved	unknown	
FTN	1230	(FTT1211c)	(5':722099-7224883':1371588-1371400)	1300131	1299559	191	-	protein of unknown function	unknown function - novel	cytoplasm	
FTN	1231	FTT1212c	FTL 0732	1300514	1300134	127	gloA	lactoylglutathione lyase	other metabolism - degradation, utilization, assimilation	unknown	
FTN	1232	FTT1213	FTL 0731	1300558	1301340	261	-	conserved hypothetical membrane protein	hypothetical - conserved	cytoplasmic membrane	
FTN	1233	FTT1214c	FTL 0730	1302386	1301598	263	-	haloacid dehalogenase-like hydrolase	putative enzymes	unknown	
FTN	1234	FTT1215c	FTL 0729	1303525	1302512	338	queA	S-adenosylmethionine:tRNA ribosyltransferase- isomerase	translation, ribosomal structure and biogenesis	cytoplasm	
FTN	1235	FTT1216c	FTL 0728	1304012	1303554	153	-	protein of unknown function	unknown function - novel	cytoplasm	
FTN	1236	FTT1217c	FTL 0727	1305269	1304037	411	ubiF	2-octaprenyl-3-methyl-6-methoxy-1,4-benzoquinol hydroxylase	cofactors, prosthetic groups, electron carriers metabolism	unknown	
FTN	1237	FTT1218c	FTL 0726	1306470	1305262	403	ubiH	2-octaprenyl-6-methoxyphenyl hydroxylase	cofactors, prosthetic groups, electron carriers metabolism	cytoplasmic membrane	
FTN	1238	FTT1219c	FTL 0725	1307129	1306482	216	-	conserved protein of unknown function	unknown function - conserved	unknown	
FTN	1239	FTT1220	FTL 0724	1307455	1308000	182	-	5-formyltetrahydrofolate cycloligase	putative enzymes	unknown	

FTN 1240	FTT1221	FTL 0723	1308006	1308269	88	-	BolA family protein	cofactors, prosthetic groups, electron carriers metabolism	unknown	
FTN 1241	FTT1222	FTL 0722	1308279	1308902	208	-	DedA family protein	putative enzymes	unknown	
FTN 1242	FTT1223	FTL 0721	1308961	1309605	215	-	DedA family protein	putative enzymes	cytoplasmic membrane	
FTN 1243	FTT1224c	FTL 0720	1310382	1309687	232	recO	RecFOR complex, RecO component	DNA replication, recombination, modification and repair - restriction/modification	cytoplasm	
FTN 1244	FTT1225c	FTL 0719	1310695	1310369	109	-	hypothetical protein	hypothetical - novel	cytoplasm	
FTN 1245	FTT1226c	FTL 0718	1311863	1310691	391	iscS	cysteine desulfurase	amino acid metabolism - biosynthesis	unknown	
FTN 1246	FTT1227	FTL 0717	1312113	1314767	885	rne	ribonuclease E	translation, ribosomal structure and biogenesis	unknown	
FTN 1247	FTT1228	FTL 0716	1314785	1315588	268	lgt	prolipoprotein diacylglyceryl transferase	cell wall / LPS / capsule	cytoplasmic membrane	
FTN 1248	FTT1229	FTL 0715	1315576	1316397	274	thyA	thymidylate synthase	nucleotides and nucleosides metabolism	unknown	
FTN 1249	FTT1230	FTL 0714	1316412	1317644	411	serA	D-3-phosphoglycerate dehydrogenase	amino acid metabolism - biosynthesis	unknown	
FTN 1250	FTT1231	FTL 0713	1317648	1318841	398	-	predicted ATPase of the PP-loop superfamily	cell cycle	cytoplasm	
FTN 1251	FTT1233c	(FTL_0711- FTL 0712)	1320288	1318855	478	-	proton-dependent oligopeptide transporter (POT) family protein, di- or tripeptide:H+ symporter	transport	cytoplasmic membrane	
FTN 1252	FTT1234	(FTL 0710)	1320488	1321630	381	-	choloylglycine hydrolase family protein	cell wall / LPS / capsule	unknown	
FTN 1253	FTT1235c	FTL 0709	1322698	1321637	354	lpcC	glycosyl transferase, group 1	cell wall / LPS / capsule	unknown	
FTN 1254	FTT1236	FTL 0708	1322868	1323953	362	-	protein of unknown function	unknown function - novel	unknown	
FTN 1255	FTT1237	FTL 0707	1323964	1324851	296	-	glycosyl transferase, family 8	cell wall / LPS / capsule	unknown	
FTN 1256	FTT1238c	(FTL_0706- FTL 0706)	1326250	1324883	456	-	membrane protein of unknown function	unknown function - novel	cytoplasmic membrane	
FTN 1257	FTT1239	FTL 0705	1326405	1327673	423	-	membrane protein of unknown function	unknown function - novel	cytoplasmic membrane	
FTN 1258	FTT1240c	FTL 0704	1328239	1327694	182	-	conserved protein of unknown function	unknown function - conserved	cytoplasm	
FTN 1259	FTT1241	FTL 0703	1328343	1329593	417	glyA	serine hydroxymethyltransferase	amino acid metabolism - biosynthesis	unknown	
FTN 1260	(FTT1242)	FTL 0702	1329874	1331325	484	-	hypothetical membrane protein	hypothetical - novel	outer membrane	
FTN 1261	Schunoseq	LVSnoseq	1331640	1333124	495	-	protein of unknown function	unknown function - novel	unknown	
FTN 1262	FTT1243c	FTL 0701	1334967	1333174	598	mreA	FAD binding family protein	energy metabolism	unknown	
FTN 1263	FTT1244c	FTL 0700	1335899	1335078	274	comL	competence lipoprotein ComL	transport	unknown	
FTN 1264	FTT1245	FTL 0699	1335988	1336983	332	rluD	ribosomal large subunit pseudouridine synthase D	translation, ribosomal structure and biogenesis	cytoplasm	
FTN 1265	FTT1246	FTL 0698	1336964	1337773	270	-	peptidoglycan hydrolase	cell wall / LPS / capsule	unknown	
FTN 1266	FTT1247	FTL 0697	1337851	1338948	366	-	ABC-type transport system permease protein	transport	unknown	
FTN 1267	FTT1248	FTL 0696	1338966	1339724	253	-	ATP-binding Cassette (ABC) superfamily protein	transport	unknown	
FTN 1268	FTT1249	FTL 0695	1339737	1340459	241	-	conserved protein of unknown function	unknown function - conserved	unknown	
FTN 1269	FTT1250	FTL 0694	1340467	1341162	232	-	protein of unknown function	unknown function - novel	unknown	
FTN 1270	FTT1251c	FTL 0693	1341841	1341158	228	-	conserved membrane protein of unknown function	unknown function - conserved	cytoplasmic membrane	
FTN 1271	FTT1252	FTL 0692	1341912	1342637	242	-	conserved protein of unknown function	unknown function - conserved	unknown	
FTN 1272	FTT1253	FTL 0691	1342876	1344357	494	-	proton-dependent oligopeptide transporter (POT) family protein, di- or tripeptide:H+ symporter	transport	cytoplasmic membrane	
FTN 1273	FTT1254	FTL 0690	1344468	1346156	563	-	long chain fatty acid CoA ligase	other metabolism - degradation, utilization, assimilation	unknown	
FTN 1274	FTT1255c	FTL 0689	1346974	1346150	275	-	transcriptional regulator, AraC family	signal transduction and regulation	unknown	
FTN 1275	FTT1256	FTL 0688	1347139	1348665	509	-	drug:H+ antiporter-1 (DHA2) family protein	transport - drugs / antibacterial compounds	cytoplasmic membrane	
FTN 1276	FTT1257	FTL 0687	1348690	1349712	341	-	membrane fusion protein	motility, attachment and secretion structure	periplasm	
FTN 1277	FTT1258	FTL 0686	1349725	1351194	490	-	outer membrane efflux protein	transport - drugs / antibacterial compounds	outer membrane	
FTN 1278	FTT1259	FTL 0685	1351324	1352070	249	nadE	NAD synthase	cofactors, prosthetic groups, electron carriers metabolism	unknown	
FTN 1279	FTT1260	FTL 0684	1352177	1352596	140	-	protein of unknown function	unknown function - novel	unknown	
FTN 1280	(FTT1261c)	(670424- 670997)	1353265	1352702	188	-	flavidoxin, related to tryptophan repressor binding protein	putative enzymes	unknown	yes
FTN 1281	FTT1266c	(FTL 1194)	1353978	1353283	232	-	pirin family protein	putative enzymes	unknown	
FTN 1282	FTT1267	(FTL 1193)	1354079	1354972	298	-	transcriptional regulator, LysR family	signal transduction and regulation	unknown	
FTN 1283	FTT1268c	FTL 1192	1356176	1354992	395	dnaJ	chaperone, DnaJ family, with C-terminal Zn finger domain	post-translational modification, protein turnover, chaperones	unknown	
FTN 1284	FTT1269c	FTL 1191	1358130	1356205	642	dnaK	chaperone, heat shock protein, HSP 70 family	post-translational modification, protein turnover, chaperones	unknown	
FTN 1285	FTT1270c	FTL 1190	1358818	1358234	195	grpE	chaperone GrpE (heat shock protein). Hsp70/Hsc70 protein regulator activity	post-translational modification, protein turnover, chaperones	unknown	
FTN 1286	FTT1271	FTL 1189	1358907	1360070	388	mltA	membrane-bound lytic murein transglycosylase	cell wall / LPS / capsule	unknown	
FTN 1287	FTT1272	FTL 1188	1360066	1361247	394	hemG	protoporphyrinogen oxidase	cofactors, prosthetic groups, electron carriers metabolism	unknown	
FTN 1288	FTT1273	FTL 1187	1361389	1361814	142	rplM	50S ribosomal protein L13	translation, ribosomal structure and biogenesis	unknown	
FTN 1289	FTT1274	FTL 1186	1361833	1362219	129	rpsI	30S ribosomoal protein S9	translation, ribosomal structure and biogenesis	unknown	
FTN 1290	FTT1275	FTL 1185	1362282	1362896	205	mglA	macrophage growth locus, protein A	signal transduction and regulation	unknown	
FTN 1291	FTT1276	FTL 1184	1362901	1363305	135	mglB	macrophage growth locus, subunit B	unknown function - conserved	unknown	
FTN 1292	FTT1277c	FTL 1183	1364576	1363308	423	-	solute:sodium symporter	transport	cytoplasmic membrane	
FTN 1293	FTT1278c	FTL 1182	1365134	1364580	185	rnhB	ribonuclease HII	DNA replication, recombination, modification and repair - restriction/modification	cytoplasm	
FTN 1294	FTT1279c	FTL 1181	1365879	1365139	247	-	rRNA methylase, SpoU family	translation, ribosomal structure and biogenesis	cytoplasm	
FTN 1295	FTT1280c	FTL 1180	1366327	1365875	151	ptsN	PEP-dependent sugar phosphotransferase system (PTS), enzyme IIA	transport - carbohydrates (sugars, polysaccharides)	unknown	
FTN 1296	FTT1281c	FTL 1179	1366660	1366367	98	-	sigma54 modulation protein	translation, ribosomal structure and biogenesis	cytoplasm	
FTN 1297	FTT1282	(FTL 1178)	1366779	1367021	81	-	protein of unknown function	unknown function - novel	unknown	
FTN 1298	FTT1283	FTL 1177	1367030	1368379	450	-	GTPase of unknown function	putative enzymes	unknown	
FTN 1299	FTT1284c	1128601- 1128792	1368586	1368395	64	-	hypothetical protein	hypothetical - novel	unknown	
FTN 1300	FTT1285c	(FTL_1176- 1128566)	1369497	1368610	296	-	transcriptional regulator, LysR family	signal transduction and regulation	unknown	
FTN 1301	(FTT1286)	(1126703- 1127545)	1369632	1370684	351	-	choloylglycine hydrolase family protein	cell wall / LPS / capsule	unknown	yes
FTN 1302	FTT1287	FTL 1174	1370906	1371826	307	cysK	cysteine synthase	amino acid metabolism - biosynthesis	unknown	
FTN 1303	FTT1288	FTL 1173	1372091	1372657	189	-	disulfide bond formation protein DsbB family	post-translational modification, protein turnover, chaperones - protein modification	cytoplasmic membrane	
FTN 1309	FTT1699	FTL 1172	1378294	1380753	820	pdpA	protein of unknown function	unknown function - novel	unknown	
FTN 1310	FTT1700	FTL 1171	1380761	1384039	1093	pdpB	protein of unknown function	unknown function - novel	outer membrane	
FTN 1311	FTT1701	FTL 1170	1384032	1384406	125	-	protein of unknown function	unknown function - novel	unknown	
FTN 1312	FTT1702	FTL 1169	1384422	1384913	164	-	conserved hypothetical protein	hypothetical - conserved	unknown	
FTN 1313	FTT1703	FTL 1168	1384921	1386648	576	-	hypothetical protein	hypothetical - novel	unknown	
FTN 1314	FTT1704	FTL 1167	1386652	1387170	173	-	conserved hypothetical protein	hypothetical - conserved	unknown	
FTN 1315	FTT1705	FTL 1166	1387186	1388613	476	-	protein of unknown function	unknown function - novel	unknown	
FTN 1316	FTT1706	FTL 1165	1388629	1389249	207	-	protein of unknown function	unknown function - novel	unknown	
FTN 1317	FTT1707	FTL 1164	1389274	1390422	383	-	protein of unknown function	unknown function - novel	unknown	
FTN 1318	FTT1708	FTL 1163	1390391	1391161	257	-	hypothetical protein	hypothetical - novel	unknown	
FTN 1319	FTT1709	FTL 1162	1391204	1395178	1325	pdpC	hypothetical protein	hypothetical - novel	unknown	
FTN 1320	FTT1710	FTL 1161	1395199	1395762	188	-	hypothetical protein	hypothetical - novel	unknown	
FTN 1321	FTT1711c	FTL 1160	1396968	1395775	398	iglD	intracellular growth locus protein D	unknown function - novel	unknown	
FTN 1322	FTT1712c	FTL 1159	1397618	1396992	209	iglC	intracellular growth locus protein C	unknown function - novel	unknown	
FTN 1323	FTT1713c	FTL 1158	1399155	1397638	506	iglB	intracellular growth locus protein B	unknown function - conserved	unknown	
FTN 1324	FTT1714c	FTL 1157	1399723	1399172	184	iglA	intracellular growth locus protein A	unknown function - conserved	unknown	
FTN 1325	FTT1715c	(FTL 1155)	1403540	1399806	1245	pdpD	protein of unknown function	unknown function - novel	outer membrane	
FTN 1326	(FTT1716c)	LVSnoseq	1404786	1403644	381	-	conserved protein of unknown function	unknown function - conserved	unknown	
(FTN_1327)	(FTT1362)	(1094548- 1094802)	1404933	1406114						
FTN 1328	(FTT1364)	(FTL 1151)	1406119	1407570	484	treA	trehalase	carbohydrate metabolism - degradation, utilization, assimilation	unknown	
FTN 1329	FTT1365c	FTL 1149	1408804	1407743	354	fbaA	fructose bisphosphate aldolase Class II	energy metabolism	unknown	
FTN 1330	FTT1366c	FTL 1148	1410256	1408823	478	pyk	pyruvate kinase	carbohydrate metabolism - degradation, utilization, assimilation	unknown	
FTN 1331	FTT1367c	FTL 1147	1411450	1410275	392	pgk	phosphoglycerate kinase	energy metabolism	unknown	
FTN 1332	FTT1368c	FTL 1146	1412470	1411472	333	gapA	glyceraldehyde-3-phosphate dehydrogenase/erythrose-4-phosphate dehydrogenase	energy metabolism	unknown	
FTN 1333	FTT1369c	FTL 1145	1414499	1412511	663	tktA	transketolase I	carbohydrate metabolism - degradation, utilization, assimilation	unknown	
FTN 1334	FTT1370	FTL 1144	1414639	1415139	167	-	conserved protein of unknown function	unknown function - conserved	unknown	
FTN 1335	FTT1371	FTL 1143	1415186	1415365	60	rpmF	50S ribosomal protein L32	translation, ribosomal structure and biogenesis	unknown	
FTN 1336	FTT1372	FTL 1142	1415467	1416510	348	plsX	Fatty acid/phospholipid biosynthesis enzyme	cell wall / LPS / capsule	unknown	
FTN 1337	FTT1373	FTL 1141	1416610	1417578	323	fabH	3-oxoacyl-[acyl-carrier protein] synthase III	fatty acids and lipids metabolism	unknown	
FTN 1338	FTT1374	FTL 1140	1417629	1418546	306	fabD	malonyl-CoA:ACP transacylase	fatty acids and lipids metabolism	unknown	

FTN_1339	FTT1375	FTL_1139	1418563	1419303	247	fabG	beta-ketoacyl-ACP reductase	fatty acids and lipids metabolism	unknown	
FTN_1340	FTT1376	FTL_1138	1419403	1419684	94	acpP	acyl carrier protein	cell wall / LPS / capsule	unknown	
FTN_1341	FTT1377	FTL_1137	1419767	1421023	419	fabF	beta-ketoacyl-ACP synthase II	fatty acids and lipids metabolism	unknown	
FTN_1342	(FTT1378)	FTL_1136	1421108	1421389	94	-	conserved hypothetical protein	hypothetical - conserved	unknown	
FTN_1343	(FTT1379c)	1078593-1079276	1422068	1421385	228	-	conserved protein of unknown function	unknown function - conserved	unknown	
FTN_1344	(FTT1380)	FTL_1134	1422190	1423386	399	-	major facilitator superfamily (MFS) transport protein	transport	cytoplasmic membrane	
FTN_1345	(FTT1381)	FTL_1133	1423503	1424552	350	-	conserved hypothetical membrane protein	hypothetical - conserved	cytoplasmic membrane	
FTN_1346	FTT1382	FTL_1132	1424642	1425427	262	-	inositol monophosphatase family protein.	putative enzymes	unknown	
FTN_1347	FTT1383	(FTL_1130-FTL_1131)	1425430	1426707	426	sun	tRNA and rRNA cytosine-C5-methylases, sun protein	translation, ribosomal structure and biogenesis	unknown	
FTN_1348	FTT1384c	FTL_1129	1427179	1426691	163	-	acetyltransferase	putative enzymes	unknown	
FTN_1349	FTT1385c	(FTL_0677-FTL_1128)	1427817	1427275	181	-	hypothetical protein	hypothetical - novel	unknown	
FTN_1350	FTT1387c	FTL_0676	1429850	1427817	678	ligA	DNA ligase	DNA replication, recombination, modification and repair - restriction/modification	cytoplasm	
FTN_1351	FTT1388	FTL_0675	1430157	1430870	238	-	conserved hypothetical protein	hypothetical - conserved	unknown	
FTN_1352	FTT1389	FTL_0674	1430875	1431669	265	panB	3-methyl-2-oxobutanoate hydroxymethyltransferase	cofactors, prosthetic groups, electron carriers metabolism	unknown	
FTN_1353	FTT1390	FTL_0673	1431659	1432441	261	panC	pantoate-beta-alanine ligase	cofactors, prosthetic groups, electron carriers metabolism	unknown	
FTN_1354	FTT1391	(FTL_0672)	1432438	1432770	111	panD	aspartate 1-decarboxylase	cofactors, prosthetic groups, electron carriers metabolism	unknown	
FTN_1355	FTT1392	FTL_0671	1432777	1433544	256	-	regulatory factor, Bvg accessory factor family	signal transduction and regulation	unknown	
FTN_1356	FTT1393c	FTL_0670	1435358	1433562	599	recD	exodeoxyribonuclease V, alpha subunit	DNA replication, recombination, modification and repair - restriction/modification	cytoplasm	
FTN_1357	FTT1394c	FTL_0669	1438974	1435327	1216	recB	ATP-dependent exoDNAse (exonuclease V) beta subunit	DNA replication, recombination, modification and repair - restriction/modification	cytoplasm	
FTN_1358	FTT1396c	FTL_0667	1439578	1439009	190	-	hypothetical membrane protein	hypothetical - novel	cytoplasmic membrane	
FTN_1359	FTT1397c	FTL_0666	1442759	1439523	1079	recC	exodeoxyribonuclease V, gamma subunit	DNA replication, recombination, modification and repair - restriction/modification	cytoplasm	
FTN_1360	(FTT1398c)	FTL_0665	1444422	1442839	528	gltB	glutamate synthase domain 2	other metabolism - degradation, utilization, assimilation	unknown	
FTN_1361	FTT1399	FTL_0664	1444600	1445466	289	-	drug/metabolite exporter	transport - drugs / antibacterial compounds	unknown	
FTN_1362	FTT1400c	FTL_0663	1445869	1445486	128	-	hypothetical protein	hypothetical - novel	unknown	
FTN_1363	FTT1401	FTL_0662	1446038	1446688	217	-	prophage repressor protein	signal transduction and regulation	unknown	
FTN_1367	FTT1402c	FTL_0661	1448899	1447268	544	-	protein of unknown function	unknown function - novel	unknown	
FTN_1368	FTT1403c	FTL_0660	1449140	1448919	74	feoA	Fe2+ transport system protein A	transport	unknown	
FTN_1369	FTT1404	FTL_0659	1449249	1449953	235	-	protein of unknown function	unknown function - novel	unknown	
FTN_1370	FTT1405c	FTL_0657	1450792	1450037	252	gloB	hydroxyacylglutathione hydrolase	other metabolism - degradation, utilization, assimilation	unknown	
FTN_1371	FTT1406c	FTL_0656	1451963	1450809	385	-	conserved protein of unknown function	unknown function - conserved	unknown	
FTN_1372	FTT1407c	FTL_0655	1453045	1451963	361	-	protein of unknown function	unknown function - novel	unknown	
FTN_1373	FTT1408c	FTL_0654	1453760	1453014	249	hemD	uroporphyrinogen-III synthase	cofactors, prosthetic groups, electron carriers metabolism	unknown	
FTN_1374	FTT1409c	FTL_0653	1454180	1453764	139	sufE	sulfur acceptor protein SufE	other metabolism - biosynthesis	unknown	
FTN_1376	FTT1410	FTL_0652	1454884	1455417	178	-	disulfide bond formation protein, DsbB family	post-translational modification, protein turnover, chaperones - protein modification	cytoplasmic membrane	
FTN_1377	FTT1412	FTL_0650	1455684	1457378	565	proS	prolyl-tRNA synthetase	other metabolism - biosynthesis	unknown	
FTN_1378	Schunoseq	LVSnoseq	1457390	1457926	179	-	hypothetical protein	hypothetical - novel	unknown	
(FTN_1379)	Schunoseq	LVSnoseq	1459691	1457975						
FTN_1380	FTT1413	(FTL_0648-FTL_0649)	1459763	1460893	377	-	aspartate/tyrosine/aromatic aminotransferase	amino acid metabolism - biosynthesis	unknown	
FTN_1381	(FTT1414-FTT1415)	(FTL_0646-FTL_0647)	1460964	1462379	472	-	hypothetical protein	hypothetical - novel	unknown	yes
FTN_1382	FTT1416c	FTL_0645	1462831	1462421	137	-	protein of unknown function	unknown function - novel	unknown	
FTN_1383	FTT1417	FTL_0644	1463001	1463768	256	-	carbon-nitrogen hydrolase	putative enzymes	unknown	
FTN_1384	FTT1418c	FTL_0643	1464180	1463758	141	nusB	transcription termination factor	transcription	unknown	
FTN_1385	(FTT1419)	FTL_0642	1464367	1464765	133	-	protein of unknown function	unknown function - novel	unknown	
FTN_1386	FTT1420	(FTL_0641)	1464785	1465294	170	-	protein of unknown function	unknown function - novel	unknown	
FTN_1387	FTT1421	FTL_0640	1465519	1465962	148	-	conserved protein of unknown function	unknown function - conserved	cytoplasm	
FTN_1388	FTT1422	FTL_0639	1466005	1466622	206	-	oxidoreductase	putative enzymes	unknown	
FTN_1389	FTT1423c	FTL_0638	1467367	1466621	249	-	conserved hypothetical membrane protein	hypothetical - novel	cytoplasmic membrane	
FTN_1390	FTT1424c	FTL_0637	1468055	1467372	228	-	Zn-dependent hydrolase	putative enzymes	unknown	
FTN_1391	FTT1425c	(FTL_0634-FTL_0636)	1469791	1468103	563	naoX	uncharacterized NAD(FAD)-dependent dehydrogenase	putative enzymes	unknown	
FTN_1392	FTT1426c	FTL_0633	1470334	1469819	172	-	rhodanese-related sulfurtransferase	other metabolism - biosynthesis	unknown	
FTN_1394	Schunoseq	LVSnoseq	1471791	1471522	90	-	hypothetical protein	hypothetical - novel	unknown	
FTN_1395	Schunoseq	LVSnoseq	1472776	1471775	334	-	conserved hypothetical protein	hypothetical - conserved	cytoplasm	
FTN_1396	Schunoseq	LVSnoseq	1473271	1472783	163	-	RecB family exonuclease	DNA replication, recombination, modification and repair	unknown	
FTN_1397	Schunoseq	LVSnoseq	1477344	1473445	1300	-	protein of unknown function	unknown function - novel	unknown	
FTN_1398	FTT1428c	(618666-619123)	1477947	1477435	171	-	acetyltransferase	putative enzymes	unknown	
FTN_1399	(FTT1429c)	617891-618604	1478668	1477955	238	-	hypothetical protein	hypothetical - novel	unknown	
FTN_1400	(FTT1430c)	617192-617878	1479367	1478681	229	-	S-adenosylmethionine-dependent methyltransferase	putative enzymes	unknown	
FTN_1401	FTT1431	FTL_0627	1479437	1480069	211	rhtB	homoserine/threonine efflux family protein	transport - amino-acid	unknown	
FTN_1402	FTT1432c	FTL_0626	1480959	1480072	296	ppnK	NAD kinase	carbohydrate metabolism	unknown	
FTN_1403	FTT1433	FTL_0625	1481150	1482091	314	gtrB	glycosyl transferase	cell wall / LPS / capsule	unknown	
FTN_1404	FTT1434c	FTL_0624	1482877	1482101	259	-	ATP-binding cassette (ABC) superfamily protein	transport	unknown	
FTN_1405	FTT1435c	FTL_0623	1483809	1482889	307	-	ATP-binding cassette (ABC) superfamily protein	transport	unknown	
FTN_1406	FTT1437c	(610691-611788)fusion	1484686	1483913	258	-	conserved hypothetical membrane protein	hypothetical - conserved	cytoplasmic membrane	
FTN_1407	FTT1438c	(610691-611788)fusion	1485012	1484689	108	-	conserved hypothetical protein	hypothetical - conserved	unknown	
FTN_1408	FTT1439c	FTL_0620	1485905	1485141	255	-	Mg-dependent DNase	DNA replication, recombination, modification and repair	unknown	
FTN_1409	(FTT1440c)	(608331-609712)	1487373	1485991	461	-	major facilitator superfamily (MFS) transport protein	transport	cytoplasmic membrane	
FTN_1410	FTT1441	FTL_0617	1487622	1488059	146	bfr	bacterioferritin	energy metabolism	unknown	
FTN_1412	FTT1442c	FTL_0616	1489635	1488682	318	-	DNA-directed RNA polymerase, alpha subunit/40 kD subunit	transcription	cytoplasm	
FTN_1413	(FTT1443c)	(FTL_0615)	1490961	1489726	412	-	ATPase, AAA family, related to the helicase subunit of the Holliday junction resolvase	DNA replication, recombination, modification and repair	unknown	
FTN_1414	FTT1444c	FTL_0612	1492131	1491208	308	ppx	exopolyphosphatase	other metabolism - biosynthesis	unknown	
FTN_1415	FTT1445	FTL_0611	1492329	1492649	107	-	thioredoxin	cofactors, prosthetic groups, electron carriers metabolism	unknown	
FTN_1416	FTT1446	FTL_0610	1492695	1493954	420	rho	transcription termination factor Rho	transcription	unknown	
FTN_1417	FTT1447c	FTL_0609	1495781	1494300	494	manB	phosphomannomutase	carbohydrate metabolism - biosynthesis	unknown	
FTN_1418	FTT1448c	FTL_0608	1497217	1495814	468	manC	mannose-1-phosphate guanylyltransferase	cell wall / LPS / capsule	unknown	
(FTN_1419)	FTT1450c	FTL_0606	1497548	1497892						
FTN_1420	Schunoseq	LVSnoseq	1499933	1498689	415	wzx	O antigen flippase	transport	cytoplasmic membrane	
FTN_1421	FTT1456c	FTL_0600	1501830	1499941	630	wbtH	glutamine amidotransferase/asparagine synthase	amino acid metabolism - biosynthesis	unknown	
FTN_1422	Schunoseq	LVSnoseq	1502944	1501835	370	wbtN	glycosyl transferase, group 1	cell wall / LPS / capsule	unknown	
FTN_1423	FTT1457c	FTL_0599	1504025	1502940	362	wbtG	glycosyl transferase, group 1	cell wall / LPS / capsule	unknown	
FTN_1424	Schunoseq	LVSnoseq	1505283	1503994	430	-	hypothetical membrane protein	hypothetical - novel	cytoplasmic membrane	
FTN_1425	FTT1459c	FTL_0597	1506254	1505283	324	wbtF	NAD dependent epimerase	cell wall / LPS / capsule	unknown	
FTN_1426	FTT1460c	FTL_0596	1507564	1506257	436	wbtE	UDP-glucose/GDP-mannose dehydrogenase family protein	cell wall / LPS / capsule	unknown	
FTN_1427	FTT1461c	FTL_0595	1508659	1507571	363	wbtD	glycosyl transferase, group 1	cell wall / LPS / capsule	unknown	
FTN_1428	Schunoseq	LVSnoseq	1509267	1508659	203	wbtO	transferase	cell wall / LPS / capsule	unknown	
FTN_1429	FTT1463c	FTL_0593	1509886	1509260	209	wbtP	galactosyl transferase	cell wall / LPS / capsule	unknown	
FTN_1430	FTT1455c	FTL_0601	1510991	1509879	371	wbtQ	aminotransferase	putative enzymes	unknown	
FTN_1431	FTT1464c	FTL_0592	1512733	1511000	578	wbtA	dTDP-glucose 4,6-dehydratase	cell wall / LPS / capsule	unknown	
FTN_1432	FTT1524c	FTL_0590	1517211	1513195	1339	hrpA	HrpA-like helicase	DNA replication, recombination, modification and repair	unknown	
FTN_1433	FTT1525c	FTL_0589	1518122	1517238	295	-	protein of unknown function	unknown function - novel	unknown	
FTN_1434	FTT1526c	FTL_0588	1520352	1518139	738	icd	isocitrate dehydrogenase	energy metabolism	unknown	
FTN_1435	(FTT1527c)	FTL_0587	1520820	1520404	139	-	arsenate reductase	cofactors, prosthetic groups, electron carriers metabolism	unknown	
FTN_1436	FTT1528	FTL_0586	1520964	1522649	562	fadD	long chain fatty acid CoA ligase	other metabolism - degradation, utilization, assimilation	unknown	
FTN_1437	FTT1529	FTL_0585	1522690	1524927	746	fadE	Acyl-CoA dehydrogenase	other metabolism - degradation, utilization, assimilation	unknown	

FTN 1438	FTT1530	FTL 0584	1525003	1527696	898	-	bifunctional protein: 3-hydroxacyl-CoA dehydrogenase/acyl-CoA-binding protein	other metabolism - degradation, utilization, assimilation	unknown	
FTN 1439	FTT1531	FTL 0583	1527712	1528896	395	fadA	acetyl-CoA acetyltransferase	other metabolism - degradation, utilization, assimilation	unknown	
FTN 1440	FTT1532	FTL 0582	1528961	1529446	162	-	conserved hypothetical protein, Thioesterase superfamily	hypothetical - conserved	unknown	
FTN 1441	(FTT1533c)	FTL 0581	1530722	1529445	426	-	sugar porter (SP) family protein	transport - carbohydrates (sugars, polysaccharides)	cytoplasmic membrane	
FTN 1442	(1595356- 1595572)	FTL 0580	1531099	1530875	75	-	conserved protein of unknown function	unknown function - conserved	unknown	
FTN 1443	FTT1534c	FTL 0579	1532599	1531187	471	-	nicotinic acid phosphoribosyltransferase	other metabolism - biosynthesis	unknown	
FTN 1444	(FTT1535c)	(556167- FTL 0578)	1533686	1532673	338	-	ornithine cyclodeaminase, mu-crystallin homolog	amino acid metabolism - degradation, utilization, assimilation	unknown	yes
FTN 1445	FTT1536c	(FTL_0576- FTL 0577)	1534848	1533937	304	-	conserved protein of unknown function	unknown function - conserved	unknown	
FTN 1446	FTT1537c	FTL 0574	1536382	1534991	464	-	conserved hypothetical protein	hypothetical - conserved	unknown	
FTN 1447	FTT1538c	FTL 0573	1537371	1536391	327	-	conserved protein of unknown function	unknown function - conserved	unknown	
FTN 1448	FTT1539c	FTL 0572	1538812	1537385	476	-	protein of unknown function	unknown function - novel	unknown	
FTN 1449	FTT1540c	FTL 0571	1539477	1538872	202	-	conserved protein of unknown function	cell wall / LPS / capsule	unknown	
FTN 1450	(FTT1541c)	FTL 0570	1540053	1539655	133	-	hypothetical membrane protein	hypothetical - novel	cytoplasmic membrane	
FTN 1451	FTT1542c	FTL 0569	1540707	1540174	178	-	protein of unknown function	unknown function - novel	unknown	
FTN 1452	FTT1543	(546773- 547286)	1540850	1541536	229	-	two-component response regulator	signal transduction and regulation	cytoplasm	
FTN 1453	(FTT1544)	(545603- 546274)	1541523	1542557	345	-	two-component regulator, sensor histidine kinase	signal transduction and regulation	cytoplasmic membrane	
FTN 1454	(FTT1545)	(544459- 545511)	1542637	1543887	417	-	NAD/FAD-binding protein	putative enzymes	unknown	
FTN 1455	(FTT1546)	(543959- 544462)	1543884	1544657	258	-	conserved hypothetical protein	hypothetical - conserved	unknown	yes
FTN 1456	(FTT1547)	(FTL 0561)	1544780	1545940	387	cfa	cyclopropane fatty acid synthase, methyltransferase	fatty acids and lipids metabolism	unknown	
FTN 1457	FTT1549	FTL 0560	1545936	1546451	172	-	protein of unknown function	unknown function - novel	unknown	
FTN 1458	FTT1550	FTL 0559	1546487	1546990	168	-	conserved hypothetical protein	hypothetical - conserved	unknown	
FTN 1459	FTT1551	FTL 0558	1546995	1547735	247	-	short chain dehydrogenase	putative enzymes	unknown	
FTN 1460	FTT1552	(FTL 0557)	1547752	1548579	276	-	delta 9 acyl-lipid fatty acid desaturase	fatty acids and lipids metabolism	unknown	
FTN 1461	FTT1553c	FTL 0556	1550903	1548609	765	rnr	ribonuclease R	transcription	unknown	
FTN 1462	FTT1554c	FTL 0555	1551804	1550899	302	truB	tRNA pseudouridine synthase B	translation, ribosomal structure and biogenesis	unknown	
FTN 1463	FTT1555c	FTL 0554	1552483	1551794	230	rnc	ribonuclease III	transcription	unknown	
FTN 1464	FTT1556c	FTL 0553	1553353	1552493	287	lepB	signal peptidase I	motility, attachment and secretion structure	unknown	
FTN 1465	FTT1557c	FTL 0552	1554044	1553361	228	-	two-component response regulator	signal transduction and regulation	unknown	
FTN 1466	(FTT1558c)	(532915- 533882)	1555576	1554488	363	-	conserved protein of unknown function	unknown function - conserved	unknown	
FTN 1467	FTT1559c	FTL 0549	1556637	1555816	274	proC	pyrroline-5-carboxylate reductase	amino acid metabolism - biosynthesis	unknown	
FTN 1468	FTT1560	FTL 0548	1556744	1557319	192	-	HAM1-like protein, possible xanthosine triphosphate pyrophosphatase	putative enzymes	unknown	
FTN 1469	FTT1561	FTL 0547	1557427	1558719	431	kdtA	3-deoxy-D-manno-octulosonic-acid transferase	fatty acids and lipids metabolism	unknown	
FTN 1470	FTT1562	FTL 0546	1558719	1559597	293	ispA	geranyl diphosphate synthase/farnesyl diphosphate synthase	cofactors, prosthetic groups, electron carriers metabolism	unknown	
FTN 1471	FTT1563	FTL 0545	1559648	1560409	254	pcs	(CDP-alcohol) phosphatidyltransferase	fatty acids and lipids metabolism	unknown	
FTN 1472	FTT1564	FTL 0544	1560482	1561288	269	-	conserved protein of unknown function	unknown function - conserved	unknown	
FTN 1474	(FTT1565c)	(525666- 526706)	1562807	1561674	378	bglX	glycosyl hydrolase family 3	carbohydrate metabolism - degradation, utilization, assimilation	unknown	yes
FTN 1475	(FTT1566c)	FTL 0542	1563483	1563112	124	-	protein of unknown function	unknown function - novel	unknown	
FTN 1476	FTT1567c	FTL 0541	1564161	1563577	195	-	protein of unknown function	unknown function - novel	unknown	
FTN 1477	FTT1568c	FTL 0540	1565363	1564224	380	lpxB	lipid A disaccharide synthetase	fatty acids and lipids metabolism	unknown	
FTN 1478	FTT1569c	FTL 0539	1566142	1565366	259	lpxA	UDP-N-acetylglucosamine acyltransferase	fatty acids and lipids metabolism	unknown	
FTN 1479	FTT1570c	FTL 0538	1566636	1566148	163	fabZ	beta-hydroxyacyl-ACP dehydratase	fatty acids and lipids metabolism	unknown	
FTN 1480	FTT1571c	FTL 0537	1567664	1566654	337	lpxD	UDP-3-O-(3-hydroxy-fatty acid)-glucosamine N-acyltransferase	fatty acids and lipids metabolism	unknown	
FTN 1481	FTT1572c	FTL 0536	1568186	1567686	167	ompH	outer membrane protein	unknown function - conserved	unknown	
FTN 1482	FTT1573c	FTL 0535	1570700	1568325	792	-	conserved outer membrane protein of unknown function	unknown function - conserved	unknown	
FTN 1483	FTT1574c	FTL 0534	1571863	1570709	385	dxr	1-deoxy-D-xylulose 5-phosphate reductoisomerase	cofactors, prosthetic groups, electron carriers metabolism	unknown	
FTN 1484	FTT1575c	FTL 0533	1574475	1571872	868	gyrA	DNA gyrase, subunit A	DNA replication, recombination, modification and repair - restriction/modification	cytoplasm	
FTN 1485	(FTT1576- FTT1577)	(FTL_0530- FTL 0532)	1574733	1576397	555	cbpA	chitin-binding protein	putative enzymes	unknown	yes
FTN 1486	FTT1578c	FTL 0529	1577096	1576437	220	ung	uracil DNA glycosylase	DNA replication, recombination, modification and repair - repair	unknown	
FTN 1487	FTT1579c	FTL 0528	1579960	1577096	955	-	restriction endonuclease	DNA replication, recombination, modification and repair - restriction/modification	unknown	
FTN 1488	Schunoseq	LVSnoseq	1580906	1579953	318	-	prophage maintenance system killer protein (DOC)	mobile and extrachromosomal element functions - phage or plasmid related proteins	unknown	
FTN 1489	Schunoseq	LVSnoseq	1581764	1581021	248	-	protein of unknown function	unknown function - novel	outer membrane	
FTN 1490	Schunoseq	LVSnoseq	1582627	1581779	283	-	protein of unknown function	unknown function - novel	cytoplasm	
FTN 1491	Schunoseq	LVSnoseq	1584609	1582630	660	-	adenine specific DNA methylase	DNA replication, recombination, modification and repair - restriction/modification	unknown	
FTN 1492	FTT1483c	FTL 0311	1586203	1584794	470	lpdA	pyruvate dehydrogenase complex, E3 component, dihydrolipoamide dehydrogenase	other metabolism - degradation, utilization, assimilation	cytoplasm	
FTN 1493	FTT1484c	FTL 0310	1588112	1586220	631	aceF	pyruvate dehydrogenase complex, E2 component, dihydrolipoamide acyltransferase	carbohydrate metabolism - degradation, utilization, assimilation	unknown	
FTN 1494	FTT1485c	FTL 0309	1590811	1588133	893	aceE	pyruvate dehydrogenase complex, E1 component, pyruvate dehydrogenase	carbohydrate metabolism - degradation, utilization, assimilation	cytoplasm	
FTN 1495	FTT1486c	FTL 0308	1592298	1591114	395	-	conserved protein of unknown function	unknown function - conserved	cytoplasm	
FTN 1496	FTT1487	FTL 0307	1592378	1592989	204	coaE	dephospho-CoA kinase	cofactors, prosthetic groups, electron carriers metabolism	unknown	
FTN 1499	FTT1488	FTL 0306	1593289	1594290	334	trpS	tryptophanyl-tRNA synthetase	other metabolism - biosynthesis	unknown	
FTN 1500	FTT1489	FTL 0305	1594295	1595017	241	-	protein of unknown function	unknown function - novel	unknown	
FTN 1501	FTT1490	FTL 0304	1595030	1596325	432	-	monovalent cation:proton antiporter-1	transport	cytoplasmic membrane	
FTN 1502	(FTT1491c- FTT1492c)	(FTL_0301- FTL 0303)	1597764	1596730	345	-	hypothetical protein	hypothetical - novel	unknown	
FTN 1503	FTT1493c	FTL 0300	1598656	1598264	131	-	protein of unknown function	unknown function - novel	unknown	
FTN 1504	FTT1494c	FTL 0299	1599542	1598661	294	-	glucokinase regulatory protein	signal transduction and regulation	unknown	
FTN 1505	FTT1495c	FTL 0298	1601503	1599542	654	-	protein of unknown function	unknown function - novel	unknown	
FTN 1506	FTT1496c	FTL 0297	1602525	1601515	337	-	pilus assembly protein	motility, attachment and secretion structure	unknown	
FTN 1507	FTT1497c	FTL 0296	1603009	1602509	167	-	pilus assembly protein	motility, attachment and secretion structure	cytoplasm	
FTN 1508	FTT1498c	FTL 0295	1603976	1603032	315	accA	acetyl-CoA carboxylase, carboxytransferase subunit alpha	fatty acids and lipids metabolism	unknown	
FTN 1509	FTT1499	FTL 0294	1604143	1606674	844	mutS	MutS, subunit of MutHLS complex, methyl-directed mismatch repair protein	DNA replication, recombination, modification and repair - restriction/modification	cytoplasm	
FTN 1510	FTT1500	FTL 0293	1606754	1607194	147	secB2	preprotein translocase, subunit B	motility, attachment and secretion structure	unknown	
FTN 1511	(FTT1501)	(276927- 277928)	1607392	1608612	407	-	conserved hypothetical protein	hypothetical - conserved	unknown	
FTN 1512	FTT1502	FTL 0291	1608678	1609847	390	-	hydroxy/aromatic amino acid permease (HAAAP) family protein	transport - amino-acid	cytoplasmic membrane	
FTN 1513	FTT1503	FTL 0290	1609860	1610735	292	xerC	site-specific recombinase	DNA replication, recombination, modification and repair	unknown	
FTN 1514	FTT1504	FTL 0289	1610738	1611460	241	-	conserved hypothetical protein	hypothetical - conserved	unknown	
FTN 1515	FTT1505c	FTL 0288	1612084	1611473	204	-	hypothetical membrane protein	hypothetical - novel	cytoplasmic membrane	
FTN 1516	FTT1506	FTL 0287	1612172	1612747	192	-	protein of unknown function	unknown function - novel	unknown	
FTN 1517	FTT1507	FTL 0286	1612827	1613456	210	-	protein of unknown function	unknown function - novel	unknown	
FTN 1518	FTT1508c	FTL 0285	1616447	1614441	669	relA	GDP pyrophosphokinase/GTP pyrophosphokinase	other metabolism - biosynthesis	unknown	
FTN 1519	FTT1509c	FTL 0284	1617106	1616753	118	-	protein of unknown function	unknown function - novel	unknown	
FTN 1520	FTT1510c	FTL 0283	1618336	1617143	398	-	hydroxy/aromatic amino acid permease (HAAAP) family protein	transport - amino-acid	cytoplasmic membrane	
FTN 1521	FTT1511	FTL 0282	1618541	1619422	294	-	10 TMS drug/metabolite exporter protein	transport - drugs / antibacterial compounds	cytoplasmic membrane	
FTN 1522	FTT1512c	FTL 0281	1620341	1619418	308	-	subunit of DnaJ/DnaK/GrpE: chaperone with DnaK; heat shock protein	post-translational modification, protein turnover, chaperones - chaperones	unknown	
FTN 1523	(FTT1513)	FTL 0280	1620541	1621251	237	-	amino acid-polyamine-organocation family protein	transport - amino-acid	unknown	
FTN 1524	FTT1514c	(FTL 0279)	1621652	1621272	127	-	hypothetical membrane protein	hypothetical - novel	cytoplasmic membrane	
FTN 1525	FTT1515c	FTL 0278	1622248	1621652	199	-	phosphatidyltransferase	fatty acids and lipids metabolism	unknown	
FTN 1526	(FTT1516c)	FTL 0277	1623663	1622248	472	-	dihydrolipoamide dehydrogenase	amino acid metabolism - biosynthesis	unknown	
FTN 1527	FTT1517c	FTL 0276	1624385	1623669	239	-	conserved hypothetical membrane protein	hypothetical - conserved	cytoplasmic membrane	
FTN 1528	FTT1518	FTL 0275	1624415	1624975	187	-	methylated DNA-protein cysteine methyltransferase	DNA replication, recombination, modification and repair - repair	unknown	
FTN 1529	FTT1520c	FTL 0273	1626783	1625383	467	-	glutamate:GABA antiporter (APC family) protein	transport - amino-acid	cytoplasmic membrane	
FTN 1530	(FTT1521c)	FTL 0272	1627979	1626798	394	lysA	diaminopimelate decarboxylase	amino acid metabolism - biosynthesis	unknown	
FTN 1531	(FTT1522c)	(FTL 0271)	1628675	1627995	227	-	conserved protein of unknown function	unknown function - conserved	unknown	

FTN_1532	FTT0380c	FTL_0269	1629170	1630516	449	gdhA	glutamate dehydrogenase (NADP+)	amino acid metabolism - biosynthesis	unknown	
FTN_1533	(FTT0358)	(FTL_1291-FTL_1292)	1630614	1631801	396	-	conserved protein of unknown function	unknown function - conserved	unknown	yes
FTN_1534	FTT0359	FTL_1293	1631854	1632321	156	-	conserved protein of unknown function	unknown function - conserved	cytoplasm	
FTN_1535	FTT0360	FTL_1294	1632461	1633282	274	-	short chain dehydrogenase	putative enzymes	unknown	
FTN_1536	FTT0361c	(FTL_1295-FTL_1296)	1634656	1633310	449	-	amino acid-polyamine-organocation (APC) superfamily protein	transport - amino-acid	cytoplasmic membrane	
FTN_1537	FTT0362c	FTL_1297	1636650	1635781	290	-	hypothetical protein	hypothetical - novel	unknown	
FTN_1538	FTT1696	FTL_1714	1638515	1636884	544	groEL	chaperonin GroEL (HSP60 family)	post-translational modification, protein turnover, chaperones - chaperones	unknown	
FTN_1539	FTT1695	FTL_1715	1638840	1638556	95	groES	co-chaperonin GroES (HSP10)	post-translational modification, protein turnover, chaperones - chaperones	unknown	
FTN_1540	FTT1694c	FTL_1716	1639091	1639522	144	-	conserved hypothetical membrane protein	hypothetical - conserved	cytoplasmic membrane	
FTN_1542	FTT1693c	FTL_1717	1640166	1640927	254	-	conserved protein of unknown function	unknown function - conserved	unknown	
FTN_1543	(FTT0170c)	FTL_1719	1640934	1641398	155	-	conserved protein of unknown function	unknown function - conserved	unknown	
FTN_1544	FTT0169	FTL_1720	1642246	1641395	284	hemK	modification methylase, HemK family	translation, ribosomal structure and biogenesis	unknown	
FTN_1545	FTT0168	FTL_1721	1643324	1642242	361	prfA	protein chain release factor A	translation, ribosomal structure and biogenesis	unknown	
FTN_1546	FTT0167	FTL_1722	1644569	1643328	414	hemA	glutamyl-tRNA reductase	cofactors, prosthetic groups, electron carriers metabolism	unknown	
FTN_1547	FTT0166c	FTL_1723	1644749	1645384	212	-	conserved protein of unknown function	unknown function - conserved	unknown	
FTN_1548	FTT0165c	(FTL_1724-FTL_1724)	1645390	1646757	456	-	conserved protein of unknown function	unknown function - conserved	unknown	
FTN_1549	FTT0164c	FTL_1725	1646760	1647938	393	-	drug:H+ antiporter-1 (DHA1) family protein	transport - drugs / antibacterial compounds	cytoplasmic membrane	
FTN_1550	FTT0163c	FTL_1726	1648031	1649911	627	parE	topoisomerase IV, subunit B	DNA replication, recombination, modification and repair - restriction/modification	cytoplasm	
FTN_1551	FTT0162	FTL_1727	1650487	1649966	174	ampD	N-acetylmuramoyl-L-alanine amidase	carbohydrate metabolism - degradation, utilization, assimilation	unknown	
FTN_1552	FTT0161c	FTL_1728	1650561	1651184	208	-	acid phosphatase, PAP2 family	putative enzymes	cytoplasmic membrane	
FTN_1553	FTT0160	FTL_1729	1651661	1651200	154	nudH	dGTP pyrophosphohydrolase	DNA replication, recombination, modification and repair - restriction/modification	cytoplasm	
FTN_1554	(FTT0158c-FTT0159c)	(1661291-FTL_1730)	1651824	1653341	506	-	hypothetical membrane protein	hypothetical - novel	cytoplasmic membrane	
FTN_1555	FTT0157c	FTL_1731	1653462	1654343	294	-	licB-like transmembrane protein	putative enzymes	cytoplasmic membrane	
FTN_1556	FTT0156	FTL_1732	1654927	1654346	194	-	acid phosphatase, HAD superfamily protein	putative enzymes	unknown	
FTN_1557	FTT0155	FTL_1733	1655777	1654935	281	-	oxidoreductase iron/ascorbate family protein	other metabolism - degradation, utilization, assimilation	unknown	
FTN_1558	FTT0154	FTL_1734	1656778	1655903	292	xerD	site-specific recombinase	DNA replication, recombination, modification and repair	unknown	
FTN_1559	FTT0153	FTL_1735	1657291	1656947	115	rplS	50S ribosomal protein L19	translation, ribosomal structure and biogenesis	unknown	
FTN_1560	FTT0152	FTL_1736	1658049	1657294	252	trmD	tRNA (Guanine-N(1)-)-methyltransferase	translation, ribosomal structure and biogenesis	unknown	
FTN_1561	FTT0151	FTL_1737	1658963	1658457	169	rimM	16S rRNA processing protein RimM	translation, ribosomal structure and biogenesis	unknown	
FTN_1562	FTT0150	FTL_1738	1659255	1659010	82	rpsP	30S ribosomal protein S16	translation, ribosomal structure and biogenesis	unknown	
FTN_1563	FTT0149c	FTL_1739	1659549	1660706	386	metK	S-adenosylmethionine synthetase	amino acid metabolism - biosynthesis	unknown	
FTN_1564	FTT0148	FTL_1740	1662086	1660923	388	-	fatty acid desaturase	fatty acids and lipids metabolism	unknown	
FTN_1565	FTT0147	FTL_1741	1663210	1662203	336	gcp	O-sialoglycoprotein endopeptidase	post-translational modification, protein turnover, chaperones	unknown	
FTN_1566	FTT0146	FTL_1742	1663446	1663213	78	-	hypothetical membrane protein	hypothetical - novel	unknown	
FTN_1567	FTT0145	FTL_1743	1667802	1663552	1417	rpoC	DNA-directed RNA polymerase, beta' subunit/160 kD subunit	transcription	unknown	
FTN_1568	FTT0144	FTL_1744	1671940	1667867	1358	rpoB	DNA-directed RNA polymerase, beta subunit/140 kD subunit	transcription	cytoplasm	
FTN_1569	FTT0143	FTL_1745	1672471	1672097	125	rplL	50S ribosomal protein L7/L12	translation, ribosomal structure and biogenesis	unknown	
FTN_1570	FTT0142	FTL_1746	1673052	1672537	172	rplJ	50S ribosomal protein L10	translation, ribosomal structure and biogenesis	unknown	
FTN_1571	FTT0141	FTL_1747	1673917	1673225	231	rplA	50S ribosomal protein L1	translation, ribosomal structure and biogenesis	cytoplasm	
FTN_1572	FTT0140	FTL_1748	1674354	1673923	144	rplK	50S ribosomal protein L11	translation, ribosomal structure and biogenesis	cytoplasm	
FTN_1573	FTT0139	FTL_1749	1674956	1674426	177	nusG	transcription antitermination protein nusG	transcription	cytoplasm	
FTN_1574	FTT0138	FTL_1750	1675442	1674975	156	secE	preprotein translocase, subunit E, membrane protein	motility, attachment and secretion structure	cytoplasmic membrane	
FTN_1576	FTT0137	FTL_1751	1676818	1675637	394	tufA	elongation factor Tu	translation, ribosomal structure and biogenesis	cytoplasm	
FTN_1580	FTT0136	(FTL_1752)	1678698	1677286	471	-	DNA helicase	DNA replication, recombination, modification and repair	unknown	
FTN_1581	(FTT0135)	FTL_1753	1679195	1678701	165	-	small conductance mechanosensitive ion channel (MscS) family protein	transport	cytoplasmic membrane	
FTN_1582	FTT0134	FTL_1754	1679886	1679176	237	-	hypothetical membrane protein	hypothetical - novel	cytoplasmic membrane	
FTN_1583	FTT0133	FTL_1755	1680654	1679893	254	glpF	glycerol uptake facilitator protein	transport - carbohydrates (sugars, polysaccharides)	unknown	
FTN_1584	FTT0132	FTL_1756	1682196	1680667	510	glpD	glycerol-3-phosphate dehydrogenase	other metabolism - degradation, utilization, assimilation	unknown	
FTN_1585	FTT0130	FTL_1644	1683947	1682448	500	glpK	glycerol kinase	other metabolism - degradation, utilization, assimilation	unknown	
FTN_1586	FTT0129	FTL_1645	1685317	1684007	437	-	sugar transporter, MFS superfamily	transport - carbohydrates (sugars, polysaccharides)	cytoplasmic membrane	
FTN_1587	FTT0128	FTL_1646	1685998	1685390	203	-	protein of unknown function	unknown function - novel	unknown	
FTN_1588	FTT0127c	FTL_1647	1686132	1687331	400	-	major facilitator superfamily (MFS) transport protein	transport	cytoplasmic membrane	
FTN_1589	FTT0126	(FTL_1648)	1688312	1687341	324	oppF	peptide/opine/nickel uptake transporter (PepT) family protein	transport	unknown	
FTN_1590	FTT0125	LVSnoseq	1689281	1688316	322	oppD	peptide/opine/nickel uptake transporter (PepT) family protein	transport	unknown	
FTN_1591	(FTT0124)	(1580108-1580965)	1690145	1689288	286	oppC	peptide/opine/nickel uptake transporter (PepT) family protein	transport	unknown	yes
FTN_1592	(FTT0123)	(1580968-1581835)	1691083	1690148	312	oppB	peptide/opine/nickel uptake transporter (PepT) family protein	transport	unknown	yes
FTN_1593	(FTT0122)	(FTL_1653-FTL_1654)	1692784	1691111	558	oppA	ABC-type oligopeptide transport system, periplasmic component	transport	unknown	
FTN_1594	FTT0121	FTL_1656	1695096	1692877	740	uvrD	DNA helicase II	DNA replication, recombination, modification and repair - restriction/modification	cytoplasm	
FTN_1595	FTT0120	FTL_1657	1696182	1695190	331	ftsY	cell division protein, signal recognition particle GTPase	cell cycle	unknown	
FTN_1596	FTT0119	FTL_1658	1697531	1696218	438	fimV	Type IV pili, pilus assembly protein	motility, attachment and secretion structure	unknown	
FTN_1597	FTT0118	FTL_1659	1699183	1697609	525	prfC	peptide chain release factor 3	translation, ribosomal structure and biogenesis	unknown	
FTN_1598	FTT0117	FTL_1660	1699874	1699248	209	tmk	thymidylate kinase	nucleotides and nucleosides metabolism	unknown	
FTN_1599	FTT0116	FTL_1661	1701089	1699890	400	nupC	nucleoside permease NUP family protein	transport	unknown	
FTN_1600	FTT0115	FTL_1662	1702307	1701111	399	nupC1	nucleoside permease NUP family protein	transport	unknown	
FTN_1601	FTT0114	FTL_1663	1703058	1702300	253	deoC	deoxyribose-phosphate aldolase	other metabolism - degradation, utilization, assimilation	unknown	
FTN_1602	FTT0113	FTL_1664	1704265	1703024	414	deoB	phosphopentomutase	other metabolism - degradation, utilization, assimilation	unknown	
FTN_1603	FTT0112	FTL_1665	1705046	1704273	258	-	regulatory factor, Bvg accessory factor family	signal transduction and regulation	unknown	
FTN_1604	FTT0111	FTL_1666	1707759	1705069	897	polA	DNA polymerase I	DNA replication, recombination, modification and repair - restriction/modification	cytoplasm	
FTN_1605	FTT0110	FTL_1667	1708823	1707858	322	lpxK	tetraacyldisaccharide 4'-kinase	fatty acids and lipids metabolism	unknown	
FTN_1606	FTT0109	FTL_1668	1710658	1708832	609	msbA	lipid exporter (LipidE) family protein	transport	unknown	
FTN_1607	FTT0108c	FTL_1669	1710763	1711869	369	cca	tRNA nucleotidyl transferase	translation, ribosomal structure and biogenesis	unknown	
FTN_1608	FTT0107c	FTL_1670	1711912	1712400	163	dsbB	disulfide bond formation protein	putative enzymes	unknown	
FTN_1609	FTT0106c	FTL_1671	1712416	1713786	457	-	membrane fusion protein	transport	periplasm	
FTN_1610	FTT0105c	FTL_1672	1713789	1716899	1037	-	RND efflux transporter, AcrB/AcrD/AcrF family	transport	cytoplasmic membrane	
FTN_1611	FTT0104c	FTL_1673	1717771	1719048	426	-	major facilitator superfamily (MFS) transport protein	transport	cytoplasmic membrane	
FTN_1612	FTT0103c	FTL_1674	1719296	1719874	193	-	hypothetical protein	hypothetical - novel	unknown	
FTN_1613	FTT0101	FTL_1678	1721120	1720110	337	-	peptidase, U61 family	post-translational modification, protein turnover, chaperones - protein degradation	cytoplasmic membrane	
(FTN_1614)	FTT0097	(FTL_1758)	1723925	1723670						
FTN_1615	(FTT0096)	FTL_1759	1725338	1724061	426	-	conserved protein of unknown function	unknown function - conserved	unknown	
FTN_1616	FTT0095	(FTL_1760-FTL_1761)	1726298	1725345	318	-	protein of unknown function	unknown function - novel	unknown	
FTN_1617	FTT0094c	FTL_1762	1726414	1727838	475	-	two-component regulator, sensor histidine kinase	signal transduction and regulation	unknown	
FTN_1618	FTT0093	FTL_1763	1728719	1727841	293	-	conserved protein of unknown function	unknown function - conserved	cytoplasmic membrane	

FTN 1619	(FTT0092c)	(1698739-1699735)	1728955	1730328	458	appC	cytochrome bd-II terminal oxidase subunit I	energy metabolism	unknown	yes
FTN 1620	FTT0091c	FTL 1765	1730335	1731294	320	appB	cytochrome bd-II terminal oxidase subunit II	energy metabolism	unknown	
1731308-1731751	FTT0090c	FTL 1766	1731308	1731751						
FTN 1621	(FTT0089c)	(1701158-1702141)	1731751	1732734	328	-	predicted NAD/FAD-dependent oxidoreductase	putative enzymes	unknown	yes
FTN 1622	FTT0088	(FTL_1770-FTL 1771)	1733845	1732820	342	pilT	Type IV pili nucleotide-binding protein	motility, attachment and secretion structure	unknown	
FTN 1623	FTT0087	FTL 1772	1736753	1733943	937	acnA	aconitate hydratase	energy metabolism	unknown	
FTN 1624	FTT0086	FTL 1773	1737736	1736861	292	-	conserved protein of unknown function	unknown function - conserved	unknown	
FTN 1625	FTT0085c	FTL 1774	1737806	1738180	125	-	GtrA-like protein	putative enzymes	unknown	
FTN 1626	FTT0084c	FTL 1775	1738188	1739327	380	hemN	coproporphyrinogen III oxidase, anaerobic	cofactors, prosthetic groups, electron carriers metabolism	unknown	
FTN 1627	FTT0083	FTL 1776	1739779	1739330	150	-	protein of unknown function	unknown function - novel	unknown	
FTN 1628	(FTT0082)	(1709202-1710153)	1740791	1739841	317	-	transcriptional regulator, LysR family	signal transduction and regulation	unknown	yes
FTN 1630	FTT0081	FTL 1779	1741314	1740964	117	secG	preprotein translocase, subunit G, membrane protein	motility, attachment and secretion structure	unknown	
FTN 1631	FTT0080	FTL 1780	1742063	1741305	253	tpiA	triosephosphate isomerase	energy metabolism	unknown	
FTN 1632	FTT0079	FTL 1781	1743397	1742069	443	mrsA	phosphoglucosamine mutase	other metabolism - degradation, utilization, assimilation	unknown	
FTN 1633	FTT0078	FTL 1782	1743935	1743411	175	apt	adenine phosphoribosyltransferase	nucleotides and nucleosides metabolism	unknown	
FTN 1634	FTT0077	FTL 1783	1745444	1743978	489	sucB	2-oxoglutarate dehydrogenase complex, E2 component, dihydrolipoyltranssuccinase	energy metabolism	unknown	
FTN 1635	FTT0076	FTL 1784	1748283	1745473	937	sucA	2-oxoglutarate dehydrogenase complex, E1 component, 2-oxoglutarate decarboxylase	energy metabolism	unknown	
FTN 1636	FTT0075	FTL 1785	1749014	1748316	233	sdhB	succinate dehydrogenase iron-sulfur protein	energy metabolism	unknown	
FTN 1637	FTT0074	FTL 1786	1750825	1749035	597	sdhA	succinate dehydrogenase flavoprotein	energy metabolism	unknown	
FTN 1638	FTT0073	FTL 1787	1751206	1750841	122	sdhD	succinate dehydrogenase hydrophobic membrane anchor protein	energy metabolism	unknown	
FTN 1639	FTT0072	FTL 1788	1751655	1751185	157	sdhC	succinate dehydrogenase, cytochrome b556	energy metabolism	unknown	
FTN 1640	FTT0071c	FTL 1789	1751913	1753169	419	gltA	citrate synthase	energy metabolism	unknown	
FTN 1641	FTT0070c	FTL 1790	1753261	1754523	421	ampG	peptide-acetyl-coenzyme A transporter (PAT) family protein	transport	cytoplasmic membrane	
FTN 1642	FTT0068	FTL 1791	1755309	1754734	192	sodB	iron/manganese superoxide dismutase family protein	other metabolism - degradation, utilization, assimilation	unknown	
FTN 1643	FTT0067c	FTL 1792	1755445	1755771	109	-	glutaredoxin-related protein	putative enzymes	unknown	
FTN 1644	FTT0066	FTL 1793	1758634	1755794	947	-	protein of unknown function	unknown function - novel	unknown	
FTN 1645	FTT0065	FTL 1794	1760144	1759710	145	atpC	ATP synthase, F1 sector, subunit epsilon	energy metabolism	unknown	
FTN 1646	FTT0064	FTL 1795	1761533	1760160	458	atpD	ATP synthase, F1 sector, subunit beta	energy metabolism	unknown	
FTN 1647	FTT0063	FTL 1796	1762439	1761546	298	atpG	ATP synthase, F1 sector, subunit gamma	energy metabolism	unknown	
FTN 1648	FTT0062	FTL 1797	1763995	1762457	513	atpA	ATP synthase, F1 sector, subunit alpha	energy metabolism	unknown	
FTN 1649	FTT0061	FTL 1798	1764538	1764017	174	atpH	ATP synthase, F1 sector, subunit delta	energy metabolism	unknown	
FTN 1650	FTT0060	FTL 1799	1765028	1764561	156	atpF	ATP synthase, F0 sector, subunit b	energy metabolism	cytoplasm	
FTN 1651	FTT0059	FTL 1800	1765379	1765077	101	atpE	ATP synthase, F0 sector, subunit c	energy metabolism	unknown	
FTN 1652	FTT0058	FTL 1801	1766221	1765433	263	atpB	ATP synthase, F0 sector, subunit a	energy metabolism	unknown	
FTN 1653	FTT0057	FTL 1802	1766705	1766277	143	-	hypothetical membrane protein	hypothetical - novel	cytoplasmic membrane	
FTN 1654	FTT0056c	FTL 1803	1766782	1768083	434	-	major facilitator superfamily (MFS) transport protein	transport	cytoplasmic membrane	
FTN 1655	FTT0055	FTL 1804	1769017	1768091	309	rluC	ribosomal large subunit pseudouridine synthase C	translation, ribosomal structure and biogenesis	unknown	
FTN 1656	FTT0054	FTL 1805	1770077	1769013	355	-	conserved hypothetical protein	hypothetical - conserved	unknown	
FTN 1657	FTT0053	FTL 1806	1771378	1770083	432	-	major facilitator superfamily (MFS) transport protein	transport	cytoplasmic membrane	
FTN 1658	FTT0052	FTL 1807	1772650	1771388	421	hisS	histidyl-tRNA synthetase	other metabolism - biosynthesis	unknown	
FTN 1659	FTT0051	FTL 1808	1773089	1772661	143	rbfA	ribosome-binding factor A	translation, ribosomal structure and biogenesis	unknown	
FTN 1660	FTT0050	FTL 1809	1775632	1773095	846	infB	translation initiation factor IF-2	translation, ribosomal structure and biogenesis	unknown	
FTN 1661	FTT0049	FTL 1810	1777140	1775674	489	nusA	transcription elongation factor	transcription	unknown	
FTN 1662	FTT0048	FTL 1811	1777610	1777161	150	-	conserved protein of unknown function	unknown function - conserved	unknown	
FTN 1664	FTT0047	FTL 1812	1778870	1777839	344	hemE	uroporphyrinogen decarboxylase	cofactors, prosthetic groups, electron carriers metabolism	unknown	
FTN 1665	(FTT0046)	(1747428-1748848)	1780432	1778927	502	-	magnesium chelatase	cofactors, prosthetic groups, electron carriers metabolism	unknown	yes
FTN 1666	FTT0045	1749887-1750078	1780628	1780437	64	-	conserverd protein of unknown function	unknown function - conserved	unknown	
FTN 1667	FTT0044	FTL 1817	1782155	1780707	483	nuoN	NADH dehydrogenase I, N subunit	energy metabolism	cytoplasmic membrane	
FTN 1668	FTT0043	FTL 1818	1783760	1782174	529	nuoM	NADH dehydrogenase I, M subunit	energy metabolism	cytoplasmic membrane	
FTN 1669	FTT0042	FTL 1819	1785795	1783789	669	nuoL	NADH dehydrogenase I, L subunit	energy metabolism	cytoplasmic membrane	
FTN 1670	FTT0041	FTL 1820	1786122	1785808	105	nuoK	NADH dehydrogenase I, K subunit	energy metabolism	unknown	
FTN 1671	FTT0040	FTL 1821	1786720	1786118	201	nuoJ	NADH dehydrogenase I, J subunit	energy metabolism	unknown	
FTN 1672	FTT0039	FTL 1822	1787213	1786728	162	nuoI	NADH dehydrogenase I, I subunit	energy metabolism	unknown	
FTN 1673	FTT0038	FTL 1823	1788237	1787230	336	nuoH	NADH dehydrogenase I, H subunit	energy metabolism	unknown	
FTN 1674	FTT0037	FTL 1824	1790605	1788242	788	nuoG	NADH dehydrogenase I, G subunit	energy metabolism	unknown	
FTN 1675	FTT0036	FTL 1825	1791897	1790626	424	nuoF	NADH dehydrogenase I, F subunit	energy metabolism	unknown	
FTN 1676	FTT0035	FTL 1826	1792392	1791907	162	nuoE	NADH dehydrogenase I, E subunit	energy metabolism	unknown	
FTN 1677	FTT0034	FTL 1827	1793655	1792405	417	nuoD	NADH dehydrogenase I, D subunit	energy metabolism	unknown	
FTN 1678	FTT0033	FTL 1828	1794321	1793680	214	nuoC	NADH dehydrogenase I, C subunit	energy metabolism	unknown	
FTN 1679	FTT0032	FTL 1829	1794800	1794327	158	nuoB	NADH dehydrogenase I, B subunit	energy metabolism	unknown	
FTN 1680	FTT0031	FTL 1830	1795189	1794794	132	nuoA	NADH dehydrogenase I, A subunit	energy metabolism	unknown	
FTN 1681	FTT0030c	FTL 1831	1795398	1795817	140	fur	ferric uptake regulation protein	signal transduction and regulation	unknown	
FTN 1682	FTT0029c	FTL 1832	1796074	1797990	639	frgA	siderophore biosynthesis protein	other metabolism - biosynthesis	unknown	
FTN 1683	FTT0028c	FTL 1833	1798000	1799259	420	-	drug:H+ antiporter-1 (DHA1) family protein	transport - drugs / antibacterial compounds	cytoplasmic membrane	
FTN 1684	FTT0027c	FTL 1834	1799255	1800505	417	-	pyridoxal-dependent decarboxylase	putative enzymes	unknown	
FTN 1685	FTT0026c	FTL 1835	1800523	1801710	396	-	drug:H+ antiporter-1 (DHA1) family protein	transport - drugs / antibacterial compounds	cytoplasmic membrane	
FTN 1686	FTT0025c	FTL 1836	1801799	1803325	509	-	hypothetical membrane protein	hypothetical - novel	cytoplasmic membrane	
FTN 1687	(FTT0023c-FTT0024c)	(FTL_1837-FTL 1839)	1803376	1804866	497	-	GDSL-like lipolytic enzyme	fatty acids and lipids metabolism	unknown	yes
FTN 1688	FTT0022	FTL 1840	1805292	1804888	135	-	protein of unknown function	unknown function - novel	cytoplasm	
FTN 1689	FTT0021	FTL 1841	1806728	1805310	473	gatB	Glu-tRNAGln amidotransferase B subunit	translation, ribosomal structure and biogenesis	unknown	
FTN 1690	FTT0020	FTL 1842	1808176	1806734	481	gatA	Glu-tRNAGln amidotransferase A subunit	translation, ribosomal structure and biogenesis	unknown	
FTN 1691	FTT0019	FTL 1843	1808466	1808188	93	gatC	Glu-tRNAGln amidotransferase C subunit	translation, ribosomal structure and biogenesis	unknown	
FTN 1692	FTT0018	(FTL_1844-FTL 1845)	1809619	1808552	356	-	membrane fusion protein	motility, attachment and secretion structure	periplasm	
FTN 1693	FTT0017	(FTL_1846-FTL 1847)	1811171	1809633	513	-	ATP-binding cassette (ABC) superfamily protein	transport	cytoplasmic membrane	
FTN 1694	FTT0015	FTL 1850	1812625	1811330	432	purB	adenylosuccinate lyase	nucleotides and nucleosides metabolism	unknown	
FTN 1695	(FTT0014c)	(FTL 1852)	1812953	1813585	211	-	protein of unknown function	unknown function - novel	unknown	yes
FTN 1696	FTT0013c	FTL 1853	1813595	1814374	260	-	protein of unknown function	unknown function - novel	unknown	
FTN 1697	(FTT0012)	FTL 1854	1815263	1814406	286	-	galactose mutarotase	carbohydrate metabolism - biosynthesis	unknown	
FTN 1698	Schunoseq	LVSnoseq	1816619	1815852	256	-	Dam-replacing family protein	DNA replication, recombination, modification and repair - restriction/modification	unknown	
FTN 1699	FTT1720c	FTL 1860	1820534	1816650	1295	purL	phosphoribosylformylglycinamide synthase	nucleotides and nucleosides metabolism	unknown	
FTN 1700	FTT1721c	FTL 1861	1822024	1820537	496	purF	amidophosphoribosyltransferase	nucleotides and nucleosides metabolism	unknown	
FTN 1701	FTT1722c	(FTL 1863)	1823464	1822121	448	-	glutamate decarboxylase	amino acid metabolism - degradation, utilization, assimilation	unknown	
FTN 1702	FTT1723c	FTL 1864	1824116	1823478	213	-	phosphopantetheinyl transferase	cofactors, prosthetic groups, electron carriers metabolism	unknown	
FTN 1703	FTT1724c	FTL 1865	1825654	1824128	509	tolC	outer membrane efflux protein, tolC precursor	transport	unknown	
FTN 1704	FTT1725c	FTL 1866	1826283	1825666	206	pcm	protein-L-isoaspartate O-methyltransferase	post-translational modification, protein turnover, chaperones - protein modification	unknown	
FTN 1705	FTT1726	FTL 1867	1826603	1827931	443	-	peptidase, U32 family	post-translational modification, protein turnover, chaperones - protein degradation	unknown	
FTN 1706	FTT1727c	FTL 1868	1829157	1827979	393	-	drug:H+ antiporter-1 (DHA1) family protein	transport - drugs / antibacterial compounds	cytoplasmic membrane	
FTN 1707	FTT1728	FTL 1869	1829332	1830756	475	nhaD	Na+:H+ antiporter	transport	cytoplasmic membrane	
FTN 1708	(FTT1729c)	(1802334-1803545)	1832410	1830752	553	-	ATP-binding cassette (ABC) superfamily protein	transport	unknown	yes
FTN 1709	FTT1730c	FTL 1873	1834285	1832414	624	-	amino acid-polyamine-organocation (APC) superfamily protein	transport - amino-acid	cytoplasmic membrane	
FTN 1710	FTT1731c	FTL 1874	1835296	1834295	334	-	GTP-binding protein, GTP1/Obg family	putative enzymes	unknown	

FTN 1711	FTT1732c	FTL 1875	1836565	1835372	398	tyrP	tyrosine permease	transport - amino-acid	cytoplasmic membrane	
FTN 1712	(FTT1733)	LVSnoseq	1836693	1837145	151	-	hypothetical protein	hypothetical - novel	unknown	
FTN 1713	(FTT1734c)	(FTL_1876-FTL 1876)	1837808	1837164	215	-	protein of unknown function	unknown function - novel	unknown	
FTN 1714	(FTT1735c)	(1809367-1809735)	1838559	1837876	228	kdpE	two-component response regulator	signal transduction and regulation	cytoplasm	yes
FTN 1715	FTT1736c	(FTL_1878-FTL 1879)	1841282	1838604	893	kdpD	two component regulator, sensor histidine kinase kdpD	signal transduction and regulation	cytoplasmic membrane	
FTN 1716	FTT1737c	FTL 1880	1841940	1841389	184	kdpC	potassium-transporting ATPase C chain	transport	unknown	
FTN 1717	FTT1738c	(FTL_1881-FTL 1882)	1843996	1841960	679	kdpB	potassium-transporting ATPase B chain	transport	unknown	
FTN 1718	(FTT1739c)	FTL 1883	1845727	1844009	573	kdpA	K(+)-ATPase uptake protein	transport	unknown	
FTN 1719	(FTT1698c-FTT1741c)	(FTL_0127-FTL 1884)	1847178	1846033	382	-	D-isomer specific 2-hydroxyacid dehydrogenase	energy metabolism	unknown	yes
FTN 1725	Schunoseq	LVSnoseq	1853917	1852730	396	aspC	aspartate aminotransferase	amino acid metabolism - degradation, utilization, assimilation	unknown	
FTN 1726	Schunoseq	LVSnoseq	1855098	1853938	387	-	pyridoxal-dependent decarboxylase	amino acid metabolism - biosynthesis	cytoplasm	
FTN 1727	Schunoseq	LVSnoseq	1855901	1855155	249	dapD	tetrahydrodipicolinate succinylase subunit	amino acid metabolism - biosynthesis	unknown	
FTN 1728	Schunoseq	LVSnoseq	1856812	1855928	295	dapA	dihydrodipicolinate synthase	amino acid metabolism - biosynthesis	unknown	
FTN 1729	Schunoseq	LVSnoseq	1857474	1856806	223	dapB	dihydrodipicolinate reductase	amino acid metabolism - biosynthesis	unknown	
FTN 1730	Schunoseq	LVSnoseq	1858841	1857489	451	lysC	aspartate kinase III	amino acid metabolism - biosynthesis	unknown	
FTN 1731	Schunoseq	LVSnoseq	1860191	1859256	312	pip	proline iminopeptidase	post-translational modification, protein turnover, chaperones - protein degradation	unknown	
FTN 1732	Schunoseq	LVSnoseq	1861013	1860270	248	-	Mg-dependent DNase	DNA replication, recombination, modification and repair - degradation	unknown	
FTN 1733	(FTT1779)	FTL 0184	1861637	1861017	207	-	nicotinamide ribonucleoside (NR) uptake permease (PnuC) family protein	transport	unknown	
FTN 1734	FTT1778c	FTL 0105	1861861	1862250	130	-	protein of unknown function	unknown function - novel	unknown	
FTN 1735	FTT1777c	FTL 0104	1862274	1862681	136	-	protein of unknown function	unknown function - novel	unknown	
FTN 1736	FTT1776c	(FTL_0103-FTL 0103)	1862687	1863082	132	-	hypothetical protein	hypothetical - novel	unknown	
FTN 1737	FTT1775c	FHF0994.1(FTL 0101)	1863156	1864571	472	eriC	Cl-:H+ antiporter	transport	cytoplasmic membrane	
FTN 1738	(FTT1774c)	FTL 0100	1864510	1866012	501	-	metallocarboxypeptidase	post-translational modification, protein turnover, chaperones - protein degradation	unknown	
FTN 1739	FTT1773c	FTL 0099	1866821	1868008	396	trpB	tryptophan synthase beta chain	amino acid metabolism - biosynthesis	unknown	
FTN 1740	FTT1772c	FTL 0098	1868014	1868817	268	trpA	tryptophan synthase alpha chain	amino acid metabolism - biosynthesis	unknown	
FTN 1741	FTT1771	FTL 0097	1869228	1868878	117	-	protein of unknown function	unknown function - novel	cytoplasm	
FTN 1742	(FTT1770)	(92138-92772)	1870028	1869321	236	pnuC	nicotinamide ribonucleoside (NR) uptake permease (PnuC) family protein	transport	unknown	
FTN 1743	FTT1769c	FTL 0094	1870244	1872820	859	clpB	chaperone clpB	post-translational modification, protein turnover, chaperones - chaperones	unknown	
FTN 1744	(FTT1768c)	FTL 0093	1873074	1875263	730	chiB	chitinase	carbohydrate metabolism - degradation, utilization, assimilation	unknown	
FTN 1745	FTT1767c	FTL 0092	1875320	1876477	386	purT	phosphoribosylglycinamide formyltransferase 2	nucleotides and nucleosides metabolism	unknown	
(FTN_1746)	FTT1766	(FTL 0091)	1876485	1877140						
FTN 1747	FTT1765	FTL 0090	1877859	1877221	213	-	hypothetical membrane protein	hypothetical - novel	cytoplasmic membrane	
FTN 1748	FTT1764c	FTL 0089	1878089	1878409	107	-	4Fe-4S ferredoxin	energy metabolism	unknown	
FTN 1749	FTT1763c	FTL 0088	1878575	1879324	250	-	acyltransferase	fatty acids and lipids metabolism	unknown	
FTN 1750	FTT1762c	FTL 0087	1879341	1880078	246	-	acyltransferase	fatty acids and lipids metabolism	unknown	
FTN 1751	FTT1761	FTL 0086	1880806	1880090	239	-	conserved hypothetical protein	hypothetical - conserved	unknown	
FTN 1752	FTT1760	FTL 0085	1882022	1880874	383	nhaA	Na+:H+ antiporter	transport	cytoplasmic membrane	
FTN 1753	(FTT1759c)	(79155-80048)	1882116	1883009	298	-	Rieske (2Fe-2S) domain protein	putative enzymes	unknown	
FTN 1754	FTT1758c	FTL 0082	1883079	1883585	169	-	cytochrome b561 family protein	cofactors, prosthetic groups, electron carriers metabolism	cytoplasmic membrane	
FTN 1755	(FTT1757c)	(77851-78498)	1883615	1884871	419	-	metabolite:H+ symporter (MHS) family protein	transport	cytoplasmic membrane	
FTN 1756	Schunoseq	LVSnoseq	1885175	1885651	159	bcp	bacterioferritin comigratory protein	post-translational modification, protein turnover, chaperones - protein modification	unknown	
FTN 1757	Schunoseq	LVSnoseq	1886715	1885735	327	-	D-isomer specific 2-hydroxyacid dehydrogenase	energy metabolism	unknown	
FTN 1758	Schunoseq	LVSnoseq	1887821	1888438	206	-	hypothetical protein	hypothetical - novel	unknown	
FTN 1759	Schunoseq	LVSnoseq	1888901	1888512	130	-	protein of unknown function	unknown function - novel	unknown	
FTN 1760	Schunoseq	LVSnoseq	1889010	1889996	329	-	zinc-binding alcohol dehydrogenase	carbohydrate metabolism	unknown	
(FTN_1761)	FTT1781c	LVSnoseq	1889997	1890746						
FTN 1762	FTT1782c	FTL 1947	1892423	1890747	559	-	(putative) drug resistance ATPase-1 (Drug RA1) family protein	transport - drugs / antibacterial compounds	unknown	
FTN 1763	FTT1783	FTL 1948	1892635	1893852	406	-	metabolite:H+ symporter (MHS) family protein	transport	cytoplasmic membrane	
FTN 1764	FTT1785c	FTL 1949	1894084	1893875	70	-	protein of unknown function	unknown function - novel	cytoplasm	
FTN 1765	(FTT1786)	FTL 1950	1894130	1895098	323	-	conserved hypothetical protein	hypothetical - conserved	unknown	
FTN 1766	Schunoseq	LVSnoseq	1896115	1895114	334	-	drug/metabolite transporter (DMT) superfamily protein	transport - drugs / antibacterial compounds	unknown	
FTN 1767	Schunoseq	LVSnoseq	1897052	1896135	306	rbsK	ribokinase, pfkB family	carbohydrate metabolism - degradation, utilization, assimilation	unknown	
FTN 1768	FTT1793c	FTL 1956	1899746	1897173	858	pepN	aminopeptidase N	post-translational modification, protein turnover, chaperones - protein modification	unknown	
FTN 1769	FTT1794	FTL 1957	1899948	1900373	142	-	heat shock protein, HSP20 family	post-translational modification, protein turnover, chaperones - chaperones	unknown	
FTN 1770	FTT1795c	FTL 1958	1901734	1900376	453	trpC	indole-3-glycerol phosphate synthase (trpC); phosphoribosylanthranilate isomerase (trpF)	amino acid metabolism - biosynthesis	unknown	
FTN 1771	FTT1796c	(FTL 1959)	1902239	1901829	137	-	conserved hypothetical protein	hypothetical - conserved	unknown	
FTN 1772	FTT1797c	FTL 1960	1902933	1902247	229	-	peptide methionine sulfoxide reductase	post-translational modification, protein turnover, chaperones - protein modification	unknown	
FTN 1773	FTT1798c	FTL 1961	1903385	1903020	122	-	hypothetical protein	hypothetical - novel	unknown	
FTN 1774	(FTT1799c)	FTL 1962	1903719	1903381	113	-	protein of unknown function	unknown function - novel	unknown	
FTN 1775	FTT1800c	FTL 1963	1904228	1903791	146	-	protein of unknown function	unknown function - novel	unknown	
FTN 1776	FTT1801b	1890384-1891406	1905303	1904293	337	trpD	anthranilate phosphoribosyltransferase	amino acid metabolism - biosynthesis	unknown	
FTN 1777	FTT1801a	1891394-1891969	1905878	1905303	192	trpG	anthranilate synthase component II	amino acid metabolism - biosynthesis	unknown	
FTN 1778	FTT1802c	FTL 1966	1907426	1905885	514	trpE	anthranilate synthase component I	amino acid metabolism - biosynthesis	unknown	
FTN 1779	FTT1803c	FTL 1967	1907722	1907438	95	trpR	trp operon repressor	signal transduction and regulation	unknown	
FTN 1782	FTT1804c	FTL 1968	1909693	1908200	498	rng	ribonuclease G	translation, ribosomal structure and biogenesis	unknown	

[1] The locus tags of pseudogenes have been put between parenthesis. When several locus tags are present, it indicates that they cover the same pseudogenes. For genes where no locus tag was available, the genome coordinate[illegible]
