# Supplementary material for: Comparison of Francisella tularensis genomes reveals evolutionary events associated with the emergence of human pathogenic strains
Source: Genome Biol. 2007 Jun 5;8(6):R102. doi: 10.1186/gb-2007-8-6-r102 (PMC2394750; doi:10.1186/gb-2007-8-6-r102)
Supplement: Additional data file 2 — Eighty candidate virulence genes of Francisella tularensis subspecies novicida U112 are also present in holarctica and tularensis genomes. [file gb-2007-8-6-r102-S2.pdf]

Supplemental Table 2: 80 candidate virulence genes of *F.t. novicida U112* are also present in *holarctica* and *tularensis* genomes.

Protein feature suggesting role in virulence	Accession number in F.t.n. U112	Accession number in F.t.t. Schu S4	Protein size (aa)	Gene name	Function description	Presence of a signal peptide predicted by SignalP	First COG hit with a rps-blast search (e-value resulting from rps-blast)	PFAM domains detected in the protein sequence and the e-value resulting from the search with the software HMMER	Prosite domains detected in the protein sequence
DOMAINS ASSOCIATED WITH VIRULENCE	FTN_0023	FTT1661	226	*tmpT*	thiopurine S-methyltransferase	no signal peptide detected		TPMT_ Thiopurine S-methyltransferase (TPMT)_ PF05724.1 2.1e-37	
DOMAINS ASSOCIATED WITH VIRULENCE	FTN_0031	FTT1652c	289	-	transcriptional regulator, LysR family	no signal peptide detected	COG0583_LysR_Transcriptional regulator_Transcription_8e-33	HTH_1_ Bacterial regulatory helix-turn-helix protein, lysR family_ PF00126.12_1e-10;LysR_substrate_ LysR substrate binding domain PF03466.6 7e-14	PS50931_HTH_LYSR_LysR-type HTH domain profile
DOMAINS ASSOCIATED WITH VIRULENCE	FTN_0070	FTT0230c	135	*pilE*	Type IV pili, pilus assembly protein	no signal peptide detected	COG4968_PilE_Tfp pilus assembly protein PilE_Cell motility and secretion / Intracellular trafficking and secretion 1e-16	N_methyl_ Prokaryotic N-terminal methylation motif_ PF07963.1_7e-09	PS00409_PROKAR_NTER_METHYL_Prokaryotic N-terminal methylation site
EUKARYOTIC DOMAINS	FTN_0083	FTT0243	252	-	conserved protein of unknown function	signal peptide detected	COG5184_ATS1_Alpha-tubulin suppressor and related RCC1 domain-containing proteins_Cell division and chromosome partitioning / Cytoskeleton_5e-12		PS50012_RCC1_3_Regulator of chromosome condensation (RCC1) repeat profile;PS50012_RCC1_3_Regulator of chromosome condensation (RCC1) repeat profile
DOMAINS ASSOCIATED WITH VIRULENCE	FTN_0090	FTT0221	514	-	acid phosphatase	signal peptide detected	COG3511_PlcC_Phospholipase C_Cell envelope biogenesis, outer membrane 1e-26	Phosphoesterase_ Phosphoesterase family_ PF04185.2 3.6e-182	
EUKARYOTIC DOMAINS	FTN_0132	FTT0256c	322	-	protein of unknown function	no signal peptide detected			
DOMAINS ASSOCIATED WITH VIRULENCE	FTN_0134	FTT0258	222	-	carboxylesterase/phospholipase family protein	no signal peptide detected	COG0400_COG0400_Predicted esterase_General function prediction only_4e-33	Abhydrolase_2_ Phospholipase/Carboxylesterase_ PF02230.6 5e-72	
EUKARYOTIC DOMAINS	FTN_0171	FTT0195c	513	*glsA*	glutaminase	no signal peptide detected	COG2066_GlsA_Glutaminase_Amino acid transport and metabolism_5e-81	Glutaminase_ Glutaminase_ PF04960.4_7.6e-130;Ank_ Ankyrin repeat_ PF00023.15_1.7e-11	PS50088_ANK_REPEAT_Ankyrin repeat profile;PS50297_ANK_REP_REGION_Ankyrin repeat region circular profile
DOMAINS ASSOCIATED WITH VIRULENCE	FTN_0200	FTT0286c	347	-	UDP-3-O-[3-fatty acid] glucosamine N-acyltransferase	no signal peptide detected	COG1044_LpxD_UDP-3-O-[3-hydroxymyristoyl]__7e-78	LpxD_ UDP-3-O-[3-hydroxymyristoyl] glucosamine N-acyltransferase, LpxD_ PF04613.2_6e-05;Hexapep_ Bacterial transferase hexapeptide (three repeats)_ PF00132.10_0.0022;Hexapep_ Bacterial transferase hexapeptide (three repeats)_ PF00132.10_0.0047;Hexapep_ Bacterial transferase hexapeptide (three repeats)_ PF00132.10_0.014;Hexapep_ Bacterial transferase hexapeptide (three repeats)_ PF00132.10 0.091	PS00101_HEXAPEP_TRANSFERASES_Hexapeptide-repeat containing-transferases signature
EUKARYOTIC DOMAINS	FTN_0275	FTT0369c	352	-	conserved protein of unknown function	signal peptide detected	COG0790_COG0790_FOG: TPR repeat, SEL1 subfamily_General function prediction only_6e-04		
DOMAINS ASSOCIATED WITH VIRULENCE	FTN_0276	FTT0368c	513	*mviN*	multidrug/oligosaccharidyl-lipid/polysaccharide (MOP) transporter	signal peptide detected	COG0728_MviN_Uncharacterized membrane protein, putative virulence factor_General function prediction only 1e-51	MVIN_ MviN-like protein_ PF03023.4_4.9e-50;Na_H_Exchanger_ Sodium/hydrogen exchanger family PF00999.9 0.041	
DOMAINS ASSOCIATED WITH VIRULENCE	FTN_0303	FTT1627c	308	-	pilus assembly protein	no signal peptide detected	COG4966_PilW_Tfp pilus assembly protein PilW_Cell motility and secretion / Intracellular trafficking and secretion 2e-07	N_methyl_ Prokaryotic N-terminal methylation motif_ PF07963.1_9.3e-07	PS00409_PROKAR_NTER_METHYL_Prokaryotic N-terminal methylation site
DOMAINS ASSOCIATED WITH VIRULENCE	FTN_0322	FTT1591	366	-	VacJ like lipoprotein	signal peptide detected	COG2853_VacJ_Surface lipoprotein_Cell envelope biogenesis, outer membrane_1e-37	VacJ_ VacJ like lipoprotein_ PF04333.2_2.1e-38	PS00013_PROKAR_LIPOPROTEIN_Prokaryotic membrane lipoprotein lipid attachment site
DOMAINS ASSOCIATED WITH VIRULENCE	FTN_0347	FTT0832	154	*fkpB*	FKBP-type peptidyl-prolyl cis-trans isomerase	no signal peptide detected	COG1047_SlpA_FKBP-type peptidyl-prolyl cis-trans isomerases 2 _Posttranslational modification, protein turnover, chaperones_3e-30	FKBP_C_ FKBP-type peptidyl-prolyl cis-trans isomerase_ PF00254.14_0.00041	PS50059_FKBP_PPIASE_FKBP-type peptidyl-prolyl cis-trans isomerase domain profile
DOMAINS ASSOCIATED WITH VIRULENCE	FTN_0398	FTT0872c	178	-	hypothetical membrane protein	no signal peptide detected		Colicin_V_ Colicin V production protein_ PF02674.5 0.0016	
EUKARYOTIC DOMAINS	FTN_0403	FTT0877c	217	-	hypothetical membrane protein	no signal peptide detected		PQ-loop_ PQ loop repeat_ PF04193.4_0.00056;PQ-loop_ PQ loop repeat_ PF04193.4 3e-07	
DOMAINS ASSOCIATED WITH VIRULENCE	FTN_0416	FTT0891	239	*lpxE*	lipid A 1-phosphatase	no signal peptide detected	COG0671_PgpB_Membrane-associated phospholipid phosphatase _Lipid metabolism_4e-05	PAP2_ PAP2 superfamily_ PF01569.9_5.2e-10	
DOMAINS ASSOCIATED WITH VIRULENCE	FTN_0425	FTT0899c	668	*prlC*	oligopeptidase A	no signal peptide detected	COG0339_Dcp_Zn-dependent oligopeptidases_Amino acid transport and metabolism 0.0	Peptidase_M3_ Peptidase family M3_ PF01432.8_4.2e-145	PS00142_ZINC_PROTEASE_"Neutral zinc metallopeptidases, zinc-binding region signature"
DOMAINS ASSOCIATED WITH VIRULENCE	FTN_0426	FTT0900	123	-	conserved protein of unknown function	no signal peptide detected	COG3737_COG3737_Uncharacterized conserved protein Function unknown 1e-12	DUF598_ Protein of unknown function, DUF598_ PF04635.3 5.1e-54	
DOMAINS ASSOCIATED WITH VIRULENCE	FTN_0427	FTT0901	149	-	lipoprotein of unknown function	signal peptide detected			PS00013_PROKAR_LIPOPROTEIN_Prokaryotic membrane lipoprotein lipid attachment site
EUKARYOTIC DOMAINS	FTN_0429	FTT0903	167	-	conserved protein of unknown function	signal peptide detected	COG4372_COG4372_Uncharacterized protein conserved in bacteria with the myosin-like domain Function unknown 4e-04		PS00013_PROKAR_LIPOPROTEIN_Prokaryotic membrane lipoprotein lipid attachment site
DOMAINS ASSOCIATED WITH VIRULENCE	FTN_0431	FTT0905	480	-	hypothetical membrane protein	no signal peptide detected		Wzy_C_ O-Antigen Polymerase_ PF04932.4 7.9e-09	
EUKARYOTIC DOMAINS	FTN_0458	FTT1299	112	*hitA*	histidine triad (HIT) family protein	no signal peptide detected	COG0537_Hit_Diadenosine tetraphosphate (Ap4A) hydrolase and other HIT family hydrolases_Nucleotide transport and metabolism / Carbohydrate transport and metabolism / General function prediction only_8e-27	HIT_ HIT domain_ PF01230.11_1e-47	
EUKARYOTIC DOMAINS	FTN_0478	FTT1342	351	-	conserved hypothetical membrane protein	no signal peptide detected		Neur_chan_LBD_ Neurotransmitter-gated ion-channel ligand binding domain_ PF02931.10 5.3e-11	
EUKARYOTIC DOMAINS	FTN_0491	FTT0394	282	-	hypothetical protein	no signal peptide detected			
EUKARYOTIC DOMAINS	FTN_0494	FTT0398c	199	-	hypothetical membrane protein	signal peptide detected			
DOMAINS ASSOCIATED WITH VIRULENCE	FTN_0496	FTT0400	658	*slt*	soluble lytic murein transglycosylase	signal peptide detected	COG0741_MltE_Soluble lytic murein transglycosylase and related regulatory proteins (some contain LysM/invasin domains)_Cell envelope biogenesis, outer membrane_3e-23	SLT_ Transglycosylase SLT domain_ PF01464.8_2.9e-23	
DOMAINS ASSOCIATED WITH VIRULENCE	FTN_0500	FTT0403	211	-	peptide deformylase	no signal peptide detected	COG0242_Def_N-formylmethionyl-tRNA deformylase_Translation, ribosomal structure and biogenesis 6e-28	Pep_deformylase_ Polypeptide deformylase_ PF01327.9_9e-13	PS00142_ZINC_PROTEASE_"Neutral zinc metallopeptidases, zinc-binding region signature"
EUKARYOTIC DOMAINS	FTN_0561	FTT0470	275	*apaH*	diadenosine tetraphosphatase	no signal peptide detected	COG0639_ApaH_Diadenosine tetraphosphatase and related serine/threonine protein phosphatases_Signal transduction mechanisms 3e-04	Metallophos_ Calcineurin-like phosphoesterase_ PF00149.14_2.1e-15	
DOMAINS ASSOCIATED WITH VIRULENCE	FTN_0580	FTT0489c	316	*trxB*	thioredoxin reductase	no signal peptide detected	COG0492_TrxB_Thioredoxin reductase_Posttranslational modification, protein turnover, chaperones_4e-96	Pyr_redox_ Pyridine nucleotide-disulphide oxidoreductase_ PF00070.14_6.8e-81	PS00573_PYRIDINE_REDOX_2_Pyridine nucleotide-disulphide oxidoreductases class-II active site
DOMAINS ASSOCIATED WITH VIRULENCE	FTN_0581	FTT0490c	405	-	phospholipase D family protein	signal peptide detected	COG1502_Cls_ Phosphatidylserine/phosphatidylglycerophosphate /cardiolipin synthases and related enzymes_Lipid metabolism_1e-20	PLDc_ Phospholipase D Active site motif_ PF00614.9_0.015	PS50035_PLD_Phospholipase D phosphodiesterase active site profile;PS50035_PLD_Phospholipase D phosphodiesterase active site profile
DOMAINS ASSOCIATED WITH VIRULENCE	FTN_0583	FTT0492c	308	-	transcriptional regulator, LysR family	no signal peptide detected	COG0583_LysR_Transcriptional regulator_Transcription_2e-40	HTH_1_ Bacterial regulatory helix-turn-helix protein, lysR family_ PF00126.12_1.4e-14;LysR_substrate_ LysR substrate binding domain PF03466.6 2.7e-20	PS50931_HTH_LYSR_LysR-type HTH domain profile
DOMAIN ASSOCIATED WITH TOXIN SYNTHESIS	FTN_0604	FTT0694	699	-	AMP-binding protein	no signal peptide detected	COG0318_CaiC_Acyl-CoA synthetases (AMP-forming)/AMP-acid ligases II_Lipid metabolism / Secondary metabolites biosynthesis, transport, and catabolism 3e-71	AMP-binding_ AMP-binding enzyme_ PF00501.13_6.4e-64	PS00455_AMP_BINDING_Putative AMP-binding domain signature
DOMAINS ASSOCIATED WITH VIRULENCE	FTN_0627	FTT0715	870	*chiA*	chitinase, glycosyl hydrolase family 18	signal peptide detected	COG3325_ChiA_Chitinase_Carbohydrate transport and metabolism_5e-41	Glyco_hydro_18_ Glycosyl hydrolases family 18_ PF00704.15_1.5e-67;fn3_ Fibronectin type III domain_ PF00041.9_7.7e-07;CBM_5_12_ Carbohydrate binding domain_ PF02839.4_1.2e-15;CBM_5_12_ Carbohydrate binding domain_ PF02839.4_2e-14;CBM_5_12_ Carbohydrate binding domain_ PF02839.4_3.6e-15	PS50853_FN3_Fibronectin type III domain profile;PS01095_CHITINASE_18_Chitinases family 18 active site

DOMAINS ASSOCIATED WITH VIRULENCE	FTN_0645	FTT1332	419	-	multidrug/oligosaccharidyl-lipid/polysaccharide (MOP) transporter	no signal peptide detected	COG2244_RfbX_Membrane protein involved in the export of O-antigen and teichoic acid_General function prediction only 3e-09	Polysacc_synt_ Polysaccharide biosynthesis protein_ PF01943.7_0.00015;HlyIII_ Haemolysin-III related PF03006.7 0.086	
DOMAINS ASSOCIATED WITH VIRULENCE	FTN_0664	FTT1314c	194	*fimT*	Type IV pili, pilus assembly protein	signal peptide detected	COG4970_FimT_Tfp pilus assembly protein FimT_Cell motility and secretion / Intracellular trafficking and secretion 3e-07	N_methyl_ Prokaryotic N-terminal methylation motif_ PF07963.1_7.5e-06	PS00409_PROKAR_NTER_METHYL_Prokaryotic N-terminal methylation site
DOMAINS ASSOCIATED WITH VIRULENCE	FTN_0720	FTT0748	238	-	transcriptional regulator, IclR family	no signal peptide detected	COG1414_IclR_Transcriptional regulator_Transcription_3e-31	MarR_ MarR family_ PF01047.10_0.057;Rrf2_ Transcriptional regulator_ PF02082.6_0.014;TrmB_ Sugar-specific transcriptional regulator TrmB_ PF01978.7_0.006;IclR_ Bacterial transcriptional regulator_ PF01614.7_2.4e-07	PS51077_HTH_ICLR_IclR-type HTH domain profile;PS51078_ICLR_ED_IclR effector binding domain profile
EUKARYOTIC DOMAINS	FTN_0745	FTT0571	235	-	protein of unknown function	no signal peptide detected		GST_N_ Glutathione S-transferase, N-terminal domain PF02798.7 0.018	
DOMAINS ASSOCIATED WITH VIRULENCE	FTN_0769	FTT1105c	284	-	peptide methionine sulfoxide reductase	no signal peptide detected	COG0225_MsrA_Peptide methionine sulfoxide reductase _Posttranslational modification, protein turnover, chaperones_5e-49	SelR_ SelR domain_ PF01641.7_2.9e-47;PMSR_ Peptide methionine sulfoxide reductase_ PF01625.8_7.7e-62	
EUKARYOTIC DOMAINS	FTN_0797	FTT0922	182	-	fatty acid hydroxylase	no signal peptide detected	COG3000_ERG3_Sterol desaturase_Lipid metabolism 2e-06	FA_hydroxylase_ Fatty acid hydroxylase_ PF04116.2 2.8e-11	
DOMAINS ASSOCIATED WITH VIRULENCE	FTN_0834	FTT0955c	453	*gor*	glutathione reductase	signal peptide detected	COG1249_Lpd_Pyruvate/2-oxoglutarate dehydrogenase complex, dihydrolipoamide dehydrogenase (E3) component, and related enzymes_Energy production and conversion_1e-113	HI0933_like_HI0933-like protein_PF03486.4_0.011;DAO_FAD dependent oxidoreductase_PF01266.11_0.00084;FAD_binding_2_FAD binding domain_PF00890.13_0.00018;GIDA_Glucose inhibited division protein A_PF01134.11_0.00054;Pyr_redox_2_Pyridine nucleotide-disulphide oxidoreductase_PF07992.2_4.2e-50;NAD_Gly3P_dh_N_NAD-dependent glycerol-3-phosphate dehydrogenase N-terminus_PF01210.12_0.093;Pyr_redox_Pyridine nucleotide-disulphide oxidoreductase_PF00070.16_2.3e-26;Pyr_redox_dim_Pyridine nucleotide-disulphide oxidoreductase, dimerisation domain_PF02852.11_4.2e-52;DAO_ FAD dependent oxidoreductase_ PF01266.10_0.015;Pyr_redox_ Pyridine nucleotide-disulphide oxidoreductase_ PF00070.14_1.1e-71;Pyr_redox_dim_ Pyridine nucleotide-disulphide oxidoreductase, dimerisation domain_ PF02852.9_1.9e-52	PS00076_PYRIDINE_REDOX_1_Pyridine nucleotide-disulphide oxidoreductases class-I active site
EUKARYOTIC DOMAINS	FTN_0856	FTT0976	108	-	thioredoxin	no signal peptide detected	COG3118_COG3118_Thioredoxin domain-containing protein _Posttranslational modification, protein turnover, chaperones_4e-23	Thioredoxin_Thioredoxin_PF00085.8_5.6e-38;Thioredoxin_ Thioredoxin_ PF00085.8_3.7e-37;HyaE_ Hydrogenase-1 expression protein HyaE_ PF07449.1_0.083;DUF836_ Glutaredoxin-like domain (DUF836)_ PF05768.2 0.03	PS00194_THIOREDOXIN_Thioredoxin family active site
DOMAINS ASSOCIATED WITH VIRULENCE	FTN_0861	FTT0980	413	-	conserved protein of unknown function	no signal peptide detected	COG0156_BioF_7-keto-8-aminopelargonate synthetase and related enzymes_Coenzyme metabolism_3e-49	Aminotran_1_2_Aminotransferase class I and II_PF00155.9_0.017;Aminotran_1_2_Aminotransferase class I and II_PF00155.9_0.073;IpaB_EvcA_IpaB/EvcA family_PF03278.3_0.054;Aminotran_1_2_ Aminotransferase class I and II_ PF00155.8 0.011	
DOMAINS ASSOCIATED WITH VIRULENCE	FTN_0877	FTT0997	476	*cls*	cardiolipin synthetase	no signal peptide detected	COG1502_Cls_ Phosphatidylserine/phosphatidylglycerophosphate/cardiolipin synthases and related enzymes_Lipid metabolism_6e-59	DUF843_Baculovirus protein of unknown function (DUF843)_PF05814.1_0.0068;PLDc_Phospholipase D Active site motif_PF00614.11_8.3e-07;PLDc_Phospholipase D Active site motif_PF00614.11_0.00013;DUF843_ Baculovirus protein of unknown function (DUF843)_ PF05814.1_0.063;PLDc_ Phospholipase D Active site motif_ PF00614.9_1.2e-05;PLDc_ Phospholipase D Active site motif_ PF00614.9_0.0022	PS50035_PLD_Phospholipase D phosphodiesterase active site profile;PS50035_PLD_Phospholipase D phosphodiesterase active site profile
DOMAINS ASSOCIATED WITH VIRULENCE	FTN_0918	FTT1040	203	-	conserved protein of unknown function	signal peptide detected	COG4764_COG4764_Uncharacterized protein conserved in bacteria _Function unknown_1e-52	SLT_ Transglycosylase SLT domain_ PF01464.8_0.013	PS00013_PROKAR_LIPOPROTEIN_Prokaryotic membrane lipoprotein lipid attachment site
DOMAINS ASSOCIATED WITH VIRULENCE	FTN_0921	FTT1043	283	-	FKBP-type peptidyl-prolyl cis-trans isomerase	signal peptide detected	COG0545_FkpA_FKBP-type peptidyl-prolyl cis-trans isomerases 1 _Posttranslational modification, protein turnover, chaperones_1e-24	FKBP_C_FKBP-type peptidyl-prolyl cis-trans isomerase_PF00254.16_6.1e-14;FKBP_N_ Domain amino terminal to FKBP-type peptidyl-prolyl isomerase_ PF01346.8_0.0077;FKBP_C_ FKBP-type peptidyl-prolyl cis-trans isomerase_ PF00254.14_3.6e-12	PS00013_PROKAR_LIPOPROTEIN_Prokaryotic membrane lipoprotein lipid attachment site;PS50059_FKBP_PPIASE_FKBP-type peptidyl-prolyl cis-trans isomerase domain profile
DOMAINS ASSOCIATED WITH VIRULENCE	FTN_0922	FTT1044c	325	*ispB*	octaprenyl diphosphate synthase	no signal peptide detected	COG0142_IspA_Geranylgeranyl pyrophosphate synthase_Coenzyme metabolism_5e-76	polyprenyl_synt_Polyprenyl synthetase_PF00348.7_5e-83;FlgM_Anti-sigma-28 factor, FlgM_PF04316.3_0.053;polyprenyl_synt_ Polyprenyl synthetase_ PF00348.7_1.4e-83	PS00723_POLYPRENYL_SYNTHET_1_Polyprenyl synthetases signature 1;PS00444_POLYPRENYL_SYNTHET_2_Polyprenyl synthetases signature 2
DOMAINS ASSOCIATED WITH VIRULENCE	FTN_0946	FTT1057c	301	*pilF*	Type IV pili, pilus assembly protein	no signal peptide detected	COG3063_PilF_Tfp pilus assembly protein PilF_Cell motility and secretion / Intracellular trafficking and secretion_4e-15	TPR_2_Tetratricopeptide repeat_PF07719.4_0.0017;TPR_2_ Tetratricopeptide repeat_ PF07719.2_0.03	PS00013_PROKAR_LIPOPROTEIN_Prokaryotic membrane lipoprotein lipid attachment site;PS50293_TPR_REGION_TPR repeat region circular profile
DOMAINS ASSOCIATED WITH VIRULENCE	FTN_0957	FTT0558	203	-	short chain dehydrogenase	no signal peptide detected	COG1028_FabG_Dehydrogenases with different specificities (related to short-chain alcohol dehydrogenases)_Secondary metabolites biosynthesis, transport, and catabolism / General function prediction only_5e-10	adh_short_short chain dehydrogenase_PF00106.13_0.084;DapB_N_Dihydrodipicolinate reductase, N-terminus_PF01113.10_0.0083;RmlD_sub_bind_RmlD substrate binding domain_PF04321.6_0.005;Epimerase_NAD dependent epimerase/dehydratase family_PF01370.10_7.6e-06;NmrA_NmrA-like family_PF05368.2_0.041;Saccharop_dh_Saccharopine dehydrogenase_PF03435.7_0.021;adh_short_ short chain dehydrogenase_ PF00106.12 0.00056	
DOMAINS ASSOCIATED WITH VIRULENCE	FTN_0959	FTT0556c	289	*oxyR*	oxidative stress transcriptional regulator	no signal peptide detected	COG0583_LysR_Transcriptional regulator_Transcription_3e-36	HTH_1_Bacterial regulatory helix-turn-helix protein, lysR family_PF00126.14_4.5e-14;LysR_substrate_LysR substrate binding domain_PF03466.8_5.3e-30;HTH_1_ Bacterial regulatory helix-turn-helix protein, lysR family_ PF00126.12_1.8e-14;LysR_substrate_ LysR substrate binding domain_ PF03466.6_1.5e-27	PS50931_HTH_LYSR_LysR-type HTH domain profile
DOMAINS ASSOCIATED WITH VIRULENCE	FTN_0977	FTT0538c	189	-	conserved protein of unknown function	signal peptide detected	COG4764_COG4764_Uncharacterized protein conserved in bacteria _Function unknown_6e-24	SLT_Transglycosylase SLT domain_PF01464.8_6.2e-09;SLT_ Transglycosylase SLT domain_ PF01464.8 0.00017	PS00013_PROKAR_LIPOPROTEIN_Prokaryotic membrane lipoprotein lipid attachment site
EUKARYOTIC DOMAINS	FTN_0994	FTT0689	249	-	hypothetical membrane protein	no signal peptide detected			

DOMAINS ASSOCIATED WITH VIRULENCE	FTN_0999	FTT0684c	466	*udhA*	soluble pyridine nucleotide transhydrogenase	no signal peptide detected	COG1249_Lpd_Pyruvate/2-oxoglutarate dehydrogenase complex, dihydrolipoamide dehydrogenase (E3) component, and related enzymes_Energy production and conversion_2e-108	HI0933_like_HI0933-like protein_PF03486.4_0.0069;DAO_FAD dependent oxidoreductase_PF01266.11_0.002;FAD_binding_2_FAD binding domain_PF00890.13_5.2e-06;Pyr_redox_2_Pyridine nucleotide-disulphide oxidoreductase_PF07992.2_4.3e-43;Pyr_redox_Pyridine nucleotide-disulphide oxidoreductase_PF00070.16_1.3e-21;Pyr_redox_dim_Pyridine nucleotide-disulphide oxidoreductase, dimerisation domain_PF02852.11_2.5e-32;DAO_ FAD dependent oxidoreductase_ PF01266.10_0.069;Pyr_redox_ Pyridine nucleotide-disulphide oxidoreductase_ PF00070.14_2.8e-63;Pyr_redox_dim_ Pyridine nucleotide-disulphide oxidoreductase, dimerisation domain_ PF02852.9_1.3e-32	
DOMAINS ASSOCIATED WITH VIRULENCE	FTN_1052	FTT0629	308	*miaA*	tRNA delta(2)-isopentenylpyrophosphate transferase	no signal peptide detected	COG0324_MiaA_tRNA delta(2)-isopentenylpyrophosphate transferase_Translation, ribosomal structure and biogenesis_2e-89	AAA_ATPase family associated with various cellular activities (AAA)_PF00004.17_0.037;IPT_Isopentenyl transferase_PF01745.6_0.021;IPPT_IPP transferase_PF01715.7_3.1e-105;IPT_ Isopentenyl transferase_ PF01745.6_0.048;IPPT_ IPP transferase_ PF01715.6_7.1e-106	
DOMAINS ASSOCIATED WITH VIRULENCE	FTN_1058	FTT0623	438	*tig*	trigger factor (TF) protein (peptidyl-prolyl cis/trans isomerase)	no signal peptide detected	COG0544_Tig_FKBP-type peptidyl-prolyl cis-trans isomerase (trigger factor)_Posttranslational modification, protein turnover, chaperones_1e-81	Trigger_N_Bacterial trigger factor protein (TF)_PF05697.3_2.1e-53;FKBP_C_FKBP-type peptidyl-prolyl cis-trans isomerase_PF00254.16_1.1e-25;Trigger_C_Bacterial trigger factor protein (TF) C-terminus_PF05698.3_2e-28;Trigger_N_ Bacterial trigger factor protein (TF)_ PF05697.2_5.2e-54;FKBP_C_ FKBP-type peptidyl-prolyl cis-trans isomerase_ PF00254.14_1.3e-24;Trigger_C_ Bacterial trigger factor protein (TF) C-terminus_ PF05698.2_1.5e-28	PS50059_FKBP_PPIASE_FKBP-type peptidyl-prolyl cis-trans isomerase domain profile
EUKARYOTIC DOMAINS	FTN_1074	FTT0609	586	-	X-prolyl aminopeptidase 2	no signal peptide detected	COG0006_PepP_Xaa-Pro aminopeptidase_Amino acid transport and metabolism_1e-52	Peptidase_M24_metallopeptidase family M24_PF00557.12_6.2e-11;Peptidase_M24_ metallopeptidase family M24_ PF00557.10_2.8e-10	
DOMAINS ASSOCIATED WITH VIRULENCE	FTN_1137	FTT1156c	594	*pilQ*	Type IV pili secretin component	signal peptide detected	COG4796_HofQ_Type II secretory pathway, component HofQ _Intracellular trafficking and secretion_6e-68	STN_Secretin and TonB N terminus short domain_PF07660.3_5e-06;Secretin_N_Bacterial type II/III secretion system short domain_PF03958.6_1.2e-13;Secretin_Bacterial type II and III secretion system protein_PF00263.10_3.2e-64;PLRV_ORF5_Potato leaf roll virus readthrough protein_PF01690.6_0.013;STN_ Secretin and TonB N terminus short domain_ PF07660.2_0.00038;Secretin_N_ Bacterial type II/III secretion system short domain_ PF03958.6_6.6e-13;Secretin_ Bacterial type II and III secretion system protein_ PF00263.9_7.6e-65	PS00875_T2SP_D_Bacterial type II secretion system protein D signature
EUKARYOTIC DOMAINS	FTN_1151	FTT1170	582	-	conserved protein of unknown function	no signal peptide detected	COG0790_COG0790_FOG: TPR repeat, SEL1 subfamily_General function prediction only_2e-06	Sel1_Sel1 repeat_PF08238.1_5.4e-06;Sel1_Sel1 repeat_PF08238.1_7.5e-08;DUF928_Domain of Unknown Function (DUF928)_PF06051.2_0.043;Sel1_Sel1 repeat_PF08238.1_0.0013;TPR_1_Tetratricopeptide repeat_PF00515.15_0.069;Sel1_Sel1 repeat_PF08238.1_6.8e-08	
EUKARYOTIC DOMAINS	FTN_1184	FTT1207	456	-	hypothetical membrane protein	signal peptide detected		7tm_5_ 7TM chemoreceptor_ PF01604.11_0.096	PS50267_NA_NEUROTRAN_SYMP_3_Sodium:neurotransmitter symporter family profile
EUKARYOTIC DOMAINS	FTN_1187	FTT0821	234	-	protein of unknown function	no signal peptide detected	COG0398_COG0398_Uncharacterized conserved protein_Function unknown_1e-08	DedA_DedA family_PF00597.9_2.4e-10;DedA_ DedA family_ PF00597.8_1.1e-05	
DOMAINS ASSOCIATED WITH VIRULENCE	FTN_1219	FTT0791	339	*galE*	UDP-glucose 4-epimerase	no signal peptide detected	COG1087_GalE_UDP-glucose 4-epimerase_Cell envelope biogenesis, outer membrane_1e-130	Epimerase_NAD dependent epimerase/dehydratase family_PF01370.10_2.1e-76;NmrA_NmrA-like family_PF05368.2_0.003;Polysacc_synt_2_Polysaccharide biosynthesis protein_PF02719.5_0.00049;Saccharop_dh_Saccharopine dehydrogenase_PF03435.7_0.0003;3Beta_HSD_3-beta hydroxysteroid dehydrogenase/isomerase family_PF01073.8_8.3e-10;NAD_binding_4_Male sterility protein_PF07993.1_0.048;3Beta_HSD_ 3-beta hydroxysteroid dehydrogenase/isomerase family_ PF01073.7_1.4e-08;adh_short_ short chain dehydrogenase_ PF00106.12_0.0073;Epimerase_ NAD dependent epimerase/dehydratase family_ PF01370.8_1.8e-153	
DOMAINS ASSOCIATED WITH VIRULENCE	FTN_1268	FTT1249	241	-	conserved protein of unknown function	no signal peptide detected	COG1463_Ttg2C_ABC-type transport system involved in resistance to organic solvents, periplasmic component_Secondary metabolites biosynthesis, transport, and catabolism_6e-19	MCE_mce related protein_PF02470.9_3.7e-17;MCE_ mce related protein_ PF02470.8_1.3e-09	
DOMAINS ASSOCIATED WITH VIRULENCE	FTN_1274	FTT1255c	275	-	transcriptional regulator, AraC family	no signal peptide detected	COG2207_AraC_AraC-type DNA-binding domain-containing proteins _Transcription_4e-21	HTH_AraC_Bacterial regulatory helix-turn-helix proteins, AraC family_PF00165.10_7.2e-06;HTH_AraC_Bacterial regulatory helix-turn-helix proteins, AraC family_PF00165.10_3.2e-09;HTH_AraC_ Bacterial regulatory helix-turn-helix proteins, AraC family_ PF00165.8_0.00018;HTH_AraC_ Bacterial regulatory helix-turn-helix proteins, AraC family_ PF00165.8_2.6e-07	PS00041_HTH_ARAC_FAMILY_1_"Bacterial regulatory proteins, araC family signature";PS01124_HTH_ARAC_FAMILY_2_"Bacterial regulatory proteins, araC family DNA-binding domain profile"
DOMAINS ASSOCIATED WITH VIRULENCE	FTN_1287	FTT1272	394	*hemG*	protoporphyrinogen oxidase	no signal peptide detected	COG1232_HemY_Protoporphyrinogen oxidase_Coenzyme metabolism_3e-20	GMC_oxred_N_GMC oxidoreductase_PF00732.9_0.011;DAO_FAD dependent oxidoreductase_PF01266.11_0.00024;Lycopene_cycl_Lycopene cyclase protein_PF05834.2_0.00097;TrkA_N_TrkA-N domain_PF02254.7_0.042;Amino_oxidase_Flavin containing amine oxidoreductase_PF01593.12_0.00081	
DOMAINS ASSOCIATED WITH VIRULENCE	FTN_1319	FTT1709	1325	*pdpC*	hypothetical protein				
DOMAIN ASSOCIATED WITH TOXIN SYNTHESIS	FTN_1340	FTT1376	94	*acpP*	acyl carrier protein	no signal peptide detected	COG0236_AcpP_Acyl carrier protein_Lipid metabolism / Secondary metabolites biosynthesis, transport, and catabolism_2e-13	PP-binding_Phosphopantetheine attachment site_PF00550.12_5.1e-20;PP-binding_ Phosphopantetheine attachment site_ PF00550.10_3.6e-20	PS00012_PHOSPHOPANTETHEINE_Phosphopantetheine attachment site;PS50075_ACP_DOMAIN_Acyl carrier protein phosphopantetheine domain profile
DOMAINS ASSOCIATED WITH VIRULENCE	FTN_1355	FTT1392	256	-	regulatory factor, Bvg accessory factor family	no signal peptide detected	COG1521_COG1521_Putative transcriptional regulator, homolog of Bvg accessory factor_Transcription_1e-52	Bvg_acc_factor_Bordetella pertussis Bvg accessory factor family_PF03309.3_7.9e-60;Bvg_acc_factor_ Bordetella pertussis Bvg accessory factor family_ PF03309.3_2.1e-60	

EUKARYOTIC DOMAINS	FTN_1415	FTT1445	107	-	thioredoxin	no signal peptide detected	COG3118_COG3118_Thioredoxin domain-containing protein _Posttranslational modification, protein turnover, chaperones_6e-25	Thioredoxin_Thioredoxin_PF00085.8_4.5e-38;Glutaredoxin_Glutaredoxin_PF00462.11_0.0052;DUF836_Glutaredoxin-like domain (DUF836)_PF05768.3_0.019;Thioredoxin_ Thioredoxin_ PF00085.8_2e-37;DUF836_ Glutaredoxin-like domain (DUF836)_ PF05768.2_0.1	PS00194_THIOREDOXIN_Thioredoxin family active site
DOMAINS ASSOCIATED WITH VIRULENCE	FTN_1425	FTT1459c	324	*wbtF*	NAD dependent epimerase	no signal peptide detected	COG0451_WcaG_Nucleoside-diphosphate-sugar epimerases_Cell envelope biogenesis, outer membrane / Carbohydrate transport and metabolism_6e-58	Epimerase_NAD dependent epimerase/dehydratase family_PF01370.10_5.9e-73;NmrA_NmrA-like family_PF05368.2_0.025;Polysacc_synt_2_Polysaccharide biosynthesis protein_PF02719.5_0.087;3Beta_HSD_3-beta hydroxysteroid dehydrogenase/isomerase family_PF01073.8_2.1e-25;NAD_binding_4_Male sterility protein_PF07993.1_0.0053;Polysacc_synt_2_Polysaccharide biosynthesis protein_PF02719.5_0.082;Polysacc_synt_2_Polysaccharide biosynthesis protein_PF02719.5_0.00048;3Beta_HSD_ 3-beta hydroxysteroid dehydrogenase/isomerase family_ PF01073.7_5.8e-13;adh_short_ short chain dehydrogenase_ PF00106.12_0.014;Epimerase_ NAD dependent epimerase/dehydratase family_ PF01370.8_6e-116	PS00867_CPSASE_2_Carbamoyl-phosphate synthase subdomain signature 2
DOMAINS ASSOCIATED WITH VIRULENCE	FTN_1449	FTT1540c	202	-	conserved protein of unknown function	signal peptide detected	COG3417_FlgN_Collagen-binding surface adhesin SpaP (antigen I/II family)_General function prediction only_8e-16	Entericidin_Entericidin EcnA/B family_PF08085.1_0.084;Mycoplasma_p37_High affinity transport system protein p37_PF06646.1_0.079	PS00013_PROKAR_LIPOPROTEIN_Prokaryotic membrane lipoprotein lipid attachment site
DOMAINS ASSOCIATED WITH VIRULENCE	FTN_1461	FTT1553c	765	*rnr*	ribonuclease R	no signal peptide detected	COG0557_VacB_Exoribonuclease R_Transcription_7e-161	RNB_RNB-like protein_PF00773.8_7.7e-102;S1_S1 RNA binding domain_PF00575.11_0.0047;RNB_ RNB-like protein_ PF00773.7_1e-105;S1_ S1 RNA binding domain_ PF00575.10_0.0065	PS50126_S1_S1 domain profile;PS01175_RIBONUCLEASE_II_Ribonuclease II family signature
DOMAINS ASSOCIATED WITH VIRULENCE	FTN_1470	FTT1562	293	*ispA*	geranyl diphosphate synthase/farnesyl diphosphate synthase	no signal peptide detected	COG0142_IspA_Geranylgeranyl pyrophosphate synthase_Coenzyme metabolism_6e-53	MNHE_Na+/H+ ion antiporter subunit_PF01899.6_0.018;polyprenyl_synt_Polyprenyl synthetase_PF00348.7_3e-83;DUF1477_Protein of unknown function (DUF1477)_PF07346.1_0.044;polyprenyl_synt_ Polyprenyl synthetase_ PF00348.7_3.2e-81	PS00723_POLYPRENYL_SYNTHET_1_Polyprenyl synthetases signature 1;PS00444_POLYPRENYL_SYNTHET_2_Polyprenyl synthetases signature 2
DOMAINS ASSOCIATED WITH VIRULENCE	FTN_1480	FTT1571c	337	*lpxD*	UDP-3-O-(3-hydroxy-fatty acid)-glucosamine N-acyltransferase	no signal peptide detected	COG1044_LpxD_UDP-3-O-[3-hydroxymyristoyl]__3e-87	LpxD_UDP-3-O-[3-hydroxymyristoyl] glucosamine N-acyltransferase, LpxD_PF04613.3_5.4e-31;Hexapep_Bacterial transferase hexapeptide (three repeats)_PF00132.11_6.8e-05;Hexapep_Bacterial transferase hexapeptide (three repeats)_PF00132.11_0.00057;Hexapep_Bacterial transferase hexapeptide (three repeats)_PF00132.11_0.0025;Hexapep_Bacterial transferase hexapeptide (three repeats)_PF00132.11_0.00044;Hexapep_Bacterial transferase hexapeptide (three repeats)_PF00132.11_0.011;Hexapep_Bacterial transferase hexapeptide (three repeats)_PF00132.11_0.045;Hexapep_Bacterial transferase hexapeptide (three repeats)_PF00132.11_0.012;LpxD_ UDP-3-O-[3-hydroxymyristoyl] glucosamine N-acyltransferase, LpxD_ PF04613.2_8.9e-31;Hexapep_ Bacterial transferase hexapeptide (three repeats)_ PF00132.10_0.0015;Hexapep_ Bacterial transferase hexapeptide (three repeats)_ PF00132.10_0.013;Hexapep_ Bacterial transferase hexapeptide (three repeats)_ PF00132.10_0.057;Hexapep_ Bacterial transferase hexapeptide (three repeats)_ PF00132.10_0.0098	PS00101_HEXAPEP_TRANSFERASES_Hexapeptide-repeat containing-transferases signature;PS00101_HEXAPEP_TRANSFERASES_Hexapeptide-repeat containing-transferases signature
DOMAINS ASSOCIATED WITH VIRULENCE	FTN_1492	FTT1483c	470	*lpdA*	pyruvate dehydrogenase complex, E3 component, dihydrolipoamide dehydrogenase	no signal peptide detected	COG1249_Lpd_Pyruvate/2-oxoglutarate dehydrogenase complex, dihydrolipoamide dehydrogenase (E3) component, and related enzymes_Energy production and conversion_3e-134	FAD_binding_3_FAD binding domain_PF01494.8_4.7e-05;HI0933_like_HI0933-like protein_PF03486.4_0.042;DAO_FAD dependent oxidoreductase_PF01266.11_0.0021;GIDA_Glucose inhibited division protein A_PF01134.11_0.00045;Pyr_redox_2_Pyridine nucleotide-disulphide oxidoreductase_PF07992.2_1.4e-64;Pyr_redox_Pyridine nucleotide-disulphide oxidoreductase_PF00070.16_9.1e-23;Pyr_redox_dim_Pyridine nucleotide-disulphide oxidoreductase, dimerisation domain_PF02852.11_4e-49;DAO_ FAD dependent oxidoreductase_ PF01266.10_0.035;Pyr_redox_ Pyridine nucleotide-disulphide oxidoreductase_ PF00070.14_3.1e-88;FAD_binding_3_ FAD binding domain_ PF01494.7_0.016;Pyr_redox_dim_ Pyridine nucleotide-disulphide oxidoreductase, dimerisation domain_ PF02852.9_2.6e-49	PS00076_PYRIDINE_REDOX_1_Pyridine nucleotide-disulphide oxidoreductases class-I active site
DOMAINS ASSOCIATED WITH VIRULENCE	FTN_1506	FTT1496c	337	-	pilus assembly protein	signal peptide detected	COG4966_PilW_Tfp pilus assembly protein PilW_Cell motility and secretion / Intracellular trafficking and secretion_9e-06	N_methyl_Prokaryotic N-terminal methylation motif_PF07963.2_2.5e-06;RNA_pol_Rpb1_3_RNA polymerase Rpb1, domain 3_PF04983.7_0.038;N_methyl_ Prokaryotic N-terminal methylation motif_ PF07963.1_1.4e-05	PS00409_PROKAR_NTER_METHYL_Prokaryotic N-terminal methylation site
EUKARYOTIC DOMAINS	FTN_1552	FTT0161c	208	-	acid phosphatase, PAP2 family	no signal peptide detected		PAP2_PAP2 superfamily_PF01569.11_3.8e-16;PAP2_ PAP2 superfamily_ PF01569.9_6.5e-07	
DOMAINS ASSOCIATED WITH VIRULENCE	FTN_1603	FTT0112	258	-	regulatory factor, Bvg accessory factor family	no signal peptide detected	COG1521_COG1521_Putative transcriptional regulator, homolog of Bvg accessory factor_Transcription_6e-45	Bvg_acc_factor_ Bordetella pertussis Bvg accessory factor family_ PF03309.3_6.8e-40	
DOMAINS ASSOCIATED WITH VIRULENCE	FTN_1681	FTT0030c	140	*fur*	ferric uptake regulation protein	no signal peptide detected	COG0735_Fur_Fe2+/Zn2+ uptake regulation proteins_Inorganic ion transport and metabolism_1e-31	FUR_ Ferric uptake regulator family_ PF01475.6_1.8e-49;MarR_ MarR family_ PF01047.10_0.072	
DOMAINS ASSOCIATED WITH VIRULENCE	FTN_1768	FTT1793c	858	*pepN*	aminopeptidase N	no signal peptide detected	COG0308_PepN_Aminopeptidase N_Amino acid transport and metabolism_1e-167	Peptidase_M1_ Peptidase family M1_ PF01433.9_1.4e-70	PS00142_ZINC_PROTEASE_"Neutral zinc metallopeptidases, zinc-binding region signature"
DOMAINS ASSOCIATED WITH VIRULENCE	FTN_1772	FTT1797c	229	-	peptide methionine sulfoxide reductase	signal peptide detected	COG0225_MsrA_Peptide methionine sulfoxide reductase _Posttranslational modification, protein turnover, chaperones_3e-49	PMSR_ Peptide methionine sulfoxide reductase_ PF01625.8_6.1e-63	
