# Supplementary material for: Comparison of Francisella tularensis genomes reveals evolutionary events associated with the emergence of human pathogenic strains
Source: Genome Biol. 2007 Jun 5;8(6):R102. doi: 10.1186/gb-2007-8-6-r102 (PMC2394750; doi:10.1186/gb-2007-8-6-r102)
Supplement: Additional data file 3 — Provided is a list of the duplicated genes (100% identity) in the genomes of Francisella tularensis subspecies tularensis Schu S4 and Francisella tularensis subspecies holarctica LVS, and their counterpart in Francisella tularensis subspecies novicida U112. [file gb-2007-8-6-r102-S3.pdf]

Supplemental Table 3: List of the duplicated genes (100% identity) in the genomes of *F.t. tularensis* Schu S4 and *F.t. holarctica* LVS, and their counterpart in *F.t. novicida* U112

Locus tags for genes in U112[1]	Locus tags of orthologs in Schu S4[1]			Locus tags for orthologs in LVS[1]		
-	FTT0354	FTT0378c	FTT1263c	FTL_0265	FTL_1317	-
FTN_0487	FTT0390c	-	-	FTL_0456	FTL_1047	-
FTN_0488	FTT0391c	-	-	FTL_0457	FTL_1361	-
FTN_0667	FTT1311	-	-	(FTL_1465)	(FTL_1469)	-
FTN_0667	FTT1311	-	-	(FTL_1466)	(FTL_1470)	(FTL_1445)
FTN_0668	FTT1310c	-	-	FTL_1464	FTL_1468	-
FTN_0987	FTT0519	-	-	FTL_0975	FTL_1549	-
FTN_1309	FTT1344	FTT1699	-	FTL_0126	FTL_1172	-
FTN_1310	FTT1345	FTT1700	-	FTL_0125	FTL_1171	-
FTN_1311	FTT1346	FTT1701	-	FTL_0124	FTL_1170	-
FTN_1312	FTT1347	FTT1702	-	FTL_0123	FTL_1169	-
FTN_1313	FTT1348	FTT1703	-	FTL_0122	FTL_1168	-
FTN_1314	FTT1349	FTT1704	-	FTL_0121	FTL_1167	-
FTN_1315	FTT1350	FTT1705	-	FTL_0120	FTL_1166	-
FTN_1316	FTT1351	FTT1706	-	FTL_0119	FTL_1165	-
FTN_1317	FTT1352	FTT1707	-	FTL_0118	FTL_1164	-
FTN_1318	FTT1353	FTT1708	-	FTL_0117	FTL_1163	-
FTN_1319	FTT1354	FTT1709	-	FTL_0116	FTL_1162	-
FTN_1320	FTT1355	FTT1710	-	FTL_0115	FTL_1161	-
FTN_1321	FTT1356c	FTT1711c	-	FTL_0114	FTL_1160	-
FTN_1322	FTT1357c	FTT1712c	-	FTL_0113	FTL_1159	-
FTN_1323	FTT1358c	FTT1713c	-	FTL_0112	FTL_1158	-
FTN_1324	FTT1359c	FTT1714c	-	FTL_0111	FTL_1157	-
FTN_1325	FTT1360c	FTT1715c	-	(FTL_0109)	(FTL_1155)	-
FTN_1326	(FTT1361c)	(FTT1716c)	-	-	-	-
FTN_1513	FTT1503	-	-	FTL_0290	FTL_1734	FTL_1892
FTN_1520	FTT1510c	-	-	FTL_0283	FTL_1875	-
FTN_1549	FTT0164c	-	-	FTL_1725	FTL_1868	-

[1] The locus tags of pseudogenes have been put between parenthesis.
